# Animal Models to Understand the Etiology and Pathophysiology of Polycystic Ovary Syndrome

**DOI:** 10.1210/endrev/bnaa010

**Published:** 2020-04-20

**Authors:** Elisabet Stener-Victorin, Vasantha Padmanabhan, Kirsty A Walters, Rebecca E Campbell, Anna Benrick, Paolo Giacobini, Daniel A Dumesic, David H Abbott

**Affiliations:** 1 Department of Physiology and Pharmacology, Karolinska Institutet, Stockholm, Sweden; 2 Departments of Pediatrics, Obstetrics and Gynecology, and Environmental Health Sciences, University of Michigan, Ann Arbor, Michigan; 3 Fertility & Research Centre, School of Women’s and Children’s Health, University of New South Wales, Sydney, New South Wales, Australia; 4 Centre for Neuroendocrinology and Department of Physiology, School of Biomedical Sciences, University of Otago, Dunedin, New Zealand; 5 Department of Physiology, Sahlgrenska Academy, University of Gothenburg, Gothenburg, Sweden; 6 School of Health Sciences and Education, University of Skövde, Skövde, Sweden; 7 University of Lille, Inserm, CHU Lille, U1172 - LilNCog - Lille Neuroscience & Cognition, F-59000 Lille, France; 8 Department of Obstetrics and Gynecology, David Geffen School of Medicine, University of California, California; 9 Department of Obstetrics and Gynecology, Wisconsin National Primate Research Center, University of Wisconsin, Madison, Wisconsin

**Keywords:** androgen excess, developmental programming, genetic manipulation, therapeutic prevention, adipogenic constraint-induced lipotoxicity, naturally hyperandrogenic female monkeys

## Abstract

More than 1 out of 10 women worldwide are diagnosed with polycystic ovary syndrome (PCOS), the leading cause of female reproductive and metabolic dysfunction. Despite its high prevalence, PCOS and its accompanying morbidities are likely underdiagnosed, averaging > 2 years and 3 physicians before women are diagnosed. Although it has been intensively researched, the underlying cause(s) of PCOS have yet to be defined. In order to understand PCOS pathophysiology, its developmental origins, and how to predict and prevent PCOS onset, there is an urgent need for safe and effective markers and treatments. In this review, we detail which animal models are more suitable for contributing to our understanding of the etiology and pathophysiology of PCOS. We summarize and highlight advantages and limitations of hormonal or genetic manipulation of animal models, as well as of naturally occurring PCOS-like females.

## Graphical Abstract

**Graphical Abstract:**
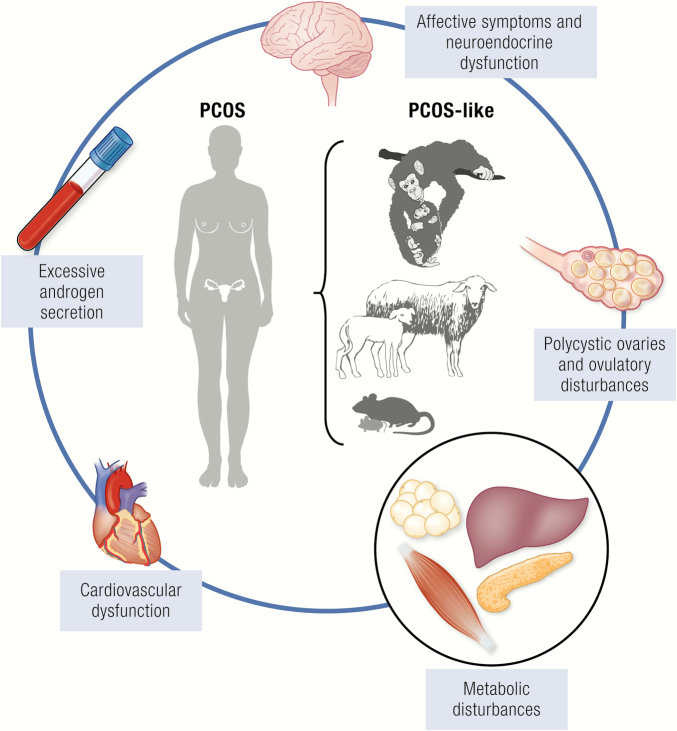

**Essential Points**

- Worldwide, 15% to 20% of women in their reproductive years have polycystic ovary syndrome (PCOS), contributing to an annual multibillion-dollar health care burden, but progress towards a cure is hindered by an absence of detailed mechanistic understanding.
- With PCOS risk genes accounting for < 10% of PCOS cases, and its heritability of 70%, PCOS etiopathogenesis may comprise complex genetic, epigenetic, and developmental contributions, including hormonally and metabolically compromised maternal environment.
- Nonhuman primate models of maternal androgen exposure or peripubertal T-onset most closely emulate PCOS pathophysiology and its metabolic sequelae, and recent work with naturally hyperandrogenic PCOS-like female rhesus macaques, with > 95% exome homology to humans, promises whole-genome sequence insight into genetic-based origins.
- Sheep models of maternal androgen exposure, providing relatively inexpensive ease of incisive developmental and pharmacological manipulation, engage rapidly maturing, mono-ovular, large-bodied females in preventive strategies demonstrating the strengths and weaknesses of fetal and postnatal intervention.
- Rodent maternal androgen or anti-Müllerian hormone (AMH) exposure or peripubertal dihydrotestosterone (DHT)-exposure, combined with genetically manipulated mouse models, will provide unique insights into how androgen receptors provide molecular gateways towards PCOS etiopathogenesis.
- Maternal androgen exposure rat and mice models demonstrate a molecular, neural blueprint for fetal programming of anxiety and depression accompanying PCOS, and how acupuncture counteracts PCOS-like traits.
- Although animal models of PCOS have their limitations, use of appropriate animal model(s) is enabling discovery, validation, and optimization of novel biomarkers and treatments for women with PCOS.

Polycystic ovary syndrome (PCOS) has drastic, lifelong consequences for a woman’s health and wellbeing (1, 2). Androgen excess is the most common endocrinopathy of PCOS, with > 80% prevalence. Newly published International Guidelines for the Assessment and Management of PCOS (1) endorse a clinical diagnosis requiring at least 2 out of the following 3 (Rotterdam) criteria: (i) high circulating levels of testosterone (T) or excessive body hair (hirsutism); (ii) intermittent or absent menstrual cycles; (iii) and polycystic ovaries on ultrasound, provided that related, but distinctly different, endocrine disorders have been excluded (3). Rotterdam criteria generate 4 PCOS phenotypes: Classic PCOS with **type A**, hyperandrogenism or hirsutism (HA) + intermittent/absent cycles (ovulatory dysfunction, OD) + polycystic ovary morphology (PCOM), and **type B**, HA + OD; **type C**, HA + PCOM; and **type D**, OD + PCOM. Phenotype B, however, may eventually be reassigned to Type A when three-dimensional ovarian imaging and standardized anti-Müllerian hormone (AMH) assays become widely available, enabling accurate characterization of a woman’s ovarian follicle population (4).

PCOS is strongly familial (5-7) and highly heritable (8), with approximately 60% to 70% of daughters born to women with PCOS manifesting their own PCOS phenotype during adolescence and as young adults (9, 10). Hyperandrogenism is the most heritable phenotypic trait (11). Using the Rotterdam PCOS criteria, prevalence rates as high as 21% are reported across a variety of populations (12-14). Moreover, prevalence rates increase to over 25% in severely obese women with PCOS (15), and significant morbidity accompanies known associations with type 2 diabetes (T2D), cardiovascular dysfunction, obesity, infertility and cancer (2, 14, 16); hence, PCOS places a heavy burden on healthcare resources (17). Yet PCOS and its accompanying morbidities remain underdiagnosed, with an average of more than 2 years and 3 physicians before women are diagnosed (1).

Commonly, onset of PCOS-related symptoms occurs during adolescence, but can be delayed into a woman’s reproductive years. Signs of PCOS, however, are found before pubertal onset of its clinical phenotype. Daughters born to mothers with PCOS have a 5-fold increased risk of developing PCOS themselves (9, 10, 18) and, as newborn infants, exhibit elongated anogenital distance (AGD) (19) and facial sebum (20), with elevated circulating levels of ovarian AMH (21, 22) indicative of exaggerated antral follicle numbers. Progress towards a cure for PCOS, however, has been mostly hindered by the absence of reliable, pre-PCOS biomarkers during infancy or childhood, a defining mechanistic pathogenesis, evolving diagnostic criteria, and readily available, naturally-occurring or experimentally-induced animal models encompassing the complexity of PCOS and its multiple phenotypes (23, 24). Since currently identified PCOS risk genes account for < 10% of PCOS prevalence (16, 25), etiopathogenesis is likely a combination of polygenic, epigenetic and developmental contributions (3, 16, 23, 25, 26), exaggerated by obesity or ameliorated by lifestyle (15, 27, 28). Animal models have indeed contributed to increased understanding of PCOS etiopathogenesis and underlying pathophysiological mechanisms, expanding from fewer than 10 publications in the 1970s to approximately 400 in the 2010s (data obtained from PubMed January 21, 2020). Most employ a variety of discrete or continuing experimental manipulations to alter phenotype due to programmed (organizational) and/or activational contribution to the manifestation and severity of pathophysiology, respectively (23, 24, 29-34). It is important to emphasize, however, that in contrast to discretely-timed gestational manipulations that permanently reorganize organ and tissue structure and function, peripubertal or adult onset manipulations that persistently activate structural and functional changes have yet to demonstrate lifelong persistence when manipulations cease. Increasing numbers of elegant, genetically manipulated rodent models are providing unparalleled insight into molecular understanding (Table 3) (35).

In addition, animal models exhibiting traits sufficiently reminiscent of a single Rotterdam criterion for PCOS in women—such as elevated circulating levels of T (total or unbound) compared to species-relevant controls or control populations—are considered to express a PCOS-like trait, but are not sufficiently PCOS-like *per se* (Supplementary Table 1a, b) (36). There are no species-specific, clinical veterinarian diagnoses for PCOS, hence nonhuman species cannot exhibit PCOS by definition. Animal models, nevertheless, can exhibit a combination of 2 or more PCOS-like equivalents of Rotterdam criteria that qualify their inclusion as PCOS-like, providing potentially more relevance for etiopathogenic studies (Tables 1-3). These include the more recently reported, naturally hyperandrogenic female found in nonhuman primates (NHP), namely cynomolgus and rhesus macaques (37), in which spontaneously hyperandrogenic females exhibit PCOS-like diagnostic traits together with additional neuroendocrine, ovarian, and metabolic traits that commonly accompany PCOS in women (Supplementary Table 3 (36). Animal models described in Tables 1-3 stand in contrast to those described in Supplementary Table 1a and 1b (36); these models either exhibit PCOS-like traits alongside other PCOS-excluding endocrinopathies, such as hyperprolactinemia and hypogonadotropic amenorrhea, including luteinizing hormone (LH)-β overexpressing mice and dehydroepiandrosterone (DHEA)-treated prepubertal or adult female rodents (32, 38, 39), or less than two traits approximating biomarkers for Rotterdam equivalent criteria, such as T-treated neonatal rats (38, 40). These latter models either do not qualify as PCOS-like *per se* or express traits that may compromise their mechanistic usefulness in investigations of PCOS etiopathogenesis.

**Table 1.** Prenatal Manipulations Generating PCOS-like First-Generation Offspring

Species		NHP		Sheep		Rat		Mouse	
**Treatment**	**Testosterone propionate**	**Testosterone propionate**	**DHT propionate**	**T**	**DHT**	**T**	**DHT**	**DHT**	**AMHc or proAMH**
**Age of treatment**	GD 40-80	GD 110-140	GD 40-95/100	GD 30-90 GD 60-90	GD 30-90	E15-19	E16-19	E16.5-18.5 / E0-delivery	E16.5-E18.5
**Dose of treatment**	10-15mg (=1.4-2.1 mg/kg) daily s.c.	10mg (=1.4 mg/kg) daily s.c.	10-15mg (=1.4-2.1 mg/kg) daily s.c.	100mg (=1.2 mg/kg) twice weekly i.m.	100mg (=1.2 mg/kg) twice weekly i.m.	0.5 mg/kg/day s.c	3 mg/day s.c.	250ug	0.12 mg/kg daily (i.p.)
**Duration of treatment**	15-40 days	25-30 days	55-60 days	30, 60 days	60 days	5 days	4 days	3 days	3 days
**Traits approximating or biomarkers for criteria used for PCOS diagnosis in women (at least 2 out of 3 required)**									
Intermittent or absent ovulatory cycles	✔	✔	✔	✔	✔	✔	✔	✔	✔
Elevated endogenous androgen levels or biomarkers of elevated androgens	✔	✔		✔	✖	✔/✖	✔/✖	✔/ ✖	✔
Polyfollicular ovaries	✔			✔	✖			✖	✖
**Traits accompanying PCOS**									
**1. Ovary**									
↑ Ovary weight or size	✔			✖	✖	✖		✖	✖
↑ Preantral /antral follicles	✔			✔	✖	✔	✔	✖	✖
Antral follicle arrest				✔	✖	✔		✖	
↑ Follicle atresia						✔	✔	✔	✔
↓ Granulosa and ↑ theca cell layer thickness								✔	
Altered follicular steroid hormone receptor expression				✔	✔				
Altered AMH or AMHR2 expression	✖			✔				✖	✖
↓ Oocyte maturation or developmental competence	✔	✖						✔	
↓ Fertility or fecundity	✔	✖		✔ (GD 60-90)				✔	✔
**2. Placenta and pregnancy**									
Pregnancy complications	✔							✔	✔
Placental defects				✔		✔		✔	✔
Fetal growth abnormalities, ↑AGD	✔	✖	✔	✔		✔	✔	✔	
↓ Placental capacity to metabolize androgens						✔		✔	✔
**3. Neuroendocrine regulation**									
↑ LH or ↑ LH:FSH ratio	✔	✖		✔			✔	✔**/**✖	✔
↑ GnRH/LH pulse frequency	✔			✔			✔	✔	✔
Altered hypothalamic GABA or KnDY neuronal morphology or function				✔			✔	✔	✔
↑ Pituitary LH responsiveness to GnRH	✔			✔	✔				
↓ E_2_ negative feedback	✔	✖		✔	✔				
↓ P_4_ negative feedback	✔			✔	✔			✔	
Compromised E_2_ positive feedback	✖	✖		✔	✖				
**4. Metabolic traits**									
↑ Body weight	✖	✖		✖		✔	✖	✖	✖
↑ Body fat or BMI	✔	✔		✖				✔**/**✖	
Adipocyte hypertrophy	✖			✖				✔	
Adipogenic constraint	✔			✔				✔	
Dyslipidemia	✔			✔				✖	
Insulin resistance	✔	✖		✔	✔	✔		✖	
Presence of liver steatosis				✔				✔**/**✖	
Pancreatic β-cell defects	✔	✖		✔				✔	
Hypertension/Echo-cardiographic alterations				✔		✔		✔	
**5. Behavioral traits**									
Behavioral deficits/abnormalities	✔	✔	✔	✔		✔		✔	
**6. References**	(41-44)	(42-44)	(42-45)	(46-85)	(47, 57-59, 61, 62, 64)	(86-90)	(91, 92)	(93-105)	(106, 107)

Abbreviations: AMH; anti-Müllerian hormone; DHT, dihydrotestosterone; T, testosterone.

✔, PCOS-like trait present; ✖, PCOS-like trait not present; “blank,” not reported by investigators in publication.

**Table 2.** Neonatal, Peripubertal, and Adult Manipulations Generating PCOS-like Features

Species	NHP			Rat				Mouse		
Time window of treatment	**Neonatal**	**Neonatal**	**Peripubertal**	**Peripubertal**	**Peripubertal**	**Peripubertal**	**Adult**	**Peripubertal**	**Peripubertal**	**Adult**
Treatment	**T**	**T**	**T**	**DHT**	**Letrozole**	**DHT**	**DHT**	**DHEA**	**Letrozole**	**Letrozole**
Age of treatment	1 day	1-51 days	1 years +	21 days	21 days	21-28 days	2 months	3 weeks	3 weeks	8 weeks
Dose of treatment	35 mg/kg (sc)	25 mg (sc) or 3-10 mg/kg/day	T capsules (sc) generating ~ 1.4 ng/ml circulating T levels	83 μg/day (sc)	200 µg/day (sc)	10 mg implant (sc) or 27.5 μg/day	4 mm (sc)	7.5 mg pellet (sc)	3 mg pellet (sc), 50 μg/day	3 mg pellet (sc), 50 μg/day
Duration of treatment	1 day	50 days	4-5 years	3 months	3 months	3 months	Replaced every month	3 months	5 weeks	5 weeks
**Traits approximating or biomarkers for criteria used for PCOS diagnosis in women (at least 2 out of 3 required)**										
Intermittent or absent ovulatory cycles	✖	✖	✖	✔	✔	✔	✔	✖	✔	✔
Elevated endogenous androgen levels or biomarkers of elevated androgens				♦	✖	♦	♦		✔	✔
Polyfollicular ovaries			✔	✔	✔	✔	✔		✔	✔
**Traits accompanying PCOS**										
**1. Ovary**										
↑ Ovary weight or size	✖		✖	✖	✔	✖	✖	✖	✔	✖
↑ Preantral /antral follicles				✔	✔	✖	✔	✖	✔	
Antral follicle arrest				✔	✔	✔	✔	✖	✔	
↑ Follicle atresia				✔	✔	✔		✖	✔	
↓ Granulosa cell layer thickness				✔	✔	✔				
↑ Theca cell layer thickness				✖	✔	✔				
Altered follicular steroid hormone receptor expression					✔	✔				
Altered AMH or AMHR2 expression			✖		✔		✔			
↓ Oocyte maturation or developmental competence			✔			✔	✔			
↓ Fertility or fecundity		✖	✔				✔		✔	
**2. Neuroendocrine regulation**										
↑ LH or ↑ LH:FSH ratio			✔		✔	✖	✖		✔	✔
↑ GnRH/LH pulse frequency			✔						✔	
↑ Pituitary LH responsiveness to GnRH			✖				✖			
↓ E_2_ negative feedback					✔					
↓ P_4_ serum levels				✔	✔	✔				
**3. Metabolic traits**										
↑ Body weight		✖	✖	✔	✔	✔	✖	✖	✔	✖
↑ Body fat or BMI			✖	✔	✔	✔	✖	✖	✔	✖
Adipocyte hypertrophy			✖	✔	✔	✔		✖	✔	
Adipogenic constraint			✖							
Dyslipidemia			✖	✖	✖	✔			✔	
Insulin resistance			✖	✔	✔	✔	✔	✖	✔	✖
Glucose intolerance			✖		✔	✔	✔	✖	✔	✖
Presence of steatosis						✔		✖	✖	
Pancreatic β-cell defects			✖			✔	✔		✔	
Hypertension						✔		✔		
Left ventricular fraction shortening						✔				
**4. Behavioral traits**										
Behavioral deficits /abnormalities		✔	✔							
**5. References**	(108)	(109)	(110-113)	(114-118)	(119-121)	(96, 102, 122-125)	(126-130)	(96, 131)	(132-135)	(136)

Abbreviations: DHEA, dehydroepiandrosterone; DHT, dihydrotestosterone; NHP, nonhuman primates; T, testosterone.

✔, PCOS-like trait present; ✖, PCOS-like trait not present; “blank,” not reported by investigators in publication, ♦, PCOS-like features could not be assessed as this trait is a feature of the hormonal modification.

**Table 3.** Genetically Manipulated Rodent Models

Species		Mouse		Rat
Modified gene	NGF/17NF overexpression	hCGb subunit overexpression	IR/LepR^POMC^ knockout	JCR:LA-*cp* LepR defect
**Traits approximating or biomarkers for criteria used for PCOS diagnosis in women (at least 2 out of 3 required)**				
Intermittent or absent ovulatory cycles	✔	✔	✔	✔
Elevated endogenous androgen levels or biomarkers of elevated androgens	✔	✔	✔	✔
Polyfollicular ovaries	✔	✔		✔
**Traits accompanying PCOS**				
**1. Ovary**				
↑ Ovary weight or size	✖			✖
↑ Preantral /antral follicles	✔	✖	✖	
Antral follicle arrest	✔	✔	✔	
↑ Follicle atresia	✔		✔	✔
↑ Theca cell layer thickness		✔		✖
↓ Oocyte maturation or developmental competence	✔		✔	
↓ Fertility or fecundity	✔	✔	✔	
**2. Neuroendocrine regulation**				
↑ LH or ↑ LH:FSH ratio	✖	✖	✔	
**3. Metabolic traits**				
↑ Body weight	✔	✔	✔	✔
↑ Body fat or BMI	✔	✔	✖	✔
Adipocyte hypertrophy			✔	
Dyslipidemia				✔
Insulin resistance/ hyperinsulinemia	✔		✔	✔
Glucose intolerance	✔		✔	✔
**4. References**	(137, 138)	(139)	(140, 141)	(142)

Abbreviations: hCGb, human chorionic gonadotropin subunit β; IR/LepR^POMC^ knockout, mice lacking leptin and insulin receptors in pro-opiomelanocortin neurons; LepR, leptin receptor; NGF, nerve growth factor.

✔, PCOS-like trait present; ✖, PCOS-like trait not present; “blank,” not reported by investigators in publication.

The current lack of clarity in the literature regarding when an animal model does or does not qualify as PCOS-like, is highly pertinent, because it causes confusion regarding the relevance or translatability of an animal model claiming to be PCOS-like (143). In addition, the recent proliferation of animal model publications requires context and perspective as to the specific usefulness and application of each model to PCOS, ranging across genetically derived mice models (31), hormonally and nonhormonally manipulated models (34, 38) and naturally hyperandrogenic NHP models with striking genetic similarity to humans (37, 144). Further elucidation of the underlying etiologies of PCOS is imperative if we are to develop new effective strategies to manage and potentially cure PCOS. Thus, we caution against recent clinical arguments to restrict PCOS research to humans because of the presumption that it is a “uniquely human disorder” (143). Such research restrictions will hinder not only progress in fundamental understanding of PCOS pathogenesis, but will also impair our appreciation of the ancient biological origins of PCOS (23, 25) that may prove vital in formulating transformative benefits to clinical care.

Consequently, this review aims to describe models that best reflect (Tables 1-3) or poorly reflect (Supplementary Table 1a and 1b) (36) clinical PCOS. The relevance of different NHP, sheep, and rodent PCOS-like models to specific aspects of PCOS etiopathogenesis and adult pathologic dysfunction will be discussed and will include consideration of accompanying neuroendocrine and metabolic dysfunction, together with other clinically relevant sequelae, based on a defined and targeted consideration of the literature in light of recent findings (Tables 1-3).

## Human PCOS—Hallmark Manifestations

### Defining diagnostic criteria for PCOS

Hyperandrogenism and hyperandrogenemia together form a key PCOS diagnostic feature exhibited by > 80% of women with PCOS. Clinical hyperandrogenism is defined by a modified Ferriman Gallwey score (mFG) ≥ 4 to 6, indicating hirsutism (145). Biochemical hyperandrogenemia in women with PCOS includes elevated circulating levels of T, as well as calculated bioavailable free (unbound) T and free androgen index (FAI), elevated circulating levels of androstenedione and elevated dehydroepiandrosterone sulfate (DHEA-S). Insufficient precision, sensitivity and specificity of methods used to measure circulating T, androstenedione and DHEA-S, and also estrogens, by liquid chromatography–tandem mass spectrometry versus assays based on antibody crossreactivity, make comparisons difficult to interpret between various studies (clinical or animal models), particularly since there are no trustworthy cutoff levels for biochemical hyperandrogenemia.

Polycystic ovarian morphology (PCOM) is defined as the presence of more than 20 follicles measuring 2 to 9 mm in diameter per human ovary and/or an increased ovarian volume of ≥ 10 cm^3^ (145). With PCOM, the number of follicles 2 to 5 mm in diameter positively correlates with serum androgen levels, while the number of follicles 6 to 9 mm in diameter negatively correlates with fasting serum insulin and testosterone levels, as well as body mass index (BMI), suggesting that ovarian hyperandrogenism promotes excessive early follicular growth that does not progress to the dominant stage due to hyperinsulinemia and/or androgen excess (3). These ovarian characteristics distinguish PCOM from other forms of polyfollicular ovarian morphology, which can be a normal stage of development in adolescence or can accompany other forms of ovarian dysfunction. Large, cystic ovarian follicles are therefore not typical of PCOM. PCOM is frequently observed in normal women, and several factors such as pharmacological treatment, may affect ovarian size and morphology (146, 147). Isolated PCOM without other diagnostic criteria is therefore not indicative of PCOS. Based on general population data, periods of irregular cycles in women with PCOS, defined as > 35 or < 21 days that persist 2 or more years postmenarche, are likely to indicate oligo-anovulation (145). With increasing adolescent gynecologic age, therefore, fewer pubertal women experience cycles exceeding 45 days (145), while adult women over 40 and with PCOS can exhibit more frequent ovulatory menstrual cycles (145).

### Hyperandrogenism, ovarian morphology, and follicular and oocyte dysfunction

**Figure 1.**
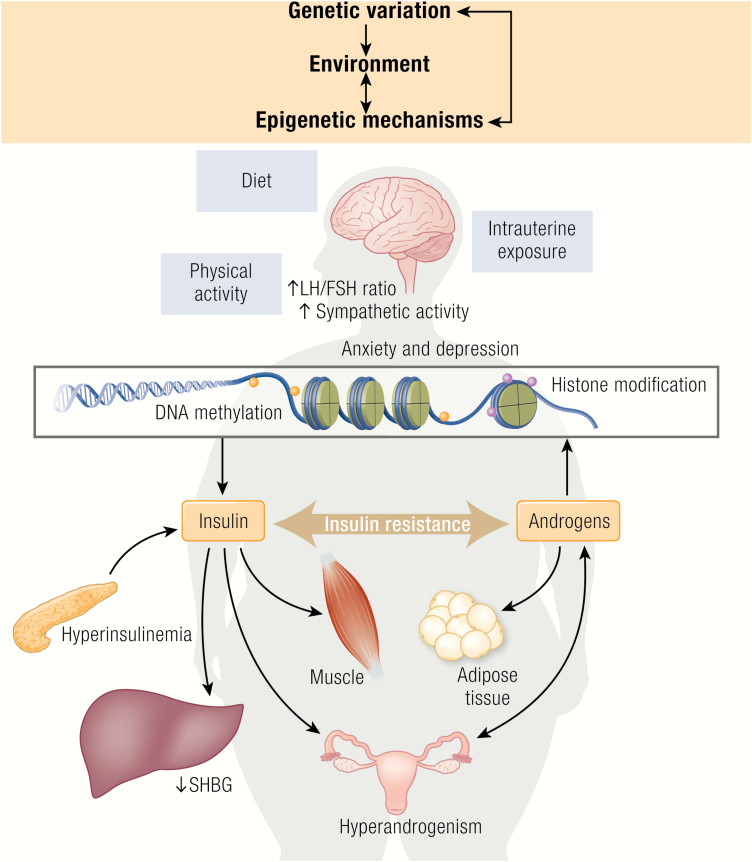
Hypothetical contribution of environmental, epigenetic, and genetic factors in the pathophysiology of PCOS. PCOS is a heterogeneous endocrine disorder and its pathogenesis is poorly understood. Current evidence supports gene-environment interactions and epigenetic regulation in the origins of PCOS. The inherited genetic component appears to span all organ systems and physiological function, with epigenetics capable of modifying the expression patterns of inherited genes. Environmental elements can influence all developmental components by impacting organ systems, physiological function and epigenetic regulation, with population-specific environmental elements thought to bring about ethnic differences in PCOS sub-phenotypes. While substantial gaps in knowledge still exist, further insights into our understanding of genetic and developmental contributions to the etiology of PCOS will significantly improve our ability to diagnose, treat, and prevent PCOS in the future.

**Figure 2.**
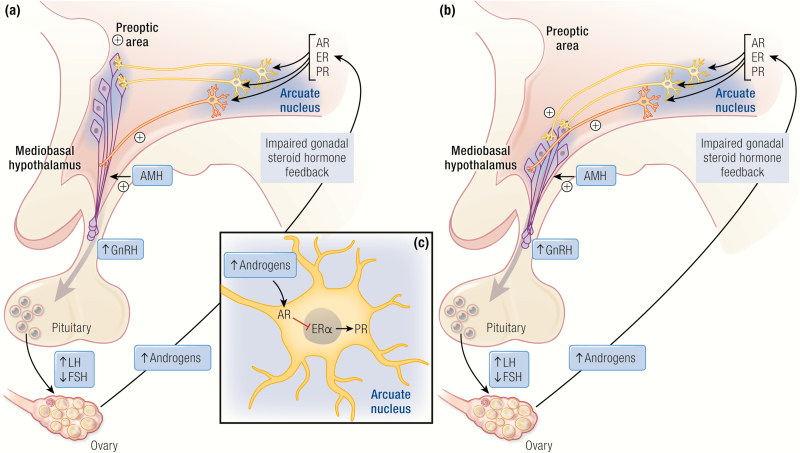
Neuroendocrine mechanisms potentially involved in mediating the development of PCOS. Substantial evidence now supports a key role for the neuroendocrine system in the pathogenesis of PCOS. Panel (A) illustrate rodent and panel (B) primate hypothalamus-pituitary-gonadal feed-back loops. The elevated LH:FSH ratio observed in women with PCOS is likely due to increased activity and secretion from GnRH neurons in the rostral forebrain (mice) and mediobasal hypothalamus (NHP and humans). The resulting increase in LH pulsatility contributes to the development of PCOS ovarian features, including theca cell hyperplasia and elevated androgen production. Impaired gonadal steroid hormone negative feedback sensitivity is indicative of brain specific impairments in the regulation of GnRH neurons. Recent research using animal models has identified that alterations in GABA, KNDy, and AMH brain-specific signaling are likely involved in GnRH neuron hyperactivity in PCOS. In hyperandrogenized PCOS animal models GABA circuit abnormalities develop before PCOS symptoms emerge (97, 158), and adult females exhibit increased GABA transmission to GnRH neurons (93), together with increased innervation to GnRH neurons (95), and yet AR blockade restores normal GABA innervation and transmission (93, 97). KNDy neurons play a key role in pulse generation (159, 160), however, varied subtle and frequently absent differences in the KNDy circuit have been identified in hyperandrogenized PCOS animal models (52, 53, 56, 91, 132, 161, 162). AMH directly stimulates GnRH activity (163) and prenatal maternal AMH excess has been shown to induce PCOS characteristics in mice, including increased GABAergic appositions to GnRH neurons and increased GnRH neuron firing rate in adulthood (107).

LH-regulated ovarian androgen production is the main source of androgen hypersecretion in women with PCOS, although adrenocorticotropic hormone (ACTH)-regulated adrenal androgen excess may contribute in approximately 25% who demonstrate enhanced 17-ketosteroid responses to ACTH (148-150). Intrinsic dysfunction within PCOS theca cells also contributes to ovarian hyperandrogenism (151, 152). Exaggerated ovarian antral follicle numbers observed in women with PCOS partly arise from theca cell hyperandrogenism, which promotes primary follicle recruitment, leading to increased numbers of gonadotropin-independent preantral and small antral follicles (153). LH hypersecretion in ~75% of women with PCOS (154) further stimulates theca cell hyperandrogenism, whereas relatively diminished follicle-stimulating hormone (FSH) levels inhibit expansion of follicular size and maturity, curtailing selection of a dominant, pre-ovulatory follicle and diminishing the likelihood of ovulation, as mechanistically illustrated in Figs. 1 and Figs. 2a and 2b. Importantly, normoandrogenic women with PCOM show increased androgen release to gonadotropin-releasing hormone (GnRH) agonist challenge and thus can also exhibit a hyperandrogenic ovarian response (155-157), but are not PCOS.

Hyperinsulinemia from insulin resistance related to PCOS can accompany hyperandrogenism and implicates metabolic dysfunction in women with PCOS. Insulin synergizes with LH to enhance ovarian theca cell androgen production, while also reducing hepatic sex hormone–binding globulin (SHBG) synthesis, causing elevated circulating levels of total and free T (164, 165). Furthermore, insulin excess inhibits ovarian follicular maturation through premature luteinization, as evidenced by augmented gonadotropin-stimulated estradiol and progesterone release (153, 166). This is because hyperinsulinemia enhances FSH-induced upregulation of LH receptors in granulosa cells during differentiation, which arrests cell proliferation and subsequent follicle growth, while increasing granulosa cell ability to produce progesterone in response to LH (167). Consequently, cultured granulosa cells from small PCOS follicles show a premature response to LH and an exaggerated steroidogenic shift from estradiol to progesterone production (168).

Hyperinsulinemia from adiposity-dependent insulin resistance can further sensitize thecal cells to LH stimulation and, in doing so, disrupt follicle development through synergistic actions of insulin and LH on enhancing ovarian theca cell androgen production (Fig. 1) (169-171). These insulin-LH interactions coexist in the presence of adipose-derived leptin overproduction that can inhibit FSH- and/or insulin-like growth factor 1 (IGF1)-stimulated granulosa cell steroidogenesis (172, 173).

In addition to ovarian hyperandrogenism, women with PCOS have 2- to 3-times higher circulating levels of AMH than women with healthy ovaries (174-176). AMH is a complex regulator of folliculogenesis and has been implicated in diminishing both primordial follicle recruitment (177-182) and FSH-regulated antral follicle development. AMH and AMH type 2 receptor, however, are not expressed in primordial follicles from NHP (183) and women (184). Elevated circulating AMH levels in women with PCOS are mainly due to large numbers of secondary preantral and nondominant antral follicles. Within women with PCOS, circulating AMH levels reflect the severity of PCOS phenotype, being higher in anovulatory than in ovulatory PCOS patients (185, 186). Measures of circulating AMH have been suggested to replace more costly and less accessible vaginal ultrasound in PCOS diagnosis, but a recent systematic review calls for more reliable cutoff values for AMH, assay standardization, and improved assay quality (176). Of interest, AMH diminishes primordial follicle recruitment in rodents, while it enhances primary to secondary follicle recruitment in NHP (177-182) before diminishing FSH-dependent antral follicle development. This emphasizes the importance of animal models in exploring AMH action during folliculogenesis across species and their differing implications for understanding ovarian folliculogenesis in women.

Intraovarian factors such as transforming growth factor-β (TGF-β) superfamily members beyond AMH, including inhibin, activin, bone morphogenic proteins (BMPs) and growth differentiation factors (GDFs) may also contribute to abnormal follicle development in women with PCOS (187). New generation serum assays suggest BMP15 and GDF9 associations are aberrant in women with PCOS (188). Furthermore, there are intriguing interactions between oocyte and granulosa cell signaling systems producing many of these intraovarian factors (189).

Single-cell transcriptomics have revealed delayed oocyte development in women with PCOS, likely due to diminished expression of genes associated with meiosis, gap junctional communication, hormone receptor signaling, and secreted factors (190, 191). This was further supported by increased expression of genes linked to DNA repair which may reflect low quality of oocytes and a poor environment for oogenesis. Furthermore, hormone receptors are downregulated in metaphase II (MII) stage oocytes, including AMH type 2 receptor (AMHR2), luteinizing hormone/chorionic gonadotropin receptor (LHCGR), oxytocin receptor (*OXTR*) and K (lysine) acetyltransferase 2B (*KAT2B*) (190). An additional study of MII-stage oocytes from women with PCOS found a preponderance of upregulated genes with particular relevance to meiosis regulation, including spindle dynamics, homologous recombination/chromosome alignment, cell cycle checkpoints, and centrosome function (192). Altogether, women with PCOS have dysfunctional ovarian follicular development and hyperandrogenic steroidogenesis, together with diminished oocyte quality, all of which require further investigation since currently employed therapeutic approaches ameliorate, but do not eliminate, follicle and oocyte impairments. Because our knowledge of oocyte quality in PCOS is based upon indirect markers of oocyte development, including gene expression and follicular fluid hormone levels (both *in vivo* and *in vitro*), animal models and *in vitro* follicle cultures are required to further understand the adverse implications of PCOS-related metabolic versus reproductive dysfunction on oocyte physiology.

### Hypothalamus-pituitary-gonadal axis

Increased LH pulse frequency, an elevated serum LH/FSH ratio, and greater pituitary LH responsiveness to GnRH in women with PCOS (31) are all likely due to an increased frequency of pulsatile GnRH secretion from neurons in the hypothalamus into the anterior pituitary portal venous drainage (3, 31, 193). The pulsatile secretory pattern for GnRH is regulated by feedback from the ovarian steroid hormones estradiol and progesterone (Figs. 2a and 2b). In contrast to women without PCOS, women with PCOS have an impaired negative feedback system, as evidenced by the need for higher doses of exogenously administered estradiol and progesterone to diminish elevated LH pulse frequency (194, 195). Since treatment of PCOS patients with the anti-androgen flutamide normalizes their estradiol and progesterone feedback regulation, elevated T may well act to diminish estradiol-mediated progesterone receptor expression within the hypothalamus of women with PCOS (Fig. 2b) (Marshall hypothesis) (196).

A recently noted AMH action at the hypothalamic level may additionally enhance GnRH release in women with PCOS (106). AMH receptors are expressed in human GnRH neurons and AMH can directly increase GnRH-dependent LH secretion (Fig. 2a, b) (163). These findings imply that high ovarian AMH levels in women with PCOS can regulate both ovarian follicle development and hypothalamic GnRH release (191). The brain represents another source of AMH production in rodents and humans, with GnRH neurons expressing both AMH and AMHR2 from early embryonic development to adulthood (197). It would be straightforward to measure AMH levels in the cerebrospinal fluid of women with and without PCOS and to use tissue-specific conditional AMH-knockout animals to examine ovarian versus neural AMH action on GnRH secretion (197).

### Obesity and metabolic disease accompanying PCOS

Metabolic disease-related sequelae for PCOS include insulin resistance and T2D, cardiovascular disease, dyslipidemia, abdominal obesity together with altered adipose tissue morphology and function, as well as sleep apnea (1-3). These additional traits all add considerable clinical complexity to the management of PCOS. Obesity is well known to enhance the severity of the PCOS phenotype (26), highlighted by the finding that for each 1-point increment in BMI above the normal range, PCOS prevalence increases ~9%, resulting in an increase of PCOS prevalence from ~5% at normal BMI to ~15% in obese women (26). This association does not necessarily suggest causation. Obesity and hyperandrogenism may independently affect female reproductive function. Considering that 30% to 50% of PCOS patients are normal weight, distinct molecular targets may underlie the pathogenesis associated with lean and obese PCOS. Therefore, it is important to investigate PCOS-like models that reflect a range of metabolic phenotypes. Due to the complexity of the PCOS phenotype, understanding the major pathological mechanisms underlying PCOS pathogenesis is problematic to investigate in humans. Animal models, therefore, provide a versatile platform from which to assess how different factors, including obesity, contribute and interact to mediate the pathogenesis of PCOS and accompanying sequelae, allowing insights into key pathological mechanisms that will enable development of new treatments.

### Familial and highly heritable

In a monozygotic twin study, the heritability of PCOS has been estimated as ~70% (18) which is almost double that in dizygotic twins ~40% (18) and suggests a genetic susceptibility to the disorder. A large-scale, genome-wide meta-analysis of PCOS found comparable genetic characteristics between self-reported PCOS and PCOS diagnosed by National Institutes of Health (NIH) or Rotterdam criteria (198), implicating shared genetic origins for the heterogenous phenotypes. At least 26 replicated PCOS risk genes have emerged from studies of human populations (6, 198-202) regulating a variety of reproductive functions, including gonadotropin secretion *(FSHB),* gonadotropin action and ovarian function *(AMH and AMHR2; LHCGR, STON1 and GTF2A1L; FSHR; DENND1A; RAB5B and SUOX: HMGA2; C9orf3; YAP1; TOX3; RAD50; FBN3)*, as well as metabolic *(THADA, GATA4 and NEIL2, ERBB2, ERBB3, ERBB4, SUMO1P1, INSR, KRR1)* and neural *(KCNA4)* function (6, 198, 199, 202). PCOS risk genes are associated with hyperandrogenism, including T levels, along with abnormal gonadotropin regulation. Furthermore, following Mendelian randomization analyses, BMI, fasting insulin, age at menopause, and depression, together with male-pattern balding among close male relatives, are all implicated in PCOS etiopathogenesis, thus providing a causal link to depression among women with PCOS and the first genetic evidence for a “male phenotype” for PCOS. Despite this, susceptible loci identified by genome-wide association study (GWAS) have only a modest effect size and explain only a minor portion of the estimated heritability. One possible explanation is that common genetic variants detected by GWAS have small biological effects, whereas rare genetic variants identified with whole-genome sequencing (WGS) or whole-exome sequencing (WES), likely have larger biological effects in complex diseases such as PCOS (203). This hypothesis has been tested by using WGS together with targeted sequencing in which 18 PCOS-specific rare *AMH* variants were identified (6), as well as 32 rare *DENNDA1A* variants among 50% (32 out of 62) of PCOS families (5). These findings implicate both AMH and DENNDA1A in the pathogenesis of PCOS. Interestingly, the posttranscription truncated isoform of *DENND1A* (DENND1A.V2) is overexpressed in women with PCOS and is functionally implicated in ovarian theca cell hyperandrogenism (204).While there are currently only a few studies investigating the contribution of rare genetic variants, this field will undoubtedly evolve in the future.

### Increasing evidence for developmental origins

Despite the progressive accumulation of evidence for PCOS risk genes (205), other factors increasing susceptibility to this complex disorder are likely involved, including environmental and epigenetic mechanisms. The developmental origin of adult disease (DoHAD) (206) or Barker hypothesis (207) refers to critical exposure(s) during gestation that permanently alter fetal physiology and/or morphology and fetal development, increasing the susceptibility to disease when adult, and likely influence phenotypic expression and transgenerational transmission of PCOS.

Circulating levels of T and other androgens are 3-fold higher in women with PCOS (208, 209), and the severity of reproductive and metabolic dysfunction, including pregnancy complications, are positively associated with maternal androgen levels (210-213). During pregnancy, such high levels of circulating androgens negatively affect placenta steroidogenesis and nutrient transport (214-216). In addition, women with PCOS are more often obese than women without PCOS, thus further increasing the risk of pregnancy complications, including miscarriage and gestational diabetes, as well as small or large for gestational age infants (210, 211). As discussed above, daughters of women with PCOS display 2 strong markers for *in utero* androgen exposure, a longer AGD and increased facial sebum production (19, 20). These clinical observations support the DoHAD hypothesis (206, 207) for fetal androgen excess contributing to developmental programming of PCOS. A population-based study has utilized maternal PCOS as a model of prenatal androgen exposure and demonstrates that daughters of women with PCOS are at increased risk of neuropsychiatric disorders, even when accounting for familial confounding, ie, genetic factors (217). This Swedish nationwide registry-based study with over ~29 700 daughters, of whom ~2300 were born to mothers diagnosed with PCOS, demonstrates that daughters of women with PCOS have a 5-fold increased risk of subsequent diagnosis with PCOS (10). These findings were further confirmed in daughters of women with PCOS from a separate case-control study in Chile. These PCOS daughters showed elevated circulating androgens, irregular menstrual cycles, and polycystic ovaries, as well as metabolic disturbances including elevated blood pressure, larger waist circumference indicating abdominal obesity, and higher BMI than daughters of women without PCOS. Of note, these findings cannot separate eventual confounding genetic factors from a causal association. Investigation into whether elevated maternal androgen influences transgenerational susceptibility to PCOS is not yet feasible in humans, and longitudinal studies following women with PCOS across several generations are logistically unrealistic. Therefore, PCOS animal models provide an opportunity to greatly increase our knowledge of how PCOS phenotypic expression is programmed by an altered maternal endocrine-metabolic environment, potentially through developmental epigenetic modifications that adversely affect long-term offspring health (organizational effects).

## Animal Models of PCOS

Evolutionarily conserved mammalian physiological systems enable the use of experimentally manipulated or naturally occurring animal models to provide biological and clinically relevant insight into PCOS etiopathogenesis. Animal models allow highly invasive investigative procedures that are otherwise unethical in humans. Indeed, fundamental understanding of a human disorder is often only identified following insightful revelations from customized animal models. For example, estrogen resistance was considered incompatible with life until the first estrogen receptor knockout mouse was reported (218), a finding subsequently confirmed in humans. Further elucidation of PCOS etiopathogenesis utilizing animal models is imperative if we are to develop more effective strategies to manage and potentially cure PCOS.

### What is a relevant animal PCOS model?

Animal models of relevance to PCOS must, by necessity, have comparability to women with PCOS by exhibiting 2 or more PCOS-like equivalents of the Rotterdam criteria, as illustrated in Tables 1-3 and Supplementary Table 2 (36). Such models stand in contrast to those illustrated in Supplementary Tables 1a and 1b (36) exhibiting **(i)** only a single PCOS-like trait, such as T-treated neonatal rats, **(ii)** 2 or more PCOS-like traits alongside PCOS endocrine-mimics (including hyperprolactinemia and hypogonadotropic amenorrhea), such as DHEA-treated peripubertal or adult female rodents, or **(iii)** 2 or more PCOS-like traits generated by non-PCOS like mechanisms, (such as testis Leydig cell-typical HSD17B3 contributing to ovarian theca cell hyperandrogenism) as found in estrogen receptor or aromatase knock-out female mice. In this review, a clear distinction is made between such animal models with potentially limited mechanistic relevance for PCOS, as illustrated in Supplementary Tables 1a and 1b (36), and those illustrated in Supplementary Table 2 (36), where genetically modified mice, that are themselves not PCOS-like models, have been combined with a rodent PCOS-like model and have clearly demonstrated their relevance towards PCOS mechanistic understanding. These latter animal models comprise peripubertal dihydrotestosterone (DHT)-induced PCOS-like mice combined with female mice genetically manipulated, including those with whole body or organ/cell specific gene knockout of androgen receptors (AR) (219). Such models are contributing immensely to our molecular understanding, as some are unresponsive to androgen programming of a PCOS-like adult phenotype, hence demonstrating the vital contribution of AR either during development and/or in a single organ system as the molecular foundation on which prenatal PCOS-like programming and the DoHAD hypothesis relevant to PCOS are built.

### Prenatal models

Prenatally androgenized (PNA) female NHP, sheep, rats, and mice manifest reproductive and metabolic PCOS-like phenotypes in adulthood, as described in Table 1. Collectively, these animals provide unique perspectives of how hyperandrogenism and obesity interact to worsen the PCOS phenotype, as seen in Western societies due to the obesity epidemic. Furthermore, normal-weight women with PCOS, defined by NIH criteria, may or may not exhibit metabolic dysfunction (220-222). PNA models are derived from injecting their dams subcutaneously or intramuscularly with T, T propionate, DHT, DHT propionate, or AMH at various doses and at various gestational ages ranging from early-to-mid to late gestation. The doses used for NHP exceed those for nonprimates in order to overwhelm the NHP placenta’s extensive capacity to aromatize or inactivate androgen (41). NHP models provide the most comprehensive obese PCOS-like phenotypes, particularly early- to mid-gestation PNA NHP. Their genomic and epigenomic comparability to humans enables ready translation of understanding and therapeutic modeling. PNA sheep, which also represent a lean PCOS-like phenotype, have enabled multiple longitudinal studies aimed at careful characterization of PCOS-like traits at multiple developmental time points (223), identifying pre-PCOS-like traits and biomarkers of high translational interest to pediatricians. PNA female NHP and sheep also provide attractive model attributes, including precocial offspring, absence of litters (typical of rodents, but not humans), use of various surgical and experimental procedures and interventions, and relatively large body sizes for detailed and repetitive hormonal profiling and measurement of hypothalamic neuropeptides. Developmental trajectories of several organ systems in NHP and sheep also emulate their counterparts in humans, therefore providing strong translational relevance. In one sense, as habitual mono-ovulators, NHP and sheep models can truly emulate polycystic ovaries, in contrast to multi-ovular rodents, providing more directly translatable understanding regarding ovarian pathophysiology.

Several PNA mice models represent a lean PCOS-like phenotype by recapitulating the reproductive and neuroendocrine pathology of PCOS. Perhaps the most striking of all PNA mouse attributes, however, is the ability to commit genetically manipulated female mice to PNA programming to identify the molecular profile of PNA, and then develop potential therapeutic countermeasures. The PNA mouse model using DHT administration in late gestation enables elucidation of AR-mediated mechanisms involved in PCOS etiopathogenesis, avoiding the confounding effects of T aromatization to estradiol. PNA adult mice exhibit elevated plasma levels of T (hyperandrogenism), impaired estrous cyclicity (oligoovulation), and modified follicular wall morphology similar to PCOS, specifically decreased granulosa cell layer and increased theca cell layer thickness (93-96). PNA treatment of AR knockout (ARKO) mice does not cause hyperandrogenism, disrupted estrous cyclicity, or altered ovarian morphology, suggesting that the PNA-induced, PCOS-like mouse phenotype is dependent upon AR signaling (161).

Circulating AMH levels are significantly higher in naturally occurring hyperandrogenic female rhesus monkeys (Supplementary Table 3) (36), which mimics cardinal features of women with PCOS (37). Interestingly, 2 recent studies (107, 224) showed that pregnant women with PCOS maintain significantly higher serum AMH levels and a positive correlation exists between gestational AMH and androgen levels in humans during late pregnancy (107). Interestingly, this corresponds with a time window sensitive to triggering the PCOS-like phenotype in offspring of PNA models (224). This 2-fold increase in AMH levels compared with controls implicates AMH as another potential candidate in the prenatal androgen excess programming of PCOS. In support of this hypothesis, excess prenatal AMH exposure in mice engages a series of events in the dams, which leads to a fetal androgen excess programming of exposed female offspring into a PCOS-like reproductive and neuroendocrine phenotype in adulthood (Table 1) (107).

### Neonatal, peripubertal, and adult models

Neonatal T and DHT treatments of female mice and rats, and neonatal and adult T treatments of NHP, all fail to induce sufficiently PCOS-like animal models (Supplementary Table 1) (36). In contrast, peripubertal DHT exposure of mice and rats by subcutaneous implantation of ~3- to 4-week-old females with an implant containing DHT for a period of 3 months (96, 114, 115, 122, 123, 225-227) elicits an adult phenotype that displays a breadth of endocrine, reproductive, and metabolic PCOS-like traits (Table 2). Continuous low-dose DHT exposure initiated in adulthood can also initiate PCOS-like reproductive dysfunction and some metabolic features, including insulin resistance and pancreatic B-cell defects (126-129).

The peripubertal letrozole-induced PCOS-like mouse and rat models are generated in an analogous way to the DHT-induced model. Letrozole is an aromatase inhibitor that causes endogenous hyperandrogenaemia, neuroendocrine alterations, and reproductive as well as metabolic abnormalities (115, 119, 120, 123, 132, 228) (Table 2 and Supplementary Table 1a and 1b) (36). Interestingly, adding anti-TNFα, anti-inflammatory therapy to continuing letrozole treatment largely reversed hyperandrogenemia as well as reproductive and metabolic PCOS-like traits (121). This model is based on the finding in hyperandrogenic PCOS women of rare genetic variants of *CYP19A1*, the aromatase gene converting androgens to estrogen, that are associated with lower aromatase activity (229-231). However, women with NIH-defined PCOS (but perhaps not all) are estrogen-replete, having circulating estrogen levels comparable to the midfollicular phase of the normal menstrual cycle (232, 233). Administration of letrozole to adult female mice results in a similar reproductive phenotype, but unlike the peripubertal letrozole-induced mice, adult female mice exposed to continuous letrozole do not develop obesity or insulin resistance (136). Therefore, this model represents the lean reproductive phenotype.

Peripubertal T induced PCOS-like NHP exhibit polyfollicular ovaries and diminished fertility and fecundity in the presence of continuous exogenous T (Table 2). Accompanying PCOS-like traits include LH hypersecretion. When combined with diet-induced obesity (DIO), peripubertal T NHP exhibit metabolic dysfunction, including metabolic compromise of the placenta during gestation. Collectively, these models demonstrate the powerful activational action of postnatal manipulations that can generate sufficient PCOS-like traits from females in the absence of organizational effects on gestational programming. In contrast to models induced by organizational actions of manipulations during development (Table 1), there is little or no evidence for permanently reprogrammed PCOS-like phenotypes following activational postnatal manipulation. In this latter regard, ~83% of female-to-male adolescent and adult transgender patients resumed menstrual cycles with normal circulating levels of estradiol, FSH and AMH at ~4 months following cessation of approximately 3 to 4 years of transgender androgen therapy (234), suggesting that adolescent or adult onset T treatment does not reorganize ovarian function.

Naturally occurring female hyperandrogenism occurs in adult female macaques with PCOS-like reproductive and metabolic traits (37, 144) and potentially has mid-gestation hyperandrogenic origins. Rhesus macaques share > 95% DNA sequence identity with humans at protein-coding exons, confirming a close evolutionary history. Studies of complex polygenic diseases in these NHP demonstrate how damaging mutations often generate pathological phenotypes almost indistinguishable from analogous diseases in humans (235-241). Therefore, gene variants in the rhesus macaque exome that resemble those previously identified in human PCOS candidate genes are likely to have comparable physiological consequences across primates.

### Genetic models and combined PCOS-like and genetic models

The generation of customized genetically manipulated mouse models has provided a versatile and valuable tool for decisive mechanistic studies aimed at understanding the underlying pathogenic mechanisms involved in the development of PCOS. In recent studies, in which ARKO mouse models have been combined with DHT-induced mouse models of PCOS, significant advances have been made in deciphering the role of hyperandrogenism and its site of action in the organizational development of PCOS characteristics. Complete or partial AR insufficiency protects PNA female mice from developing characteristics of PCOS, including acyclicity, ovulatory dysfunction, and adipocyte hypertrophy (161). These results infer a key role for AR-mediated actions in the development of PCOS. Moreover, recent studies have used molecular modeling in global and cell-specific ARKO mouse models to begin deciphering key AR target sites involved in the pathogenesis of PCOS. Transgenic silencing of AR actions in the brain in peripubertal DHT-induced experimental PCOS mice either fully or partially prevent the development of most reproductive and metabolic PCOS traits normally observed, including ovulatory dysfunction, polyfollicular ovaries, adiposity, adipocyte hypertrophy, dyslipidemia, and hepatic steatosis (124). Additionally, a pituitary-specific loss of AR signaling protects against the development of cycle irregularity and ovulatory dysfunction (126) and when peripubertal DHT onset commences in ovariectomized global ARKO mice with transplanted control ovaries (ovaries have functioning AR), normal ovarian cycle patterns are maintained (124). In comparison, loss of AR function in granulosa cells alone did not protect mice from developing most PCOS characteristics (124). Similarly, inactivation of theca cell AR signaling in a hyperandrogenized PCOS mouse model only partially protected against the PCOS traits of acyclicity, ovulatory dysfunction, and infertility (242). Collectively, these findings emphasize the brain as a crucial site for androgen actions at the core of PCOS pathogenesis (219) (Supplementary Table 2) (36).

Adiponectin secreted from adipose tissue can be decreased in women with PCOS and low levels are strongly associated with insulin resistance (243). The adiponectin-overexpressing transgenic mouse has been used to study the causal relationship between adiponectin levels and reproductive as well as metabolic functions in the peripubertal DHT-induced PCOS mouse model (244). DHT exposure in wild-type mice decreases adiponectin levels, revealing that elevated adiponectin levels in these transgenic mice enables them to remain metabolically healthy despite DHT exposure. Reproductive function, however, is still impaired by peripubertal DHT exposure (Supplementary Table 2) (36).

Altered sympathetic activity has been proposed in the development of PCOS. Indirect and direct measurements of sympathetic nerve activity suggest sympathetic hyperactivation in PCOS. T levels are positively correlated with high sympathetic nerve activity in women with PCOS (245) and an increase in sympathetic outflow impairs metabolic and reproductive functions (245-249). Increased expression of nerve growth factor (NGF) has been found in ovaries from women with PCOS (137). Excess intraovarian NGF elevates sympathetic responses and may initiate ovarian pathology. Support for NGF overproduction as a contributor to pathological conditions of PCOS comes from transgenic mice that overexpress NGF in ovarian theca cells (17NF) and exhibit reproductive and metabolic characteristics of PCOS (137, 138) (Table 3).

Recent evidence points toward the brain as a key target site in the development of PCOS, with insights into potential underlying pathways involved coming from the examination of novel transgenic models. Specific deletion of insulin receptors (IR) and leptin receptors (LepR) from the pro-opiomelanocortin (POMC) neurons in a mouse model (IR/LepR^POMC^) results in a PCOS phenotype with female mice displaying irregular cycles, dysfunctional ovulation, reduced fertility, elevated circulating T and insulin levels, increased fat mass and adipocyte hypertrophy, as well as reduced glucose tolerance and insulin resistance (140, 141). In addition, the JCR:LA-*cp* rodent model, which displays a malfunction of the leptin receptor, has been put forward as a potential model to study the etiology of metabolic disturbances associated with PCOS as it displays increased T concentrations, oligo-ovulation, obesity, insulin resistance, and dyslipidemia (142).

For future investigations into the underlying pathways involved in driving PCOS, the use of transgenic models has the advantage of providing a platform to study specific candidate genes, such as those variants identified from genetic studies of women with PCOS, in isolation or in combinations. Assessment of whether changes in the function of PCOS candidate genes lead to the development of or protection from PCOS-like traits in animal models will provide key insights into PCOS etiology and hence are vital tools for improving our knowledge of the pathogenesis of PCOS.

## Reproduction

### Neuroendocrinology

#### PNA and prenatal AMH (PAMH) models.

Hypersecretion of pituitary LH, evident in > 75% of women with PCOS (154), is an almost universal consequence of PNA modeling, from mice to NHP, strongly implicating PNA programming of this neuroendocrine trait. Late gestation PNA in NHP and sheep, however, are the exception, likely due to late gestation PNA occurring after the crucial early- to mid-gestation developmental window for hypothalamic differentiation, following which fetal females no longer respond to reproductive neuroendocrine reprogramming (42). Mechanistic components of this developmental organization have been identified in adult PNA models from characterizing accelerated LH pulse dynamics and associated steroid hormone regulation, providing evidence for disruption of 3 hypothalamic-pituitary feedback systems regulating (GnRH) LH release, namely: negative feedback mediated by estradiol in PNA early- to mid-gestation NHP, sheep, rats, and mice (46, 47, 94, 250) and by progesterone in PNA NHP, sheep, and mice (48, 49, 95, 97, 251), together with positive feedback mediated by estradiol in PNA sheep and rat (50, 51, 252), but not in PNA NHP model or PNA mice (43, 94, 250), the latter emulating women with PCOS (253). Interestingly, since both PNA sheep and mice produced by DHT treatment (31) demonstrate normal positive feedback LH responses to estradiol (94), they provide a clear contrast to PNA sheep produced by T treatment that fail to show positive feedback LH responses to estradiol (47, 254). Programming of impaired positive feedback in nonprimate PNA models may therefore likely involve estrogen receptor (ER)-α–mediated mechanisms.

PNA sheep and PNA rodent models display changes in hypothalamic kisspeptin/neurokinin B/dynorphin (KNDy) expression and circuitry (52-55, 91, 132, 162), implicating the KNDy system as an attractive therapeutic target for modulating AR-driven, pathological neuroendocrine activity in women with PCOS. Indeed, increased circulating levels of kisspeptin have been reported in several populations of PCOS women (255-257), supporting the notion of specific alterations to hypothalamic circuits that may underlie disrupted neuroendocrine regulation of fertility in PCOS. Kisspeptin in the systemic circulation, however, is likely produced from the pituitary and the pancreas. Neuroanatomical evidence implicates kisspeptin/ neurokinin-B/dynorphin and gamma-aminobutyric acid (GABA)ergic neuronal populations in diminished progesterone negative feedback sensitivity on GnRH release (53), including increased neurokinin B receptor, GABAergic input and projections onto GnRH neurons (52, 54-56, 93, 95, 193, 258). At the level of the gonadotrope, pituitary responsiveness to GnRH is increased in early- to mid-gestation PNA NHP and sheep (42, 57) as in women with PCOS. Notably, in PNA sheep, these studies are undertaken after ablation of endogenous GnRH action. PNA-induced developmental changes in female sheep pituitary mRNA expression of GnRH receptor and estrogen receptor α (ESR1), regulators of pituitary sensitivity to both estradiol negative and positive feedback, are implicated in the differential control of LH and FSH (57), partially through hypothalamic AR-mediated inhibition of ESR1-mediated progesterone receptor (PR) expression as evidenced in rodents (259). PR expression is indeed markedly reduced throughout the hypothalamus of PNA female mice, including the anteroventral periventricular (AVPV) nucleus, and, most dramatically in the arcuate nucleus, while ESR1 expression is largely unchanged (95). Within the female arcuate nucleus, PR expression is particularly reduced within GABA neurons (95), suggesting a role for arcuate GABA neurons in mediating diminished progesterone negative feedback in PCOS women and early- to mid-gestation PNA NHP (251). It remains to be determined whether PR expression is diminished in KNDy neurons, key components of the GnRH pulse generator (260) however, the kisspeptin-GnRH/LH system, dynorphin mRNA expression, and kisspeptin cell number are largely unchanged within the arcuate nucleus of PNA mice (161, 261). Taken together, these results suggest that PNA-induced LH hypersecretion is likely the consequence of reduced hypothalamic sensitivity to steroid hormone negative feedback, potentially from diminished neuronal PR expression, together with dysregulated pituitary responsiveness to GnRH (Fig. 2a, b).

Mouse models of PCOS can employ transgenic approaches (262) to dissect the intricate neural regulation of GnRH release. Transgenic mice expressing green fluorescent protein (GFP), specifically in hypothalamic GnRH neurons (GnRH-GFP mice) (263, 264), have facilitated previously unachievable discoveries about the anatomical and functional changes in neuroendocrine circuitry associated with PCOS-like features. These novel mouse models have enabled the first identification of pronounced hypothalamic GnRH neuronal afferent remodeling and altered development and activity of GnRH neurons that accompany LH (and GnRH) hypersecretion. Studies of spontaneous GABAergic events in GnRH neurons found increased GABAergic post-synaptic currents in GnRH neurons of PNA mice (93, 158). They also exhibit anatomical evidence for elevated dendritic spine density and increased GABAergic afferent innervation to GnRH neurons (95, 97), originating largely from the arcuate nucleus (95), a hypothalamic region exquisitely involved in GnRH regulation in all female mammals (265, 266). While widely regarded as the primary inhibitory neurotransmitter in the adult brain, GABA transmission typically depolarizes GnRH neurons (265-267). Thus, increased GABAergic innervation and transmission to GnRH neurons in PNA females likely reflects greater potential to excite these neurons. This finding is quite contrary to prevailing understanding of GABAergic input as hyperpolarizing neurons and stands in marked contrast to the previously well-established inhibitory role of GABAergic-mediated inhibition of GnRH release responsible for the prepubertal “brake” on female reproductive maturation in female NHP (159). Excitatory GABAergic input is dependent upon chloride ion (Cl^-^) extrusion through the GABA_A_ receptor (266, 267). GnRH neurons in mice maintain a higher [Cl^-^]i in adulthood by maintaining expression of the Na^+^-K^+^-Cl^-^ cotransporter 1, NKCC1 (55, 265-269). PNA female sheep exhibit increased NKCC1 expression on preoptic area GnRH neurons (the most relevant population of GnRH neurons regulating female reproduction in nonprimates, as illustrated in Fig. 2), and increased NKCC1 expression on arcuate nucleus KNDy neurons intimately involved in regulating GnRH release (55).

In this regard, women receiving valproic acid, an anti-epileptic medication that increases central GABA levels, develop PCOS-like symptoms (270), and women with PCOS exhibit increased cerebrospinal fluid concentrations of GABA (271). These clinical correlates further support the notion that PNA programs LH hypersecretion in > 75% of women with PCOS by increasing GnRH neuron depolarization through increased GnRH and KNDy neuronal expression of GABA_A_ receptor–regulated chloride ion transporters. Such novel appreciation of GABA neuronal regulation of GnRH neurons reveals a potential neural therapeutic target for women with PCOS. Recently, acute activation of GABA neurons in the arcuate nucleus of the female mice hypothalamus with opto- or pharmaco-genetics was shown to elicit a long-lasting increase in LH secretion, while chronic activation was found to disrupt reproductive cycling and promote elevation of T levels, a functional induction of PCOS-like reproductive traits resembling those in PNA models (98).

Interestingly, postnatal anti-androgen treatment in PNA female mice normalizes neuroendocrine and ovulatory function (93, 97), while in PNA sheep, anti-androgen treatment normalizes timing of pubertal onset and restores preovulatory follicle growth (272) together with preovulatory LH surges (273). Gestational anti-androgen treatment, alone, restores LH surges, albeit of low amplitude (273). In some women with PCOS, 6 months of anti-androgen therapy also improves fertility, menstrual cyclicity, and LH levels (274), while 7 to 10 days of anti-androgen normalizes progesterone negative feedback regulation of episodic GnRH/LH release (194, 195). In agreement with these findings, normalization of LH pulsatility through intermittent administration of a GnRH antagonist has also been shown to normalize neuroendocrine and ovulatory function in adult PAMH mice (107).

These therapeutic findings strongly suggest that PCOS and PCOS-like traits require ongoing hyperandrogenic action mediated through AR at the hypothalamo-pituitary and/or ovarian levels to maintain PCOS pathophysiology. By no means does this eliminate important roles for ESR1 and PR in PCOS pathogenic mechanisms, but these findings together with those from the “PCOS rescue” ARKO models (Supplementary Table 2) (36), place ESR1 and PR downstream of AR in the pathogenic induction of PCOS-like traits. In line with this, DHT increases and progesterone decreases GABA-mediated post synaptic currents in GnRH neurons (275) and DHT interferes with progesterone inhibition of GABA activity (276). The specific mechanisms by which androgens impact ER/PR actions and expression are not clear, but likely involve androgen-mediated suppression of PR transcription. Acute T administration to female rats reduces hypothalamic PR mRNA and prevents an estradiol-induced rise in PR mRNA expression (252). DHT can also reduce progesterone-induced PR transcriptional activity in preovulatory gonadotropes in vitro (252, 277). Work done in breast cancer line lines, in which DHT downregulates PR expression, suggests that androgens interfere with ER complexes (278, 279). Another possibility includes epigenetic modification of the PR gene directly.

Gestational treatment with an insulin sensitizer does little to alter PCOS-like neuroendocrine and reproductive traits in PNA sheep (273), whereas postnatal treatment with an insulin sensitizer improves reproductive endocrine parameters and normalizes cycles in both female PNA NHP and sheep (280, 281). PNA NHP and humans, however, provide salient lessons against gestational intervention designed to counteract PCOS-like programming. In NHP, gestational anti-androgen therapy induces subtle cognitive dysfunction and behavioral changes in adult female offspring (282) and in humans, gestational metformin treatment of women with PCOS worsens metabolic dysfunction and weight gain in their prepubertal daughters (283). Such understanding counsels for development of novel therapies or interventions postpartum, to avoid the high risks of gestational manipulations.

Another prenatal exposure model that mirrors PCOS features was achieved by elevating dam AMH levels during late gestation (PAMH) which generates a 3-fold increase in maternal T levels (Table 1), thus closely mimicking the hyperandrogenic environment of human PCOS pregnancies (107). AMH-induced hyperandrogenism in both dams and their female fetuses is likely responsible for rewiring fetal female hypothalamic circuitry to enable excessive excitatory inputs onto GnRH neurons, leading to acquisition of hyperandrogenic PCOS-like traits in adult female offspring (Table 1). Consistent with a crucial role for PNA as a major driver of these PCOS-like traits, protracted changes in GnRH neuronal morphology (increased dendritic spine density) and increased excitatory (GABAergic) appositions onto GnRH neurons in adult PAMH offspring have been reported (107), closely mimicking the aberrant neurocircuitry of PNA mice (93, 95, 97) and further reinforcing the notion of hypothalamic GABAergic activation as a programmed driver of LH hypersecretion. Marked masculinization of the sexually dimorphic brain regions regulating reproduction are found in PAMH female offspring, including kisspeptin and tyrosine hydroxylase neurons in the AVPV, and vasopressin neurons in the bed nucleus of the stria terminalis and medial amygdala. Notably, GnRH neurons of PAMH mice have a robust 3-fold increase in their spontaneous action potential firing rate (neuronal hyperactivity), as compared with controls (107), increasing their resemblance of PNA mice (158). In addition, and closely mimicking women with PCOS, PAMH mice also exhibit a higher LH pulse frequency, reflecting their increased upstream hypothalamic GnRH neuronal firing rate. It remains to be determined, however, whether impairments are also present in the homeostatic feedback mechanism between the gonads and the central brain circuits regulating fertility in PAMH female mice, as found in PNA animal models and in women with PCOS. Interestingly, PCOS-like traits in PAMH female pups are prevented by concurrent GnRH antagonist administration to pregnant dams, indicating that PCOS-like traits in female offspring are programmed *in utero* by fetal and/or maternal androgen excess (107). The work of Tata and colleagues (107) thus raises the intriguing hypothesis as to whether the origin of gestational hyperandrogenism in women with PCOS resides with elevated maternal AMH levels during pregnancy, although a causal relationship between AMH and T during human gestation remains to be established.

#### Peripubertal and genetic models.

Female transgenic mice models with loss of AR signaling in the brain and pituitary (NeurARKO) exhibit aberrant neuroendocrine control with females displaying elevated LH levels at diestrus, a diminished serum LH response to ovariectomy and E_2_ priming, and reduced kisspeptin mRNA expression in the AVPV, but elevated kisspeptin and neurokinin B mRNA expression in the arcuate nucleus at proestrus (52-55, 91, 132, 162, 255-257, 284).

Hypothalamic neuroendocrine regulation of GnRH release in females, however, may be additionally susceptible to hyperandrogenic dysregulation at puberty. Peripubertal onset of T excess in female NHP induces LH hypersecretion in adulthood (110) and a transient, peripubertal acceleration in episodic LH release (111). It is unclear whether ovarian hyperandrogenism is induced by this LH excess since exogenous T treatment of the NHP is unremitting. Peripubertal DHT-exposure in mice certainly leads to increased pituitary gene expression of LH, GnRH receptors, and kisspeptin receptors (244). Despite these changes in pituitary gene expression, the circulating LH and LH/FSH ratio is unaltered in peripubertally DHT-exposed mice, suggesting that GnRH signaling is not increased in this model (96, 122, 244); however, LH pulse frequency remains to be determined. Peripubertal letrozole exposure increases circulating LH and LH pulse frequency and amplitude like in women with PCOS, together with increased *Lhb* expression, and decreases circulating FSH and *Fshb* expression in the pituitary (132, 133). Furthermore, peripubertal letrozole-exposure leads to upregulation of *Kiss1r* gene expression in the rostral preoptic area and *Pgr* expression is lower in the mediobasal hypothalamus region that includes the arcuate nucleus, and is associated with progesterone negative feedback in this model (132). In addition, elevated *Kiss1, Tac2* and *Pdyn* gene expression, and increased Kiss1 neuronal activation, in the hypothalamic arcuate nucleus have been observed (133).

More in-depth investigations of the hypothalamic circuitry implicated in controlling female reproduction, and its regulation by mechanisms governing energy homeostasis, are urgently needed to better understand how numerous neuroendocrine functions integrate metabolic feedback in PCOS-like animal models.

### Ovarian morphology, estrus cycle, and ovulatory function

Intermittent or absent ovulatory cycles are an almost universal PCOS-like trait, found in PNA, peripubertal and genetically manipulated animal models of PCOS (Tables 1-3). Notable exceptions are models of peripubertal onset DHEA excess in mice and T excess in NHP, adult-onset androgen excess in NHP and naturally occurring adult female hyperandrogenism among NHP. DHEA is a weak androgen and requires target organ enzymatic conversion to more potent androgens (285), while exhibiting specific action through a variety of nuclear and G-coupled receptors (286). Adult onset androgen excess and naturally occurring adult hyperandrogenism may reflect omission or insufficient androgen excess during relevant developmental “windows.” A predominance of intermittent or absent ovulatory cycles in PCOS-like females may thus require developmental reorganization, including structural and functional changes in hypothalamic neurocircuitry that can be therapeutically reversed in adulthood, as discussed in the neuroendocrine section above.

There are, however, 2 important caveats regarding developmental PNA programming of ovulatory function. First, in nonprimates, but not in primates, aromatizable androgen-mediated developmental reprogramming of regular ovulatory function involves eradication of hypothalamic neuroendocrine ability to generate an ovulation-inducing LH surge, whereas nonaromatizable androgen fails to do this (94, 254). In other words, PNA eradication of positive feedback in nonprimates requires the combined action of both AR and ER in the fetal female hypothalamus. Positive feedback in primates, including humans, is however unaffected by exposure to either androgen during gestation. Secondly, in NHP, unlike nonprimates, PNA and naturally hyperandrogenic PCOS-like models exhibit all 4 PCOS-like phenotypes, including ovulatory PCOS (type C). Thus, statistically significant expression of intermittent or absent ovulatory cycles, or its absence, in a PCOS-like model does not preclude a diversity of ovulatory function or lack thereof. Interestingly, PNA NHP models exhibit a majority of more severe, oligo-ovulatory PCOS-like phenotypes, emulating clinically referred women with PCOS, whereas, conversely, adult female NHP exhibiting natural hyperandrogenism manifest a majority of the milder, ovulatory PCOS-like phenotypes (37), emulating women with PCOS from unselected populations.

Large, polyfollicular ovaries are found in only early- to mid-gestation PNA NHP and sheep (Table 1), suggesting that developmental androgen-mediated reorganization is required for such morphologically-relevant ovarian enlargement and maybe unique to female hyperandrogenism in commonly mono-ovular species. In contrast, increased ovarian antral follicle count (AFC) or circulating AMH levels (as a biomarker for increased AFC), indicative of polyfollicular ovaries, but not necessarily PCOM or ovarian cysts, occurs in many PCOS-like models, including PNA NHP, sheep and rat, peripubertal onset DHT mice and T NHP, transgenic NGF/17NF mice and naturally hyperandrogenic NHP (Table 1). PNA, peripubertal, and subsequent adult female hyperandrogenism, however, may be sufficient for increased ovarian follicle recruitment into the growing pool, consistent with AR-mediated stimulation of ovarian primordial and primary preantral follicle growth (287-290).

Enlarged theca cell layer or enhanced ovarian androgenic response to human chorionic gonadotropin (hCG) challenge are demonstrated by PNA NHP (Table 1), as well as by mouse genetic models overexpressing LH-β, hCG-β and plasminogen activator inhibitor type 1 (PAI-1) (Table 3). Increased theca cell gene expression for the androgenic enzyme *CYP17A1* and increased androstenedione response to theca cell LH stimulation *in vitro*, however, are reported for PNA sheep, but only in late gestation retrieved fetuses (291), not adults.

At the ovarian level, PNA results in polyfollicular ovarian morphology in sheep (58, 59). Detailed stereological and sonographic surveillance at multiple developmental time points in sheep have provided documentation of increased ovarian follicular recruitment/depletion and persistence (60, 61), emulating the dynamic ovarian follicle findings in women with PCOS from recent, longitudinal, ultrasonography studies (292). Sheep, like humans, are precocial with follicular differentiation completed *in utero*. In-depth evaluations carried out with ovaries of PNA sheep have revealed disruptions in AR/ER ratios (62), growth factor expression such as activin and follistatin (58), and insulin receptor signaling (293), AMH expression levels (63), apoptotic factors (294), steroidogenesis (64), angiogenesis (295), and cell-specific changes in epigenetic modifying enzymes (296).

PAMH mouse female offspring exhibit delayed onset of puberty and severely disrupted estrous cyclicity (oligo-anovulation) and impaired fertility in adulthood (107). Consistent with the anovulatory phenotype, prenatal treatment with AMH results in a polyfollicular ovary (107, 131), characterized by diminished postovulation corpora lutea. This polyfollicular phenotype is likely the consequence of arrested antral follicular development as suggested by observations that prenatal AMH treatment lowers the percentage of late antral follicles in the ovaries of adult offspring. While the ovaries of PNA mice are not polyfollicular, developing follicles display reduced granulosa cell layer thickness and increased theca cell layer thickness. Consistent with their severely impaired estrous cyclicity, PNA mice have significantly fewer preovulatory follicles and few to no corpora lutea (97).

#### Peripubertal and genetic models.

Postnatal exposure of female mice and rats to DHT from 3 weeks of age for 3 months leads to the development of ovulatory dysfunction as ovaries lack or show few corpora lutea (96, 115, 122, 124). Peripubertal onset T in female NHP supplemented with a high fat diet (T+DIO), however, does lead to luteal insufficiency (112), suggesting impaired dominant follicle maturation prior to ovulation and a potential for subsequent cycle disruption. These changes are associated with diminished ovarian vascular perfusion likely compromising function of both the preovulatory dominant follicle and the subsequent corpus luteum (110). Histologically, ovaries display the classic polycystic appearance with numerous atretic cyst-like follicles present, but opposite to what is seen with women with PCOS, ovarian weight is unaltered or decreased (96, 115, 122, 124, 244). Additional ovarian PCOS-associated features include an increase in the proportion of morphologically unhealthy follicles, and within large antral follicles a reduction in granulosa cell-layer thickness, but an increase in theca cell-layer area, presumably a consequence of the reduction in corpora lutea populations (96, 115, 122, 124).

Letrozole exposure prepubertally in mice and rats for 3 months leads to the formation of ovarian cysts, thickening of the theca cell layer and a thin granulosa cell layer. This is accompanied by an increased ovarian weight and complete disruption of estrous cyclicity with a constant pseudodiestrus and few or no corpora lutea in the ovaries (119, 120, 123, 132, 134, 228).

Ovaries from transgenic mice overexpressing NGF in thecal-interstitial cells produce more NGF than wild-type ovaries and are hyperinnervated by sympathetic nerves. Adult mice overexpressing NGF display ovulatory dysfunction, with more time spent in estrus and less time spent in diestrus. The ovarian morphology is characterized by granulosa cell apoptosis, arrested antral follicle growth, and an increased number of antral follicles, many of which are atretic (137, 138, 297)

### Ovarian steroidogenesis

#### Prenatal models.

Functional ovarian hyperandrogenism has only been identified in PNA NHP from intramuscular injection of recombinant hCG during the follicular phase or anovulatory period resulting in an LH-receptor–mediated elevated increase in T (298). Baseline circulating levels of T, however, are elevated in PNA NHP, sheep, and mice, as well as in PAMH mice (Table 1), and estradiol levels can diminish in the late reproductive years of PNA NHP (299). Hyperandrogenism in PNA sheep is sufficiently manifest as to induce hirsutism (65), an aspect not yet addressed in other PCOS models. In addition, PNA NHP exhibit adrenal androgen excess (298, 300), emulating that found in women with PCOS (301).

#### Peripubertal and genetic models.

Consistent with anovulation, circulating progesterone and estradiol levels in adulthood are significantly reduced in postnatal DHT-induced PCOS-like mice (96, 244), while T can be decreased, likely due to negative feedback from estrogenic metabolites of DHT (96, 302). Furthermore, androgen exposure in this model intrinsically disrupts ovarian steroidogenesis. After isolation of antral follicles and 5 day *in vitro* culture DHT-exposed follicles secreted elevated levels of progesterone (125) In the ovary, DHT exposure increases *CYP17A1* and *HSD3B2* expression and decreases *CYP19A1* expression. This may further enhance androgen production and lead to less androgen being converted to estrogen by aromatase, but it is not known which cell type contributes to the difference in gene expression (244). In isolated follicles from DHT-exposed mice, gene expression for the steroidogenic enzymes *Cyp11a1* and *Cyp17a1* is upregulated and downregulated, respectively (125). Interestingly, NGF overexpression in mouse granulosa cells causes increased circulating T, progesterone and estradiol levels in adulthood (138, 297), suggesting that upregulation of one or more ovarian growth factors may contribute to PCOS-like ovarian hyperandrogenism. The increased steroid production is associated with increased expression of *Hsd17b, Cyp17a1*, and *Cyp19a1*, which enhance steroidogenesis (297).

### Oocyte and fertility

Impaired oocyte quality is inconsistent among women with PCOS, likely due to variations in specific PCOS phenotype and associated comorbidities observed between PCOS patients which differentially alter oocyte quality (303). Women with classic PCOS are most likely to exhibit abnormal intrafollicular environments and impaired oocyte development (304), possibly related to androgen and insulin excess or other metabolic disruptors. This is important, as hyperandrogenic follicles of classic PCOS women undergoing ovarian stimulation for in vitro fertilization (IVF) (305) contain morphologically normal MII oocytes with abnormal gene expression (192). These genes often contain promoter sequences with putative binding sites for AR and peroxisome proliferating receptor gamma, which link androgen excess with insulin resistance during oocyte development. With androgen and insulin levels in the follicles of patients with IVF determined by PCOS and BMI, respectively (305, 306), normal-appearing embryo transfers from obese patients with PCOS have a high miscarriage rate after transfer into a surrogate uterus of a woman without PCOS (307). On the other hand, oocytes from nonobese IVF patients with PCOS by Rotterdam criteria are smaller and less likely to mature than oocytes from nonobese IVF patients with male factor infertility, despite similar clinical pregnancy and live-birth rates/cycle in the 2 female groups (308). Given the necessary ethical constraints on manipulating human oocytes, PCOS-like animal models provide unique opportunities to explore the underlying mechanisms governing oocyte developmental competence.

#### Prenatal models.

Subfertility exists in early- to mid-gestation PNA NHP, sheep, and mice, as well as PAMH mice (Table 1). In PNA sheep, increased recruitment of primordial follicles and increased size of oocytes within them (59), suggest that altered preantral granulosa cell-oocyte signaling may contribute to diminished oocyte quality at the very onset of follicle growth and oocyte development within growing follicles. This is reflected as reduced breeding success in PNA sheep (66). In PNA NHP undergoing ovarian stimulation for IVF, morphologically normal MII oocytes and their fertilization rates are comparable to age- and BMI-matched controls (168), but oocyte developmental competency is diminished, particularly in early- to mid-PNA NHP (see “Preimplantation embryo” below). It is unknown whether dysfunctional ovarian follicle development in PAMH mice contributes to infertility through diminished oocyte quality.

#### Peripubertal and genetic models.

Culture of isolated preantral and antral follicles from DHT-exposed PCOS mice results in slower growth rates and reduced follicle health and survival rates compared with controls (125). In contrast, preovulatory follicles from DHT-exposed mice exhibit a significant increase in growth rate, but also with reduced health and survival rates. The impaired oocyte function in DHT-exposed mice is also evident by increased levels of reactive oxygen species, a poorer response to hyperstimulation, and a reduction in on-time embryo development (125). Comparable fertility deficits appear in peripubertal T-onset NHP with delays in first age to conceive and diminished quality of oocytes obtained following ovarian stimulation for IVF (110).

Transgenic NGF mice exhibit delayed puberty as assessed by a delayed vaginal opening and delayed first estrus. They also exhibit reduced fertility, as assessed by a prolonged interval between litters, a reduction in the number of litters born, and a reduced number of pups per litter (137). Other genetically modified models such as Lhb, hCGb subunit and prohibitin overexpressing, and IR/LepR^POMC^ knockout mice, also exhibit decreased fertility or fecundity (Supplementary Table 1a) (36).

#### Adult and naturally hyperandrogenic models.

T administration to adult female NHP increases the number of primary, growing preantral and small antral follicles and the proliferation of granulosa cells within them (288, 290). Androgen treatment in such monkeys upregulates mRNA expression of FSH receptor, IGF1 receptor, and IGF1 in granulosa cells (287, 289), and enhances IGF1 and IGF1 receptor mRNA expression in primordial follicle oocytes (309). The ability of such T-exposed oocytes to mature and acquire developmental competence following ovulation is unknown. But given the findings from peripubertal T-onset NHP models, above, it is likely that such qualities are compromised. Such diminished oocyte quality is further supported by naturally hyperandrogenic female NHP with the highest circulating T levels being subfertile, although it is unclear whether this is an issue of impaired oocyte quality or altered endometrial development (37).

### Preimplantation embryo

#### Prenatal models.

Successful oocytes in PNA NHP only develop to blastocyst after IVF when they originate from dominant follicles exhibiting follicular fluid levels of estradiol relative to the progesterone-to-estradiol ratio within the 95% confidence interval for the relationship in control females (42). The percentage of successful oocytes obtained from early- to mid-gestation PNA NHP, however, was < 20%, suggesting considerable impairment of developmental competency and diminished embryonic genome activation at the 5- to 8-cell stage (168). Thus, within a PCOS-like NHP ovary, there is considerable variation in compromised follicle function such that some follicles appear functionally normal and will yield successful oocytes. Although oocyte quality has not been examined in PNA sheep, reduced fertility in mating studies are suggestive of compromised oocyte quality in this model as well (66). Together, these findings may provide one explanation for the inconsistency in oocyte quality found in women with PCOS.

#### Peripubertal and genetic models.

Oocytes from peripubertal DHT-exposed mice appear morphologically normal after collection following ovarian stimulation and IVF (125). Hyperandrogenism, however, leads to aberrant embryo development as fertilization rates are decreased after IVF and a reduction in on-time embryo development is observed compared to oocytes collected from control females (125). Peripubertal T onset in female NHP results in poor embryo quality from oocytes obtained following ovarian stimulation for IVF in comparison to controls (110). Fertilized oocytes exhibit poor rates of progression to morula and blastocyst stages. Mild uterine endometrial progesterone resistance may also contribute to pregnancy delay and pregnancy loss, particularly when peripubertal T onset monkeys are supplemented with high-fat Western-style diet (T+DIO) (110).

### Pregnancy

#### PNA and PAMH models.

Pregnant women with PCOS are at increased risk for various pregnancy complications, including gestational diabetes and life-threatening pre-eclampsia (210-212). Their placentae exhibit structural and molecular dysfunction (213, 310, 311), including increased signal transducers and activators of transcription 3 (STAT3) phosphorylation, indicating that specific metabolic pathways are activated by increased maternal androgen concentrations (215). Diminished placental expression of aromatase (*CYP19A1*) and *HSD3B1* likely impairs its ability to metabolize androgens (216). Not surprisingly, therefore, maternal and fetal environments of all PNA and PAMH animal models are compromised. Pathophysiological and morphological insults vary, however, depending on species and when during gestation T or DHT exposure occurs (Tables 1-2), and are more pronounced in early- to mid-gestation PNA NHP models, at least when maternal BMI is higher before conception (312). For one example, the hyperandrogenic maternal environment of early- to mid-gestation PNA female Suffolk sheep manifests progesterone insufficiency, together with hyperinsulinemia and decreased circulating levels of medium chain acylcarnitine (67). The latter finding suggests some maternal counter-adjustment to metabolic compromise, since elevated acylcarnitines commonly accompany metabolically compromised pregnancies in humans (313). Not surprisingly, placental efficiency in PNA sheep is reduced (68) and early placental defects are evident by 65 days of gestation in a 147-day pregnancy, including increased placental accumulation of lipid and collagen together with female fetus-specific disruptions in proinflammatory markers and hypoxia inducible factor 1 (69).

Analogous placental compromise is found in the PAMH mouse model. Dam AMH treatment during late gestation likely acts through placental AMHR2 (314) to inhibit placental expression of *CYP19A1* and *HSD3B1*, exaggerating maternal increases in T bioavailability and decreasing circulating estradiol and progesterone levels. In line with these observations, dams of PNA rats develop increased STAT3 protein signaling in the placenta, likely diminishing placental amino acid transport and enabling fetal growth restriction (86). PAMH fetuses certainly experience a higher incidence of abortion. Maternal progesterone and estradiol levels in dams of PNA NHP, in contrast, are not perturbed by maternal hyperandrogenism; transient maternal postprandial hyperglycemia, however, accompanies excessive mid-gestation weight gain (312). PNA/PAMH promise to be excellent models in which to investigate androgen-induced placental dysfunction, including placental cell-specific expression of GFP, as already utilized when investigating neuroendocrine function (263, 264).

Fetal female endocrine environments in early- to mid-gestation PNA NHP and sheep, as well as PNA and PAMH mice, are hyperandrogenic (T levels are typical of male fetuses), but only those in PNA sheep (67, 70) and PAMH mice exhibit additional estrogenic perturbations. The more robust NHP placenta with its greater aromatase expression may better equip it to endure maternal hyperandrogenism and accompanying metabolic sequelae (41). In addition, fetal female early- to mid-gestation PNA NHP and sheep experience hyperglycemia combined with hypolipidemia, and yet this environment accompanies components of fetal growth restriction in PNA sheep (71) compared to normal or increased fetal growth in PNA NHP (312). Like PNA sheep (68), PNA mice and rats have decreased placenta and fetal weight (99, 315), together with fetal growth restriction (86). Dam hyperandrogenemia thus not only provides fetal androgenic endocrine disruption, but also perturbs nutrient provision across a compromised placenta.

As pregnant women with PCOS are often obese, 2 mouse (and 2 NHP, see below) models were combined: maternal DIO and PNA (99, 100). These DIO PNA mice develop maternal and fetal liver dysfunction with high triglyceride content and altered expression of transcription factors and enzymes involved in *de novo* lipogenesis and fat storage (99). PNA, but not maternal DIO fetuses, have a higher male, but not female, fetus/placenta weight ratio. Furthermore, total and phosphorylated proteomic analyses of placenta and fetal liver of DIO and/or PNA female mice identified a novel target, the phosphorylation of catechol-O-methyltransferase (COMT) (100). Maternal obesity apparently increases the activation of catecholamine metabolism whereas maternal androgen exposure decreases it.

#### Peripubertal models.

Peripubertal T onset in female NHP models compromises their subsequent pregnancies, particularly when supplemented with a peripubertal Western-style diet-induced obesity, T+DIO (Table 2). While fecundity was 100% in control and T-treated female groups, it fell to < 50% in T+DIO females (110), but there were no accompanying significant increases in gestational weight gain in either T-treated group. Placental blood flow was reduced in T-treated females, yet surprisingly, both fetal weight and abdominal circumference were greater relative to maternal weight in T+DIO females. Interestingly in this regard, T+DIO females exhibited gestational hyperinsulinemia and hyperglycemia as a result of gestational insulin resistance. Circulating lipids, nevertheless, were normal in both T-treated groups, except for elevated HDL levels (110). Circulating estradiol and androstenedione levels increased late in gestation in T+DIO females, while progesterone levels were unremarkable in T-treated groups. Thus, similar to T-treated dams of PNA female NHP, when peripubertal-onset T-treated females become pregnant, their female offspring are exposed to compromised placental function (but without the accompanying reductions in estradiol and progesterone found in nonprimates) and dysfunctional maternal glucoregulation and lipids. Not surprisingly, female offspring of T-treated dams show signs of increased fetal growth and adiposity in late gestation, again resembling PNA female NHP fetal and infant findings, but contrasting with those from PNA female nonprimates.

## Metabolic Dysfunction

Independent of body weight and fat mass, women with PCOS are more likely to be insulin resistant and to have compensatory hyperinsulinemia, placing them at an increased risk of impaired glucose tolerance and T2D (3, 316). Women with PCOS also have dyslipidemia, abdominal obesity, and altered adipose tissue morphology and function (3). Additionally, women with PCOS may also have pancreatic β-cell defects since their insulin secretion does not change proportionally in response to glucose (317).

Insulin resistance in women with PCOS is still not completely understood. Obesity is the most common cause of insulin resistance, and the prevalence rates of PCOS diagnosis increases with obesity (15, 169). However, as discussed above, insulin resistance in women with PCOS occurs independently of obesity, as previously reviewed (3, 169, 316).

Another factor linked to metabolic dysfunction is nonalcoholic fatty liver disease (NAFLD) and clinical studies and meta-analyzes indicate higher prevalence of NAFLD and nonalcoholic steatohepatitis (NASH) in women with PCOS (318-321). There seems to be an interplay between insulin resistance, hyperandrogenism, increased abdominal fat, and NAFLD and NASH in women with PCOS, but the mechanisms are largely unknown.

### Metabolic neural regulation

#### PNA models.

Both the ventromedial nucleus and the arcuate nucleus within the mediobasal hypothalamus play crucial roles in the neural regulation of metabolic function. In female rodents, estrogen signaling via ESR1 in the ventromedial nucleus regulates thermogenesis and locomotory activity, and thus adiposity, involving leptin responsive neurons expressing steroid factor-1 (SF1) (322). In the arcuate nucleus, in contrast, ESR-1–mediated signaling regulates calorie intake, and thus appetite, involving POMC and agouti-related peptide (AgRP), expressing neurons. AgRP neurons co-localize another orexigenic peptide, neuropeptide Y (NPY), whereas POMC neurons co-localize the anorexigenic peptide, cocaine and amphetamine-regulated transcript (CART). AgRP ⁄ NPY and POMC ⁄ CART neurons have also been shown to express receptors for both insulin and leptin (322), and interestingly, most AgRP ⁄ NPY and POMC ⁄ CART neurons in the arcuate nucleus express AR (323). At least in female rodents, T- or DHT-mediated hypothalamic AR action diminishes ESR1-mediated signaling. The potential for developmental hypothalamic programming of metabolic regulation was realized in PNA sheep when Lehman and colleagues demonstrated increased numbers of arcuate nucleus AgRP ⁄ NPY neurons, suggesting a possible substrate through which T may directly influence energy balance at the level of the hypothalamus in these PCOS-like PNA ewes (323). Since co-treatment with the AR antagonist flutamide blocks T- or DHT-mediated increase in number of AgRP-immunoreactive neurons, PNA may predominantly exert its action on AgRP neurons through AR rather than ER.

In the PNA mouse model, NPY and beta-endorphin (POMC) neuron cell numbers in the arcuate nucleus are not different (261). In addition to their well-known role in promoting food intake, NPY neurons in the arcuate nucleus are also implicated in coordinating energy homeostasis with fertility by responding to peripheral metabolic cues and signaling within the GnRH neuronal network (324). Optogenetic and chemogenetic activation of arcuate nucleus NPY neurons reduces pulsatile LH secretion in both PNA mice and healthy controls (325), through a mechanism that likely involves kisspeptin neurons (326). Whether hypersecretion of LH is linked to impaired NPY signaling within the GnRH neuronal network remains to be determined, but this work highlights activation of NPY as a potential mechanism for slowing the hyperactive GnRH/LH axis.

### Insulin resistance, compromised glucose homeostasis, and pancreatic β-cell dysfunction

#### PNA and PAMH models.

Metabolic perturbations in PNA sheep include systemic increases in peripheral insulin resistance, resulting in postnatal compensatory hyperinsulinemia in gestation days 30-90 and 60-90 PNA Suffolk sheep (72) and postnatal dyslipidemia in gestation days 30-90 PNA sheep (73, 74). Metabolic defects in gestation 30-90 day PNA sheep include tissue-specific changes in insulin resistance with liver and muscle being insulin-resistant (327), reduced adipogenesis and increased proportion of small adipocytes (73, 328), and compromised fetal pancreatic differentiation (75). Comparable glucoregulatory pathophysiology is also found in early- to mid-gestation PNA NHP, with dysregulation detectable during infancy (312, 329). Specific changes in insulin-target tissues in PNA sheep include increased lipid accumulation in liver and muscle, an increase in nitrotyrosine (an oxidative stress marker in liver and visceral adipose tissue) (74), and disrupted intracrine steroid balance in visceral adipocytes (330). Lipid accumulation in the liver is suggestive of NAFLD-like hepatic phenotype, while the increased distribution of small adipocytes is similar to that observed in early- to mid-gestation PNA NHP (331) and lean PCOS subjects (220, 331).

In concert, these studies provide a possible linkage for tissue-specific defects contributing to PCOS-like metabolic phenotype, including hyperandrogenism-mediated, adipogenic constraint (331), that likely promotes insulin resistance and pancreatic β-cell defects through lipotoxicity (221). Specifically, androgen inhibits early-stage human subcutaneous (SC) abdominal adipogenesis *in vitro*(332). A similar event *in vivo* could therefore constrain SC fat storage to promote insulin resistance through lipotoxicity from ectopic lipid accumulation in nonadipose tissues (332). This mechanism agrees with findings in normal-weight PCOS patients of altered SC abdominal adipocyte stem cell gene expression of adipogenic/angiogenic functions involving T through TGF-β signaling (221), as well as in infant and adult visceral adipose tissue from early- to mid-gestation PNA NHP of differentially methylated genes involving TGF-β signaling (333), supporting a role for epigenomic programming in the etiology of PCOS.

PNA mice present a mild metabolic phenotype, including increased fasting glucose level and impaired glucose tolerance but normal insulin sensitivity, and an early form of islet dysfunction in the pancreas (96, 101). Furthermore, the PNA mouse model also displays hepatic steatosis with increased hepatic triglyceride and lipid content, and dysregulated expression of enzymes involved in lipogenesis (99, 102). These aberrant metabolic findings are present despite a lack of change in glucose metabolism. When combining the PNA model with maternal DIO, it becomes evident that the metabolic phenotype is largely due to PNA exposure and, to a lesser extent, maternal DIO (102).

PNA rat offspring, on the other hand, are insulin-resistant as measured with the highly sensitive euglycemic hyperinsulinemic clamp method (87). Decreased insulin sensitivity was prevented when flutamide or tamoxifen was administered simultaneously with prenatal T, indicating that both AR and ER are involved in the mechanism regulating whole-body glucose homeostasis (87). Of note, the dose of T and timing of administration are key factors. When a 10-times higher dose of T was given to pregnant dams, offspring did not develop insulin resistance (315), but they did develop lipid disturbances and hepatic steatosis (315).

#### Peripubertal and genetic models.

Peripubertal DHT-induced PCOS female rats and mice exhibit elevated fasting blood glucose levels (116, 124, 125), decreased glucose tolerance (116, 244), and development of insulin resistance at adult age as measured by euglycemic hyperinsulinemic clamp, oral glucose tolerance test, and insulin tolerance test (96, 115, 122-125, 244). In line with increased fasting plasma insulin levels, insulin expression tends to be increased in pancreatic β-cells from adult DHT-exposed mice, and in combination with decreased expression of *Glut2*, this may be an early indicator of β-cell dysfunction (244). In an initial effort to dissect out the key sites of AR action, one study assessed fasting glucose levels in a DHT-induced PCOS model combined with global ARKO mice, or mice lacking AR in the brain and/or granulosa cells. Apart from the global AR knockout mouse, however, no other model has so far been able to protect against the development of increased fasting glucose levels (Supplementary Table 2) (36). Further investigations are required to elucidate the mechanisms driving insulin resistance in PCOS, including the role of AR actions in skeletal muscle, liver, and adipocytes, as well as the interplay of diet (334). For example, in a recent study combining the adult DHT-induced model in pancreatic β-cell specific (βARKO^RIP^) and neuron specific (nARKO) models together with a Western diet (DIO) as a metabolic stress, it was shown that DHT exposure predisposes to T2D via activation of hypothalamic AR causing hepatic insulin resistance, and activation of pancreatic β-cell AR leading to increased insulin secretion and β-cell failure (130).

Adiponectin-overexpressing mice are protected against peripubertal onset DHT-induced insulin resistance and glucose intolerance, supporting the idea that adiponectin plays a crucial role in whole-body glucose homeostasis in PCOS (244). Continuous DHT exposure, however, does not increase pancreas β-cell insulin expression in mice overexpressing adiponectin (244). In adult mice, continuous DHT exposure initiated in adulthood drives metabolic dysfunction and causes hepatocyte insulin resistance by increasing gluconeogenesis via transcriptional regulation of gluconeogenic coactivators and enzymes (128). Administration of flutamide reverses DHT-induced hepatocyte insulin resistance, supporting an AR-driven mechanism (128).

Peripubertal letrozole-induced PCOS rats and mice also have increased fasting glucose and insulin levels and develop insulin resistance in adulthood as measured by insulin tolerance test and euglycemic hyperinsulinemic clamp (119, 120, 123, 132, 134, 135, 228). In contrast to elevated fasting insulin levels, peripubertal letrozole-induced PCOS mice display a blunted response to glucose-stimulated insulin release, suggesting β-cell dysfunction (135).

Ovarian NGF overexpression leads to hyperinsulinemia and insulin resistance, but with a preserved hepatic insulin sensitivity and normal glucose production (138). Sympathetic overactivity has adverse effects on glucose homeostasis with impaired glucose tolerance in NGF overexpressing mice (138).

### Body composition and adipose tissue function and whole-body metabolism

#### PNA and PAMH models.

PNA models, except for NHP, have low birthweights. At weaning, nevertheless, PNA sheep, together with PNA and PAMH mice, display normal body weight, while PNA rat offspring weigh more than controls, with no difference in weight at adult age, indicating that PNA sheep and rat manifest postnatal catch-up growth (10, 76, 87, 102, 107). In analogous fashion to postnatal catch-up growth, early- to mid-gestation PNA NHP exhibit increased postnatal weight gain despite a normal birthweight (312), possibly mimicking an altered developmental trajectory that is associated with increased risk for PCOS in humans (335). When adult, early- to mid-gestation PNA NHP exhibit increased intra-abdominal visceral adiposity, hyperlipidemia, relative increases in small adipocytes (constrained adipogenesis) in subcutaneous adipose, and visceral adiposity positively correlated with basal insulin levels (280, 331, 336), while late gestation PNA NHP in adulthood demonstrate increased whole body adiposity in the absence of insulin resistance (337). In addition, early- to mid-gestation PNA NHP adults exhibit increased subcutaneous abdominal adipose *Zfp423* gene expression, but decreased *C/EBPalpha* expression, suggesting PNA-driven organizational increases in commitment of adipogenic stem cells to preadipocytes together with their subsequent diminished progression to mature adipocytes, thus constraining safe lipid storage with increasing BMI (331). Such a mechanism could result in early- to mid-gestation PNA NHP hyperlipidemia-induced increases in visceral fat accumulation, insulin resistance and pancreatic β-cell failure, culminating in increased T2D (331), and all would be consistent with an accompanying positive correlation between visceral fat and basal insulin levels (337).

Conflicting data exist whether PNA mice have more body fat with one study demonstrating more fat mass (measured with EchoMRI) and enlarged adipocytes (101), whereas another study found no difference in fat mass and adipocyte size (102). Furthermore, markers involved in adipogenesis, such as *ZfP423, Cebpa,* and *Cebpb,* have been found to be differently expressed, suggesting that PNA mice can be used to investigate adipose tissue function reflecting daughters of women with PCOS.

Using indirect calorimetry for metabolic phenotyping, PNA mice demonstrate reduced energy expenditure and lower respiratory exchange ratio, likely reflecting preferential fatty acid instead of carbohydrate consumption, and likely also reflect their increased fat mass.

#### Peripubertal and genetic models.

NHP exposed to peripubertal T onset, and supplemented with a high fat diet (T + DIO), partially emulate the metabolic outcomes of early- to mid-gestation PNA NHP. Peripubertal onset T + DIO NHP demonstrate increased abdominal “android” fat and abdominal circumference indicative of increased visceral adiposity (110). Diminished basal lipolysis in both subcutaneous and visceral abdominal fat depots co-occurs with augmented insulin-mediated FFA uptake into visceral adipocytes, alone, contributing to enlarged visceral, but not subcutaneous, adipocytes (113). Since adrenergic (sympathetic nervous system noradrenalin) stimulation of lipolysis is also diminished, but only in subcutaneous adipocytes, unaffected adrenergic stimulation of lipolysis in enlarged visceral adipocytes likely contributes to increased lipid release into the liver, with subsequent adiposity-associated insulin resistance and compensatory hyperinsulinemia (113). T + DIO NHP thus demonstrate the need for the onset of both T and DIO during adolescence to evoke an equivalent of the metabolic derangements engaged by early- to mid-gestation PNA, alone (Tables 1 and 2). Peripubertal T onset without DIO does not induce metabolic dysfunction and weight gain in female NHP (110, 113). Taken together, these peripubertal T findings in female NHP may provide support for early clinical intervention with young women with PCOS: prevent or counteract diet-enabled weight gain, and any remaining hyperandrogenism may pose little metabolic risk.

The peripubertal DHT-induced PCOS mouse model has a higher body weight, percentage body fat and lean body mass, but a decrease in bone mineral density (96). Adipocyte size is increased while adipocyte function is defective, as reflected by decreased circulating adiponectin levels (124, 244). Furthermore, DHT-exposed adipose tissue shows decreased expression of genes involved in metabolic pathways such as adiponectin receptor, insulin receptor, *Ppar gamma*, and *Chrebp* in the DHT-exposed mice (244). There are, however, no alterations in these parameters in adiponectin-overexpressing mice exposed to DHT and in mice with a brain-specific loss of AR. Adiponectin transgenic mice appear to be protected against DHT-induced adipocyte hypertrophy by virtue of smaller adipocytes (124, 244).

Peripubertal letrozole-induced mice or rat models have higher body weight, lean mass, and fat mass, with a pronounced increase in subcutaneous obesity in rats, and also increased visceral fat in letrozole-exposed mice (119, 120, 132, 134, 228). The increased fat mass in letrozole-exposed rats and mice is accompanied by enlarged adipocytes in both the visceral and subcutaneous fat depots (120, 134). Circulating levels of adiponectin are decreased, while leptin and serum triglycerides are elevated (135, 228). These findings, combined with an upregulation of several genes involved in regulating whole-body insulin sensitivity and lipogenesis in subcutaneous fat in letrozole-treated rats, along with increased expression levels of several inflammatory factors and cytokines in letrozole-treated mice, indicate an adipose tissue dysfunction in the letrozole-exposed PCOS model (132, 228). Furthermore, letrozole treatment is associated with dyslipidemia, including elevated total cholesterol, triglycerides, and free fatty acids (135). Indirect calorimetry of the peripubertal letrozole-induced PCOS model revealed no significant difference for food intake, day and night oxygen consumption, carbon dioxide expulsion, and respiratory quotient (120, 228). Nighttime locomotor activity was decreased in the letrozole model (rodents are nocturnally active) (135). The variable metabolic phenotypes observed with peripubertal DHT and letrozole-induced PCOS-like animal models compared with PNA emphasize the need to recognize contributions from activational signals that occur throughout postnatal life (ie, pubertal events, dietary choices, stress, and exposure to environmental chemicals), and that these play crucial roles in inducing pathophysiology, modifying the expression of any programmed (ie, re-organized) phenotype, and/or amplifying the severity of phenotypic expression.

Overexpression of NGF in the ovary results in increased body weight at 5 weeks of age and onwards, accompanied by increased fat mass with a higher visceral fat distribution (138). Analysis of body composition also shows an increase in lean mass and bone mineral content and density (138).

## Cardiac Dysfunction

All factors listed above are linked to cardiovascular disease and studies have demonstrated that women with PCOS can have increased left ventricular mass (338, 339), endothelial dysfunction (339), arterial stiffness (340), and coagulation and fibrinolytic disturbances (341), all surrogate markers of cardiovascular disease. Despite these dysfunctions, there are limited data demonstrating whether women with PCOS are at an increased risk of cardiovascular disease events and the underlying pathophysiology is largely unknown (342, 343).

### PNA and peripubertal models

Telemetry studies carried out in Suffolk sheep found PNA from days 30 to 90 of gestation leads to hypertension (77), adverse left ventricular remodeling that includes increased expression of genes and proteins involved in left ventricular hypertrophy and stress, together with histological evidence of focal myocardial disarray and an increase in cardiomyocyte size (78).

In adult PNA mice, despite cardiac hypertrophy, there is no difference in heart rate or blood pressure (102). The expression of genes involved in cardiac hypertrophy is upregulated in the adult PNA offspring, while neonatal PNA hearts display an upregulation of transcription factors involved in cardiac hypertrophic remodeling and of genes related to calcium signaling and oxidative stress (102). Also, peripubertal DHT exposure in mice leads to adverse left ventricular remodeling, and increased vasocontractile responses (102), as well as an increase in systolic blood pressure (124). Peripubertal DHT–exposed rats also have endothelial dysfunction assessed by vasodilatory response of preconstructed arteries to acetylcholine, even when controlling for increased body weight/fat by pair feeding (344). Simultaneous administration of flutamide from peripuberty (102), or treatment with flutamide or metformin in adulthood after developing the PCOS phenotype, partly reversed these effects demonstrating the involvement of AR (102, 344). Altogether, these observations point towards an androgenic mechanism that is independent from age at administration, although peripubertal onset DHT and letrozole PCOS-like models, with continuous treatment through puberty and adulthood, can result in distinct phenotypes that are not observed in PNA offspring and vice versa.

## Behavior

Women with PCOS are at an increased risk for a wide range of psychiatric disorders (345). The most prevalent symptoms in women with PCOS are depression and anxiety independent of obesity (346, 347). Despite the high prevalence, the cause of depressive and anxiety symptoms is not fully elucidated (347). Clinical signs of hyperandrogenism including acne and hirsutism, as well as infertility and high BMI, have been linked to symptoms of anxiety and depression (347). Recent studies highlight the role of the prenatal period in the etiology of neuropsychiatric disorders in children (217, 345, 348-350). Women with PCOS have elevated maternal androgens and thereby increase the potential risk of exposing the developing fetus to androgens. These findings need further mechanistic studies.

### PNA models

Suffolk PNA sheep manifest behavioural changes such as male-typical courting and mating behaviours consistent with defeminisation/masculinization (79) leading to increase in mounting behavior (79, 80). PNA NHP exhibit comparable alterations to female behavior prepubertally and in adulthood (351), and in NHP, such PNA re-organization of behavior is AR- and not ER-mediated (352). Using the PNA model with T exposure in rats, increased anxiety-like behavior could be prevented by simultaneous treatment with flutamide and partly also by tamoxifen, implicating that AR, and partly ER, mediate alterations in behavior (87). The anxiety-like behavior coincides with changes in gene expression involving steroid receptors in key brain areas, in particular the amygdala (87). AR expression is decreased in the amygdala, suggestive of a downregulation of androgen signaling, while the serotonin 2c and GABA_A_ receptors are elevated in PNA females of PCOS-like dams. These data highlight the amygdala as a potentially important site for developmental changes that lead to long-term behavioral consequences downstream from PNA exposure and PCOS-like development.

Studies implicating subtle cognitive impairments in women with PCOS (353, 354) have implicated hyperandrogenism and metabolic dysfunction, respectively, as causal mechanisms. In addition, recent findings in mice have functionally associated hippocampal expression of both AMH and AMHR2 with neuronal function essential for memory acquisition, retention, and recall in both males and females, with greater hippocampal AMH expression in females (355). Given that gene variants of AMH and AMHR2 with diminished bioactivity have recently been associated with ~3% of familial PCOS (5, 6), that neuronal AMH and AMHR2 expression have been associated with embryonic neuronal differentiation in both humans and mice (106), and that PAMH female mice exhibit many PCOS-like traits (197), altered AMH signaling may also contribute to cognitive differences in women with PCOS. Animal models expressing altered AMH or AMHR2 expression, including genetically manipulated and PAMH mice, together with naturally occurring PCOS-like NHP, potentially promise valuable mechanistic insights.

As obesity is a common comorbidity in women with PCOS, the PNA mouse model, with DHT exposure to specifically target the AR receptor, was combined with maternal DIO to investigate the impact of these 2 factors. Independent of diet, female PNA-exposed offspring develop an anxiety-like behaviour, whereas male offspring from only obese dams also develop an anxiety-like behavior. Differential expression of genes demonstrates that female anxiety-like behavior is linked to upregulation of adrenergic receptor α1b in the amygdala and corticotrophin releasing hormone receptor 2 in the hypothalamus (103), supporting the finding of altered placenta COMT expression (100). Males, on the other hand, display upregulation of genes linked to epigenetic mechanisms in amygdala and corticotrophin-releasing hormone in hypothalamus (103). Taken together, these findings demonstrate sexually dimorphic effects of PNA and maternal DIO on behavior function and gene expression in key brain regions in exposed offspring.

## Transgenerational Perspectives of Androgen Exposure

Pregnant women with PCOS provide the ideal opportunity to investigate the impact of PNA exposure on their female offspring since it has recently been demonstrated that daughters of women with PCOS are at increased risk of developing the diagnosis of PCOS (10). While there is, however, no safe and ethical way to accomplish this, PNA and PAMH animal models provide translatable alternatives. PNA and PAMH mouse models with or without DIO to mimic a key maternal PCOS condition (99, 100) provide key models to investigate transgenerational effects, and whether exposed lineages have increased susceptibility to a PCOS-like reproductive and metabolic phenotype in F1 to F3 offspring. These studies are analogous to those mimicking maternal metabolic syndrome in mice and the transgenerational transmission of metabolic and mitochondrial dysfunction in F1 to F3 offspring (356).

Recently, for the first time, it was demonstrated that PNA mice derived from F0 dams exposed to DHT during late pregnancy, but not maternal DIO, display both reproductive and metabolic PCOS-like phenotypes which are transmitted across generations, that is, transgenerational transmission (10). More specifically, the PNA but not DIO lineage, exhibits longer anogenital distance, irregular cycles, increased fat mass, enlarged adipocytes, and liver triglyceride content, as well as lower respiratory exchange ratio and energy expenditure. PNA lineage female mice may therefore demonstrate constrained switching between lipids and other carbon sources for energy metabolism during fasting, emulating a degree of metabolic inflexibility found in women with PCOS (357). These data show that PCOS-like phenotypic changes are transgenerationally transmitted, F1 to F3, whereas only F1 female offspring from DIO-exposed F0 dams develop a metabolic phenotype, with only minor metabolic consequences in F2 and F3 female offspring.

Of note is that the combination of PNA + DIO in F0 females result in severely compromised fetal development of second generation (F2) offspring. To investigate why the combined PNA + DIO lineage delivers only 1 F2 female offspring, litter size and fetal growth were measured at embryonic day (E)12.5 and E18.5 in a second transgenerational experiment. Fewer and smaller F2 embryos at E12.5 were found in the PNA + DIO lineage as well as in the PNA lineage. By E18.5, all PNA + DIO lineage pregnancies miscarried, whereas fetuses in the PNA lineage were only smaller in body size. There were no alterations in germ cell markers in E12.5 fetal gonads that could explain the detrimental effects. Placental gene expression of transcription factor AP-2, gamma (*Tfap2c*), however, involved in placenta growth and linked to fetal death, was decreased in the PNA + DIO lineage indicating that placenta dysfunction may trigger late miscarriage of F2 pregnancies (10). These findings support clinical studies demonstrating that pregnant women with PCOS are at increased risk for pregnancy complications, including miscarriage (210-214).

To understand the underlying mechanisms of transgenerational transmission of PCOS-like traits, single-cell RNA sequencing of MII oocytes from F1 to F3 female offspring in each lineage was performed and differential gene expression analyses were obtained for all lineages except PNA + DIO lineage. A large number of differentially expressed genes (DEGs) were identified across generations involved in ovarian function, metabolism, and DNA repair, as well as imprinted genes, genes involved in mitochondrial activity and epigenetic regulation. There are no available RNA sequencing or omics data of MII oocytes from daughters of women with and without PCOS to investigate the implication of DEGs in the PNA mouse MII oocytes. Selected DEGs with the largest expression differences across generations in MII oocytes of PNA mice, however, were analyzed in serum of daughters from women with and without PCOS, identifying overexpression of *TIAL1* encoding for a member of a family of RNA-binding proteins that are important in a large number of biological functions relevant for PCOS. Other upregulated genes in mouse MII oocytes and human serum were *FABP5, RNF141,* and *INIP* (10). These identified risk-associated genes could potentially be used to identify whether future generations could develop a PCOS phenotype, although further mechanistic research are required.

These transgenerational findings clearly indicate that intrauterine and/or germ cell factors contribute to the development and transmission of PCOS-like traits. The multiomic area of research is developing rapidly and the fact that it now is possible to sequence not just the transcriptome but also the methylome and nucleosome from the same cell will open up new avenues and likely uncover common molecular features that are altered transgenerationally. To further explore the field of transgenerational transmission of PCOS, other models need to be tested, including the PAMH model.

## Translational Aspects

Animal models that emulate PCOS-like traits have provided and will continue to provide unparalleled opportunities to develop novel therapeutic approaches to PCOS. As currently demonstrated, therapeutic approaches towards ameliorating PCOS in women are equally successful in ameliorating PCOS-like traits in animal models. Insulin sensitizers, for example, are proven therapeutics for improving metabolic and reproductive dysfunction in women with PCOS. They are also effective in improving cycles and androgen and LH levels in PNA mice (358), improving cycles and insulin-mediated glucoregulation in PNA sheep (281), and normalizing cycles while diminishing androgenic responsiveness to an hCG challenge and improving glucoregulation and lipids in PNA NHP (280), establishing a common insulin-driven mechanism exaggerating or causing metabolic and reproductive PCOS-like traits in animal models and PCOS traits in women.

In addition, 7 to 10 days of antiandrogen therapy normalizes progesterone negative feedback on LH pulse frequency in women with PCOS (359), 6 months of antiandrogen therapy normalizes menstrual cycles and reproductive hormones (274), and longer duration of application, commonly with other therapies including insulin sensitizers and oral contraceptives, have proven efficacious in improving menstrual cycle regularity and reproductive hormones (360) and hirsutism (361), respectively. Intriguingly, employing peripubertal or adult blockade of AR signaling in PNA mice has revealed an unanticipated mechanism underlying one aspect of PNA programming, rewired neuronal circuitry of the neuroendocrine hypothalamus driving hypergonadotropism that contributes to hyperandrogenism. This rewiring defect in the PNA female mouse hypothalamus persists from prepuberty and into adulthood (97). Antiandrogen therapy in adulthood normalizes hypothalamic neuronal circuitry and restores normal gonadotropin function and estrous cyclicity (93, 97), demonstrating that prevailing hypothalamic androgen action is required to maintain PNA programmed neuroendocrine pathology through ovary-quiescent prepubertal life. This PNA mouse finding holds potential for the development of antiandrogen therapy in PCOS women that targets the hypothalamus alone, thus avoiding hepatoxicity concerns from systemic administration. Such an approach also has potential for prepubertal administration to prevent the onset of PCOS signs and symptoms. The selective peripubertal DHT onset, ARKO mouse models support this notion, as they clearly demonstrate the key roles for AR in discrete organs, such as the brain, in facilitating the induction of many PCOS-like traits (Supplementary Table 2) (36).

The more recent PAMH model provides evidence suggesting that exacerbation of GnRH neuronal activity/secretion can be the basis for neuroendocrine and neural anomalies that accompany PCOS. Indeed, since the prenatal co-treatment of AMH with a GnRH antagonist successfully prevents the appearance of all PCOS-like neuroendocrine traits in female PAMH offspring, the finding suggests a critical pathogenic role for excess GnRH in developmental programming of PCOS and its maintenance (107). Along this line, Tata and colleagues have provided compelling evidence that intermittent delivery of a GnRH antagonist to adult PAMH mice, successfully restores normal gonadotropin function, LH pulsatility, T levels, ovarian morphology, and estrous cyclicity (107). Thus, based on these findings, pharmacological antagonism aimed at tempering GnRH–LH secretion is an attractive therapeutic strategy to restore ovulation and fertility in women with PCOS characterized by high LH levels (> 75% of women with PCOS), given the fact that GnRH antagonists are already used in the clinic in adult subjects without reported deleterious side effects.

Taken together, animal models consistently implicate androgen excess in the etiopathogenesis of PCOS-like traits in females, from fetal life to adolescence. Developmental programming, whether genetic, environmental (epigenetic), or a combination of both, such as androgen excess-induced epigenetic amplification of genotype, appears key. The variable phenotypes observed with DHT- and letrozole-induced PCOS-like models compared to PNA emphasize the contribution of activational signals that occur during critical times in life, including puberty, and are mediated through diet, stress, and environmental chemical exposure to modify any programmed (re-organized) phenotype and alter its severity of expression. Naturally hyperandrogenic PCOS-like female NHP raise the possibility that not only might PCOS risk genes be highly conserved and have ancient origins in human populations, but they may in fact also have more distant origins in ancestral primates, bestowing survival and pro-fertility traits. Thus, both genetically modified mouse models and familial whole genome sequencing in naturally hyperandrognic female NHP are appearing as elegant animal model techniques enabling molecular understanding of PCOS-like etiopathogenesis that can target or regulate specific tissues and body regions. Such emerging models will provide excellent tools with which to further investigate mechanisms of reproductive dysfunction, insulin resistance in target tissues, constrained adipose storage capacity, fatty liver, pancreatic decompensation and cognitive changes. Additionally, cross-talk between tissues and species will likely be important, particularly when possible sequencing data of tissue from animal models can be overlapped with sequenced data from humans using 1:1 orthologue genes.

## Abbreviations

AGD: anogenital distance
AgRP: agouti-related peptide
AMH: anti-Müllerian hormone
AR: androgen receptor
ARKO: androgen receptor knockout
AVPV: anteroventral periventricular
BMI: body mass index
BMP: bone morphogenic protein
CART: cocaine and amphetamine- regulated transcript
DEG: differentially expressed gene
DHEA: dehydroepiandrosterone
DHT: dihydrotestosterone
DIO: diet-induced obesity
DoHAD: developmental origin of adult disease
ER: estrogen receptor
ESR1: estrogen receptor alpha
FSH: follicle-stimulating hormone
GABA: gamma-aminobutyric acid
GDF: growth differentiation factor
GFP: green fluorescent protein
GnRH: gonadotropin-releasing hormone
GWAS: genome-wide association study
hCG: human chorionic gonadotropin
IVF: in vitro fertilization
KNDy: kisspeptin/neurokinin B/dynorphin
LepR: leptin receptor
LH: luteinizing hormone
MII: metaphase II
NAFLD: nonalcoholic fatty liver disease
NASH: nonalcoholic steatohepatitis
NGF: nerve growth factor
NHP: nonhuman primates
NKCC1: sodium–potassium–chloride cotransporter 1
NPY: neuropeptide Y
PCOM: polycystic ovary morphology
PCOS: polycystic ovary syndrome
POMC: pro-opiomelanocortin
PR: progesterone receptor
T2D: type 2 diabetes
TGF-β: transforming growth factor-β

## Additional Information

***Disclosure*:** Authors have nothing to disclose.

## References

[1] Teede HJ, Misso ML, Costello MF, et al.; International PCOS Network. Recommendations from the international evidence-based guideline for the assessment and management of polycystic ovary syndrome. Fertil Steril. 2018;110(3):364-379. 30033227 10.1016/j.fertnstert.2018.05.004 PMC6939856

[2] Rodgers R, Avery J, Moore V, et al Complex diseases and co-morbidities: polycystic ovary syndrome and type 2 diabetes mellitus. Endocr Connect. 2019;8(3):R71-R75. 30763275 10.1530/EC-18-0502 PMC6410761

[3] Dumesic DA, Oberfield SE, Stener-Victorin E, Marshall JC, Laven JS, Legro RS. Scientific statement on the diagnostic criteria, epidemiology, pathophysiology, and molecular genetics of polycystic ovary syndrome. Endocr Rev. 2015;36(5):487-525. 26426951 10.1210/er.2015-1018 PMC4591526

[4] Fraissinet A, Robin G, Pigny P, Lefebvre T, Catteau-Jonard S, Dewailly D. Use of the serum anti-Müllerian hormone assay as a surrogate for polycystic ovarian morphology: impact on diagnosis and phenotypic classification of polycystic ovary syndrome. Hum Reprod. 2017;32(8):1716-1722. 28854589 10.1093/humrep/dex239

[5] Gorsic LK, Dapas M, Legro RS, Hayes MG, Urbanek M. Functional genetic variation in the anti-Müllerian hormone pathway in women with polycystic ovary syndrome. J Clin Endocrinol Metab. 2019;104(7):2855-2874. 30786001 10.1210/jc.2018-02178 PMC6543512

[6] Gorsic LK, Kosova G, Werstein B, et al. Pathogenic anti-Müllerian hormone variants in polycystic ovary syndrome. J Clin Endocrinol Metab. 2017;102(8):2862-2872. 28505284 10.1210/jc.2017-00612 PMC5546867

[7] Dapas M, Sisk R, Legro RS, Urbanek M, Dunaif A, Hayes MG. Family-based quantitative trait meta-analysis implicates rare noncoding variants in DENND1A in polycystic ovary syndrome. J Clin Endocrinol Metab. 2019; 104(9):3835-3850. 10.1210/jc.2018-02496 PMC6660913 31038695

[8] Crisosto N, Codner E, Maliqueo M, et al. Anti-Müllerian hormone levels in peripubertal daughters of women with polycystic ovary syndrome. J Clin Endocrinol Metab. 2007;92(7):2739-2743. 17488788 10.1210/jc.2007-0267

[9] Crisosto N, Ladrón de Guevara A, Echiburú B, et al. Higher luteinizing hormone levels associated with antimüllerian hormone in postmenarchal daughters of women with polycystic ovary syndrome. Fertil Steril. 2019;111(2):381-388. 30527840 10.1016/j.fertnstert.2018.10.011

[10] Risal S, Pei Y, Lu H, et al. Prenatal androgen exposure and transgenerational susceptibility to polycystic ovary syndrome. Nat Med. 2019;25(12):1894-1904. 31792459 10.1038/s41591-019-0666-1

[11] Legro RS, Driscoll D, Strauss JF 3rd, Fox J, Dunaif A. Evidence for a genetic basis for hyperandrogenemia in polycystic ovary syndrome. Proc Natl Acad Sci U S A. 1998;95(25):14956-14960. 9843997 10.1073/pnas.95.25.14956 PMC24557

[12] Bozdag G, Mumusoglu S, Zengin D, Karabulut E, Yildiz BO. The prevalence and phenotypic features of polycystic ovary syndrome: a systematic review and meta-analysis. Hum Reprod. 2016;31(12):2841-2855. 27664216 10.1093/humrep/dew218

[13] Lizneva D, Suturina L, Walker W, Brakta S, Gavrilova-Jordan L, Azziz R. Criteria, prevalence, and phenotypes of polycystic ovary syndrome. Fertil Steril. 2016;106(1):6-15. 27233760 10.1016/j.fertnstert.2016.05.003

[14] Neven ACH, Laven J, Teede HJ, Boyle JA. A summary on polycystic ovary syndrome: diagnostic criteria, prevalence, clinical manifestations, and management according to the latest international guidelines. Semin Reprod Med. 2018;36(1):5-12. 30189445 10.1055/s-0038-1668085

[15] Kataoka J, Larsson I, Björkman S, Eliasson B, Schmidt J, Stener-Victorin E. Prevalence of polycystic ovary syndrome in women with severe obesity - effects of a structured weight loss programme. Clin Endocrinol (Oxf). 2019;91(6):750-758. 31529511 10.1111/cen.14098

[16] Escobar-Morreale HF . Polycystic ovary syndrome: definition, aetiology, diagnosis and treatment. Nat Rev Endocrinol. 2018;14(5):270-284. 29569621 10.1038/nrendo.2018.24

[17] Azziz R, Marin C, Hoq L, Badamgarav E, Song P. Health care-related economic burden of the polycystic ovary syndrome during the reproductive life span. J Clin Endocrinol Metab. 2005;90(8):4650-4658. 15944216 10.1210/jc.2005-0628

[18] Vink JM, Sadrzadeh S, Lambalk CB, Boomsma DI. Heritability of polycystic ovary syndrome in a Dutch twin-family study. J Clin Endocrinol Metab. 2006;91(6):2100-2104. 16219714 10.1210/jc.2005-1494

[19] Barrett ES, Hoeger KM, Sathyanarayana S, et al. Anogenital distance in newborn daughters of women with polycystic ovary syndrome indicates fetal testosterone exposure. J Dev Orig Health Dis. 2018;9(3):307-314. 29310733 10.1017/S2040174417001118 PMC5997496

[20] Homburg R, Gudi A, Shah A, M Layton A. A novel method to demonstrate that pregnant women with polycystic ovary syndrome hyper-expose their fetus to androgens as a possible stepping stone for the developmental theory of PCOS. A pilot study. Reprod Biol Endocrinol. 2017;15(1):61. 28789693 10.1186/s12958-017-0282-1 PMC5549310

[21] Sir-Petermann T, Codner E, Maliqueo M, et al. Increased anti-Müllerian hormone serum concentrations in prepubertal daughters of women with polycystic ovary syndrome. J Clin Endocrinol Metab. 2006;91(8):3105-3109. 16720659 10.1210/jc.2005-2693

[22] Detti L, Christiansen ME, Francillon L, et al. Serum Anti-Müllerian hormone (AMH) in mothers with polycystic ovary syndrome (PCOS) and their term fetuses. Syst Biol Reprod Med. 2019;65(2):147-154. 30428262 10.1080/19396368.2018.1537385

[23] Abbott DH, Kraynak M, Dumesic DA, Levine JE. In utero androgen excess: a developmental commonality preceding polycystic ovary syndrome? Front Horm Res. 2019;53:1-17. 31499494 10.1159/000494899 PMC6954824

[24] Osuka S, Nakanishi N, Murase T, et al. Animal models of polycystic ovary syndrome: a review of hormone-induced rodent models focused on hypothalamus-pituitary-ovary axis and neuropeptides. Reprod Med Biol. 2019;18(2):151-160. 30996678 10.1002/rmb2.12262 PMC6452010

[25] Dunaif A . Perspectives in polycystic ovary syndrome: from hair to eternity. J Clin Endocrinol Metab. 2016;101(3):759-768. 26908109 10.1210/jc.2015-3780 PMC4803161

[26] Rosenfield RL, Ehrmann DA. The pathogenesis of polycystic ovary syndrome (PCOS): the hypothesis of PCOS as functional ovarian hyperandrogenism revisited. Endocr Rev. 2016;37(5):467-520. 27459230 10.1210/er.2015-1104 PMC5045492

[27] Teede HJ, Joham AE, Paul E, et al. Longitudinal weight gain in women identified with polycystic ovary syndrome: results of an observational study in young women. Obesity (Silver Spring). 2013;21(8):1526-1532. 23818329 10.1002/oby.20213

[28] Lim SS, Hutchison SK, Van Ryswyk E, Norman RJ, Teede HJ, Moran LJ. Lifestyle changes in women with polycystic ovary syndrome. Cochrane Database Syst Rev. 2019;3:CD007506. 30921477 10.1002/14651858.CD007506.pub4 PMC6438659

[29] Maliqueo M, Benrick A, Stener-Victorin E. Rodent models of polycystic ovary syndrome: phenotypic presentation, pathophysiology, and the effects of different interventions. Semin Reprod Med. 2014;32(3):183-193. 24715513 10.1055/s-0034-1371090

[30] Stener-Victorin E, Manti M, Fornes R, Risal S, Lu H, Benrick A. Origins and impact of psychological traits in polycystic ovary syndrome. Med Sci. 2019;7(8):86. 10.3390/medsci7080086 PMC6723772 31387252

[31] Walters KA, Gilchrist RB, Ledger WL, Teede HJ, Handelsman DJ, Campbell RE. New perspectives on the pathogenesis of PCOS: neuroendocrine origins. Trends Endocrinol Metab. 2018;29(12):841-852. 30195991 10.1016/j.tem.2018.08.005

[32] Mahesh VB, Costoff A, Mills TM, Bagnell CA. The polycystic ovary syndrome and experimental models for the study of its pathogenesis. Prog Clin Biol Res. 1982;112 Pt A:301-313. 6219402

[33] Puttabyatappa M, Cardoso RC, Padmanabhan V. Effect of maternal PCOS and PCOS-like phenotype on the offspring’s health. Mol Cell Endocrinol. 2016;435:29-39. 26639019 10.1016/j.mce.2015.11.030 PMC4884168

[34] Ryu Y, Kim SW, Kim YY, Ku SY. Animal Models for human polycystic ovary syndrome (PCOS) focused on the use of indirect hormonal perturbations: a review of the literature. Int J Mol Sci. 2019;20(11):2720. 10.3390/ijms20112720 PMC6600358 31163591

[35] Walters KA, Allan CM, Handelsman DJ. Rodent models for human polycystic ovary syndrome. Biol Reprod. 2012;86:149, 141-112. 22337333 10.1095/biolreprod.111.097808

[36] Animal models to understand the etiology and pathophysiology of polycystic ovary syndrome. Dryad. Deposited February 17, 2020. 10.5061/dryad.zgmsbcc75 PMC7279705 32310267

[37] Abbott DH, Rayome BH, Dumesic DA, et al. Clustering of PCOS-like traits in naturally hyperandrogenic female rhesus monkeys. Hum Reprod. 2017;32(4):923-936. 28333238 10.1093/humrep/dex036 PMC6251677

[38] Abbott DH, Dumesic DA, Levin JE, Dunaif A, Padmanabhan V. Animal models and fetal programming of the polycystic ovary syndrome. In: Azziz R, Nestler JE, Dewailly D, eds. Androgen Excess Disorders in WOmen. 2nd ed. Totowa: Human Press; 2006:259-272.

[39] Couse JF, Yates MM, Sanford R, Nyska A, Nilson JH, Korach KS. Formation of cystic ovarian follicles associated with elevated luteinizing hormone requires estrogen receptor-beta. Endocrinology. 2004;145(10):4693-4702. 15231698 10.1210/en.2004-0548

[40] Jones HM, Vernon MW, Rush ME. Systematic studies invalidate the neonatally androgenized rat as a model for polycystic ovary disease. Biol Reprod. 1987;36(5):1253-1265. 3113505 10.1095/biolreprod36.5.1253

[41] Abbott DH, Barnett DK, Levine JE, et al. Endocrine antecedents of polycystic ovary syndrome in fetal and infant prenatally androgenized female rhesus monkeys. Biol Reprod. 2008;79(1):154-163. 18385445 10.1095/biolreprod.108.067702 PMC2531213

[42] Abbott DH, Barnett DK, Bruns CM, Dumesic DA. Androgen excess fetal programming of female reproduction: a developmental aetiology for polycystic ovary syndrome? Hum Reprod Update. 2005;11(4):357-374. 15941725 10.1093/humupd/dmi013

[43] Dumesic DA, Abbott DH, Eisner JR, Goy RW. Prenatal exposure of female rhesus monkeys to testosterone propionate increases serum luteinizing hormone levels in adulthood. Fertil Steril. 1997;67(1):155-163. 8986701 10.1016/s0015-0282(97)81873-0

[44] Goy RW, Bercovitch FB, McBrair MC. Behavioral masculinization is independent of genital masculinization in prenatally androgenized female rhesus macaques. Horm Behav. 1988;22(4):552-571. 3235069 10.1016/0018-506x(88)90058-x

[45] Goy RW, Robinson JA. Prenatal exposure of rhesus monkeys to patent androgens: morphological, behavioral, and physiological conse- quences. In: Hunt VR, Smith MK, Worth D, eds. Banbury Report 11: Environmental Factors in Human Growth and Development. New York: Cold Spring Harbor Laboratory, Cold Spring Harbor; 1982:355-378.

[46] Sarma HN, Manikkam M, Herkimer C, et al. Fetal programming: excess prenatal testosterone reduces postnatal luteinizing hormone, but not follicle-stimulating hormone responsiveness, to estradiol negative feedback in the female. Endocrinology. 2005;146(10):4281-4291. 15976056 10.1210/en.2005-0322

[47] Veiga-Lopez A, Astapova OI, Aizenberg EF, Lee JS, Padmanabhan V. Developmental programming: contribution of prenatal androgen and estrogen to estradiol feedback systems and periovulatory hormonal dynamics in sheep. Biol Reprod. 2009;80(4):718-725. 19122183 10.1095/biolreprod.108.074781 PMC2804826

[48] Veiga-Lopez A, Ye W, Phillips DJ, Herkimer C, Knight PG, Padmanabhan V. Developmental programming: deficits in reproductive hormone dynamics and ovulatory outcomes in prenatal, testosterone-treated sheep. Biol Reprod. 2008;78(4):636-647. 18094354 10.1095/biolreprod.107.065904

[49] Robinson JE, Forsdike RA, Taylor JA. In utero exposure of female lambs to testosterone reduces the sensitivity of the gonadotropin-releasing hormone neuronal network to inhibition by progesterone. Endocrinology. 1999;140(12):5797-5805. 10579346 10.1210/endo.140.12.7205

[50] Unsworth WP, Taylor JA, Robinson JE. Prenatal programming of reproductive neuroendocrine function: the effect of prenatal androgens on the development of estrogen positive feedback and ovarian cycles in the ewe. Biol Reprod. 2005;72(3):619-627. 15509728 10.1095/biolreprod.104.035691

[51] Sharma TP, Herkimer C, West C, et al. Fetal programming: prenatal androgen disrupts positive feedback actions of estradiol but does not affect timing of puberty in female sheep. Biol Reprod. 2002;66(4):924-933. 11906910 10.1095/biolreprod66.4.924

[52] Cernea M, Padmanabhan V, Goodman RL, Coolen LM, Lehman MN. Prenatal testosterone treatment leads to changes in the morphology of KNDy neurons, their inputs, and projections to GnRH cells in female sheep. Endocrinology. 2015;156(9):3277-3291. 26061725 10.1210/en.2014-1609 PMC4541615

[53] Cheng G, Coolen LM, Padmanabhan V, Goodman RL, Lehman MN. The kisspeptin/neurokinin B/dynorphin (KNDy) cell population of the arcuate nucleus: sex differences and effects of prenatal testosterone in sheep. Endocrinology. 2010;151(1):301-311. 19880810 10.1210/en.2009-0541 PMC2803147

[54] Ahn T, Fergani C, Coolen LM, Padmanabhan V, Lehman MN. Prenatal testosterone excess decreases neurokinin 3 receptor immunoreactivity within the arcuate nucleus KNDy cell population. J Neuroendocrinol. 2015;27(2):100-110. 25496429 10.1111/jne.12244 PMC4412353

[55] Porter DT, Moore AM, Cobern JA, et al. Prenatal testosterone exposure alters GABAergic synaptic inputs to GnRH and KNDy neurons in a sheep model of polycystic ovarian syndrome. Endocrinology. 2019;160(11):2529-2542. 31415088 10.1210/en.2019-00137 PMC6779074

[56] Cernea M, Phillips R, Padmanabhan V, Coolen LM, Lehman MN. Prenatal testosterone exposure decreases colocalization of insulin receptors in kisspeptin/neurokinin B/dynorphin and agouti-related peptide neurons of the adult ewe. Eur J Neurosci. 2016;44(8):2557-2568. 27543746 10.1111/ejn.13373 PMC5067216

[57] Manikkam M, Thompson RC, Herkimer C, et al. Developmental programming: impact of prenatal testosterone excess on pre- and postnatal gonadotropin regulation in sheep. Biol Reprod. 2008;78(4):648-660. 18094361 10.1095/biolreprod.107.063347

[58] West C, Foster DL, Evans NP, Robinson J, Padmanabhan V. Intra-follicular activin availability is altered in prenatally-androgenized lambs. Mol Cell Endocrinol. 2001;185(1-2):51-59. 11738794 10.1016/s0303-7207(01)00632-3

[59] Smith P, Steckler TL, Veiga-Lopez A, Padmanabhan V. Developmental programming: differential effects of prenatal testosterone and dihydrotestosterone on follicular recruitment, depletion of follicular reserve, and ovarian morphology in sheep. Biol Reprod. 2009;80(4):726-736. 19092114 10.1095/biolreprod.108.072801 PMC2804827

[60] Manikkam M, Steckler TL, Welch KB, Inskeep EK, Padmanabhan V. Fetal programming: prenatal testosterone treatment leads to follicular persistence/luteal defects; partial restoration of ovarian function by cyclic progesterone treatment. Endocrinology. 2006;147(4):1997-2007. 16373416 10.1210/en.2005-1338

[61] Steckler T, Manikkam M, Inskeep EK, Padmanabhan V. Developmental programming: follicular persistence in prenatal testosterone-treated sheep is not programmed by androgenic actions of testosterone. Endocrinology. 2007;148(7):3532-3540. 17446188 10.1210/en.2007-0339

[62] Ortega HH, Salvetti NR, Padmanabhan V. Developmental programming: prenatal androgen excess disrupts ovarian steroid receptor balance. Reproduction. 2009;137(5):865-877. 19261835 10.1530/REP-08-0491 PMC3968529

[63] Veiga-Lopez A, Ye W, Padmanabhan V. Developmental programming: prenatal testosterone excess disrupts anti-Müllerian hormone expression in preantral and antral follicles. Fertil Steril. 2012;97(3):748-756. 22245531 10.1016/j.fertnstert.2011.12.028 PMC3292625

[64] Padmanabhan V, Salvetti NR, Matiller V, Ortega HH. Developmental programming: prenatal steroid excess disrupts key members of intraovarian steroidogenic pathway in sheep. Endocrinology. 2014;155(9):3649-3660. 25061847 10.1210/en.2014-1266 PMC4138569

[65] Tonellotto Dos Santos J, Escarião da Nóbrega J Jr, Serrano Mujica LK, et al. Prenatal Androgenization of Ewes as a Model of Hirsutism in Polycystic Ovary Syndrome. Endocrinology. 2018;159(12):4056-4064. 30376052 10.1210/en.2018-00781

[66] Steckler TL, Roberts EK, Doop DD, Lee TM, Padmanabhan V. Developmental programming in sheep: administration of testosterone during 60-90 days of pregnancy reduces breeding success and pregnancy outcome. Theriogenology. 2007;67(3):459-467. 17010414 10.1016/j.theriogenology.2006.08.010

[67] Abi Salloum B, Veiga-Lopez A, Abbott DH, Burant CF, Padmanabhan V. Developmental programming: exposure to testosterone excess disrupts steroidal and metabolic environment in pregnant sheep. Endocrinology. 2015;156(6):2323-2337. 25763641 10.1210/en.2014-2006 PMC4430607

[68] Beckett EM, Astapova O, Steckler TL, Veiga-Lopez A, Padmanabhan V. Developmental programing: impact of testosterone on placental differentiation. Reproduction. 2014;148(2):199-209. 24840528 10.1530/REP-14-0055 PMC4091887

[69] Kelley AS, Puttabyatappa M, Ciarelli JN, et al. Prenatal testosterone excess disrupts placental function in a sheep model of polycystic ovary syndrome. Endocrinology. 2019;160(11):2663-2672. 31436841 10.1210/en.2019-00386 PMC6804485

[70] Veiga-Lopez A, Steckler TL, Abbott DH, et al. Developmental programming: impact of excess prenatal testosterone on intrauterine fetal endocrine milieu and growth in sheep. Biol Reprod. 2011;84(1):87-96. 20739662 10.1095/biolreprod.110.086686 PMC3012564

[71] Steckler T, Wang J, Bartol FF, Roy SK, Padmanabhan V. Fetal programming: prenatal testosterone treatment causes intrauterine growth retardation, reduces ovarian reserve and increases ovarian follicular recruitment. Endocrinology. 2005;146(7):3185-3193. 15802500 10.1210/en.2004-1444

[72] Padmanabhan V, Veiga-Lopez A, Abbott DH, Recabarren SE, Herkimer C. Developmental programming: impact of prenatal testosterone excess and postnatal weight gain on insulin sensitivity index and transfer of traits to offspring of overweight females. Endocrinology. 2010;151(2):595-605. 19966179 10.1210/en.2009-1015 PMC2817622

[73] Veiga-Lopez A, Moeller J, Patel D, et al. Developmental programming: impact of prenatal testosterone excess on insulin sensitivity, adiposity, and free fatty acid profile in postpubertal female sheep. Endocrinology. 2013;154(5):1731-1742. 23525243 10.1210/en.2012-2145 PMC4016698

[74] Puttabyatappa M, Andriessen V, Mesquitta M, Zeng L, Pennathur S, Padmanabhan V. Developmental programming: impact of gestational steroid and metabolic milieus on mediators of insulin sensitivity in prenatal testosterone-treated female sheep. Endocrinology. 2017;158(9):2783-2798. 28911168 10.1210/en.2017-00460 PMC5659659

[75] Veiga-lopez A, Muralidharan M, Padmanabhan V. 2010 Developmental programming: gestational T excess compromises fetal pancreatic differentiation. 92nd Annual Meeting of the Endocrine Society; 2010; San Diego, CA, USA.

[76] Manikkam M, Crespi EJ, Doop DD, et al. Fetal programming: prenatal testosterone excess leads to fetal growth retardation and postnatal catch-up growth in sheep. Endocrinology. 2004;145(2):790-798. 14576190 10.1210/en.2003-0478

[77] King AJ, Olivier NB, Mohankumar PS, Lee JS, Padmanabhan V, Fink GD. Hypertension caused by prenatal testosterone excess in female sheep. Am J Physiol Endocrinol Metab. 2007;292(6):E1837-E1841. 17327368 10.1152/ajpendo.00668.2006

[78] Vyas AK, Hoang V, Padmanabhan V, Gilbreath E, Mietelka KA. Prenatal programming: adverse cardiac programming by gestational testosterone excess. Sci Rep. 2016;6:28335. 27328820 10.1038/srep28335 PMC4916456

[79] Roberts EK, Padmanabhan V, Lee TM. Differential effects of prenatal testosterone timing and duration on phenotypic and behavioral masculinization and defeminization of female sheep. Biol Reprod. 2008;79(1):43-50. 18385446 10.1095/biolreprod.107.067074

[80] Jackson LM, Mytinger A, Roberts EK, et al. Developmental programming: postnatal steroids complete prenatal steroid actions to differentially organize the GnRH surge mechanism and reproductive behavior in female sheep. Endocrinology. 2013;154(4):1612-1623. 23417422 10.1210/en.2012-1613 PMC3602628

[81] Birch RA, Padmanabhan V, Foster DL, Unsworth WP, Robinson JE. Prenatal programming of reproductive neuroendocrine function: fetal androgen exposure produces progressive disruption of reproductive cycles in sheep. Endocrinology. 2003;144(4):1426-1434. 12639926 10.1210/en.2002-220965

[82] Cardoso RC, Burns A, Moeller J, Skinner DC, Padmanabhan V. Developmental programming: insulin sensitizer prevents the GnRH-stimulated LH hypersecretion in a sheep model of PCOS. Endocrinology. 2016;157(12):4641-4653. 27792406 10.1210/en.2016-1613 PMC5133353

[83] Comim FV, Hardy K, Robinson J, Franks S. Disorders of follicle development and steroidogenesis in ovaries of androgenised foetal sheep. J Endocrinol. 2015;225(1):39-46. 25792297 10.1530/JOE-14-0150

[84] Lee JS, Savabieafsahani M, Herkimer C, Sharma T, Foster DL, Padmanabhan V. Fetal programming: prenatal exposure to testosterone disrupts FSH dynamics during the sheep estrous cycle. Biol Reprod. 2003;68:366-367.

[85] Savabieasfahani M, Lee JS, Herkimer C, Sharma TP, Foster DL, Padmanabhan V. Fetal programming: testosterone exposure of the female sheep during midgestation disrupts the dynamics of its adult gonadotropin secretion during the periovulatory period. Biol Reprod. 2005;72(1):221-229. 15355876 10.1095/biolreprod.104.031070

[86] Sathishkumar K, Elkins R, Chinnathambi V, Gao HJ, Hankins GDV, Yallampalli C. Prenatal testosterone-induced fetal growth restriction is associated with down-regulation of rat placental amino acid transport. Reprod Biol Endocrin. 2011;9:110. 10.1186/1477-7827-9-110 PMC3162507 21812961

[87] Hu M, Richard JE, Maliqueo M, et al. Maternal testosterone exposure increases anxiety-like behavior and impacts the limbic system in the offspring. Proc Natl Acad Sci U S A. 2015;112(46): 14348-14353. 26578781 10.1073/pnas.1507514112 PMC4655563

[88] Sathishkumar K, Elkins R, Yallampalli U, Balakrishnan M, Yallampalli C. Fetal programming of adult hypertension in female rat offspring exposed to androgens in utero. Early Hum Dev. 2011;87(6):407-414. 21450421 10.1016/j.earlhumdev.2011.03.001 PMC3093104

[89] Chinnathambi V, Balakrishnan M, Yallampalli C, Sathishkumar K. Prenatal testosterone exposure leads to hypertension that is gonadal hormone-dependent in adult rat male and female offspring. Biol Reprod. 2012;86:137, 131-137. 22302690 10.1095/biolreprod.111.097550 PMC3364920

[90] More AS, Mishra J, Hankins GD, Yallampalli C, Sathishkumar K. Enalapril normalizes endothelium-derived hyperpolarizing factor-mediated relaxation in mesenteric artery of adult hypertensive rats prenatally exposed to testosterone. Biol Reprod. 2015;92(6):155. 25972013 10.1095/biolreprod.115.130468 PMC4652613

[91] Osuka S, Iwase A, Nakahara T, et al. Kisspeptin in the hypothalamus of 2 rat models of polycystic ovary syndrome. Endocrinology. 2017;158(2):367-377. 27983870 10.1210/en.2016-1333

[92] Dean A, Smith LB, Macpherson S, Sharpe RM. The effect of dihydrotestosterone exposure during or prior to the masculinization programming window on reproductive development in male and female rats. Int J Androl. 2012;35(3):330-339. 22248293 10.1111/j.1365-2605.2011.01236.x

[93] Sullivan SD, Moenter SM. Prenatal androgens alter GABAergic drive to gonadotropin-releasing hormone neurons: implications for a common fertility disorder. Proc Natl Acad Sci U S A. 2004;101(18):7129-7134. 15096602 10.1073/pnas.0308058101 PMC406477

[94] Moore AM, Prescott M, Campbell RE. Estradiol negative and positive feedback in a prenatal androgen-induced mouse model of polycystic ovarian syndrome. Endocrinology. 2013;154(2):796-806. 23254197 10.1210/en.2012-1954

[95] Moore AM, Prescott M, Marshall CJ, Yip SH, Campbell RE. Enhancement of a robust arcuate GABAergic input to gonadotropin-releasing hormone neurons in a model of polycystic ovarian syndrome. Proc Natl Acad Sci U S A. 2015;112(2):596-601. 25550522 10.1073/pnas.1415038112 PMC4299257

[96] Caldwell AS, Middleton LJ, Jimenez M, et al. Characterization of reproductive, metabolic, and endocrine features of polycystic ovary syndrome in female hyperandrogenic mouse models. Endocrinology. 2014;155(8):3146-3159. 24877633 10.1210/en.2014-1196

[97] Silva MS, Prescott M, Campbell RE. Ontogeny and reversal of brain circuit abnormalities in a preclinical model of PCOS. JCI Insight. 2018;3(7):e99405. 10.1172/jci.insight.99405 PMC5928858 29618656

[98] Silva MSB, Desroziers E, Hessler S, et al. Activation of arcuate nucleus GABA neurons promotes luteinizing hormone secretion and reproductive dysfunction: Implications for polycystic ovary syndrome. Ebiomedicine. 2019;44:582-596. 31178425 10.1016/j.ebiom.2019.05.065 PMC6606966

[99] Fornes R, Maliqueo M, Hu M, et al. The effect of androgen excess on maternal metabolism, placental function and fetal growth in obese dams. Sci Rep. 2017;7(1):8066. 28808352 10.1038/s41598-017-08559-w PMC5556034

[100] Fornes R, Manti M, Qi X, et al. Mice exposed to maternal androgen excess and diet-induced obesity have altered phosphorylation of catechol-O-methyltransferase in the placenta and fetal liver. Int J Obes (Lond). 2019;43(11):2176-2188. 30670847 10.1038/s41366-018-0314-8

[101] Roland AV, Nunemaker CS, Keller SR, Moenter SM. Prenatal androgen exposure programs metabolic dysfunction in female mice. J Endocrinol. 2010;207(2):213-223. 20713501 10.1677/JOE-10-0217 PMC3612271

[102] Manti M, Fornes R, Pironti G, et al Maternal androgen excess induces cardiac hypertrophy and left ventricular dysfunction in female mice offspring. Cardiovasc Res. 2020;116(3):619-632. 31382275 10.1093/cvr/cvz180

[103] Manti M, Fornes R, Qi X, et al. Maternal androgen excess and obesity induce sexually dimorphic anxiety-like behavior in the offspring. Faseb J. 2018;32(8):4158-4171. 29565738 10.1096/fj.201701263RR

[104] Witham EA, Meadows JD, Shojaei S, Kauffman AS, Mellon PL. Prenatal exposure to low levels of androgen accelerates female puberty onset and reproductive senescence in mice. Endocrinology. 2012;153(9):4522-4532. 22778229 10.1210/en.2012-1283 PMC3423623

[105] Dulka EA, Moenter SM. Prepubertal development of gonadotropin-releasing hormone neuron activity is altered by sex, age, and prenatal androgen exposure. Endocrinology. 2017;158(11):3943-3953. 28938422 10.1210/en.2017-00768 PMC5695838

[106] Barbotin AL, Peigné M, Malone SA, Giacobini P. Emerging roles of anti-müllerian hormone in hypothalamic-pituitary function. Neuroendocrinology. 2019;109(3):218-229. 31280262 10.1159/000500689 PMC6878735

[107] Tata B, Mimouni NEH, Barbotin AL, et al. Elevated prenatal anti-Müllerian hormone reprograms the fetus and induces polycystic ovary syndrome in adulthood. Nat Med. 2018;24(6):834-846. 29760445 10.1038/s41591-018-0035-5 PMC6098696

[108] Treloar OL, Wolf RC, Meyer RK. Failure of a single neonatal dose of testosterone to alter ovarian function in the Rhesus monkey. Endocrinology. 1972;90(1):281-284. 4621539 10.1210/endo-90-1-281

[109] Abbott DH . The effects of neonatal exposure to testosterone on the development of behavior in female marmoset monkeys. Ciba Found Symp. 1979;62:299-327. 10.1002/9780470720448.ch14 111908

[110] Bishop CV, Mishler EC, Takahashi DL, et al. Chronic hyperandrogenemia in the presence and absence of a western-style diet impairs ovarian and uterine structure/function in young adult rhesus monkeys. Hum Reprod. 2018;33(1):128-139. 29190387 10.1093/humrep/dex338 PMC5850861

[111] McGee WK, Bishop CV, Bahar A, et al. Elevated androgens during puberty in female rhesus monkeys lead to increased neuronal drive to the reproductive axis: a possible component of polycystic ovary syndrome. Hum Reprod. 2012;27(2):531-540. 22114112 10.1093/humrep/der393 PMC3258033

[112] McGee WK, Bishop CV, Pohl CR, et al. Effects of hyperandrogenemia and increased adiposity on reproductive and metabolic parameters in young adult female monkeys. Am J Physiol Endocrinol Metab. 2014;306(11):E1292-E1304. 24735887 10.1152/ajpendo.00310.2013 PMC4042098

[113] True C, Abbott DH, Roberts CT Jr, Varlamov O. Sex differences in androgen regulation of metabolism in nonhuman primates. Adv Exp Med Biol. 2017;1043:559-574. 29224110 10.1007/978-3-319-70178-3_24 PMC5893331

[114] Benrick A, Maliqueo M, Johansson J, et al. Enhanced insulin sensitivity and acute regulation of metabolic genes and signaling pathways after a single electrical or manual acupuncture session in female insulin-resistant rats. Acta Diabetol. 2014;51(6):963-972. 25218925 10.1007/s00592-014-0645-4

[115] Mannerås L, Cajander S, Holmäng A, et al. A new rat model exhibiting both ovarian and metabolic characteristics of polycystic ovary syndrome. Endocrinology. 2007;148(8):3781-3791. 17495003 10.1210/en.2007-0168

[116] Ressler IB, Grayson BE, Ulrich-Lai YM, Seeley RJ. Diet-induced obesity exacerbates metabolic and behavioral effects of polycystic ovary syndrome in a rodent model. Am J Physiol Endocrinol Metab. 2015;308(12):E1076-E1084. 26078189 10.1152/ajpendo.00182.2014 PMC4469809

[117] Johansson J, Feng Y, Shao R, Lönn M, Billig H, Stener-Victorin E. Intense electroacupuncture normalizes insulin sensitivity, increases muscle GLUT4 content, and improves lipid profile in a rat model of polycystic ovary syndrome. Am J Physiol Endocrinol Metab. 2010;299(4):E551-E559. 20663984 10.1152/ajpendo.00323.2010

[118] Yanes LL, Romero DG, Moulana M, et al. Cardiovascular-renal and metabolic characterization of a rat model of polycystic ovary syndrome. Gend Med. 2011;8(2):103-115. 21536229 10.1016/j.genm.2010.11.013 PMC3093922

[119] Maliqueo M, Benrick A, Alvi A, et al. Circulating gonadotropins and ovarian adiponectin system are modulated by acupuncture independently of sex steroid or β-adrenergic action in a female hyperandrogenic rat model of polycystic ovary syndrome. Mol Cell Endocrinol. 2015;412:159-169. 25963796 10.1016/j.mce.2015.04.026

[120] Maliqueo M, Sun M, Johansson J, et al. Continuous administration of a P450 aromatase inhibitor induces polycystic ovary syndrome with a metabolic and endocrine phenotype in female rats at adult age. Endocrinology. 2013;154(1):434-445. 23183180 10.1210/en.2012-1693

[121] Lang Q, Yidong X, Xueguang Z, Sixian W, Wenming X, Tao Z. ETA-mediated anti-TNF-α therapy ameliorates the phenotype of PCOS model induced by letrozole. PLoS One. 2019;14(6):e0217495. 31170164 10.1371/journal.pone.0217495 PMC6553850

[122] van Houten EL, Kramer P, McLuskey A, Karels B, Themmen AP, Visser JA. Reproductive and metabolic phenotype of a mouse model of PCOS. Endocrinology. 2012;153(6):2861-2869. 22334715 10.1210/en.2011-1754

[123] Marcondes RR, Maliqueo M, Fornes R, et al. Exercise differentially affects metabolic functions and white adipose tissue in female letrozole- and dihydrotestosterone-induced mouse models of polycystic ovary syndrome. Mol Cell Endocrinol. 2017;448:66-76. 28344042 10.1016/j.mce.2017.03.025

[124] Caldwell ASL, Edwards MC, Desai R, et al. Neuroendocrine androgen action is a key extraovarian mediator in the development of polycystic ovary syndrome. Proc Natl Acad Sci U S A. 2017;114(16):E3334-E3343. 28320971 10.1073/pnas.1616467114 PMC5402450

[125] Bertoldo MJ, Caldwell ASL, Riepsamen AH, et al. A hyperandrogenic environment causes intrinsic defects that are detrimental to follicular dynamics in a PCOS mouse model. Endocrinology. 2019;160(3):699-715. 30657917 10.1210/en.2018-00966

[126] Wang Z, Feng M, Awe O, et al Gonadotrope androgen receptor mediates pituitary responsiveness to hormones and androgen-induced subfertility. JCI Insight. 2019;5(17):e127817. 10.1172/jci.insight.127817 PMC6777920 31393859

[127] Andrisse S, Billings K, Xue P, Wu S. Insulin signaling displayed a differential tissue-specific response to low-dose dihydrotestosterone in female mice. Am J Physiol Endocrinol Metab. 2018;314(4):E353-E365. 29351485 10.1152/ajpendo.00195.2017 PMC5966754

[128] Andrisse S, Childress S, Ma Y, et al. Low-dose dihydrotestosterone drives metabolic dysfunction via cytosolic and nuclear hepatic androgen receptor mechanisms. Endocrinology. 2017;158(3):531-544. 27967242 10.1210/en.2016-1553 PMC5460775

[129] Xue P, Wang Z, Fu X, et al A hyperandrogenic mouse model to study polycystic ovary syndrome. J Vis Exp. 2018;(140):58379. 10.3791/58379 PMC6235414 30346398

[130] Navarro G, Allard C, Morford JJ, et al Androgen excess in pancreatic beta cells and neurons predisposes female mice to type 2 diabetes. JCI Insight. 2018;3(12):e98607. 10.1172/jci.insight.98607 PMC6124401 29925687

[131] Qi X, Yun C, Sun L, et al. Gut microbiota-bile acid-interleukin-22 axis orchestrates polycystic ovary syndrome. Nat Med. 2019;25(8):1225-1233. 31332392 10.1038/s41591-019-0509-0 PMC7376369

[132] Kauffman AS, Thackray VG, Ryan GE, et al. A novel letrozole model recapitulates both the reproductive and metabolic phenotypes of polycystic ovary syndrome in female mice. Biol Reprod. 2015;93(3):69. 26203175 10.1095/biolreprod.115.131631 PMC4710190

[133] Esparza LA, Schafer D, Ho BS, Thackray VG, Kauffman AS. Hyperactive LH pulses and elevated kisspeptin and NKB gene expression in the arcuate nucleus of a PCOS mouse model. Endocrinology. 2020;161(4):bqaa018. doi:10.1210/endocr/bqaa018 32031594 10.1210/endocr/bqaa018 PMC7341557

[134] Ryan GE, Malik S, Mellon PL. Antiandrogen treatment ameliorates reproductive and metabolic phenotypes in the letrozole-induced mouse model of PCOS. Endocrinology. 2018;159(4):1734-1747. 29471436 10.1210/en.2017-03218 PMC6097580

[135] Skarra DV, Hernández-Carretero A, Rivera AJ, Anvar AR, Thackray VG. Hyperandrogenemia induced by letrozole treatment of pubertal female mice results in hyperinsulinemia prior to weight gain and insulin resistance. Endocrinology. 2017;158(9):2988-3003. 28911175 10.1210/en.2016-1898 PMC5659661

[136] Torres PJ, Skarra DV, Ho BS, et al. Letrozole treatment of adult female mice results in a similar reproductive phenotype but distinct changes in metabolism and the gut microbiome compared to pubertal mice. BMC Microbiol. 2019;19(1):57. 30871463 10.1186/s12866-019-1425-7 PMC6419356

[137] Dissen GA, Garcia-Rudaz C, Paredes A, Mayer C, Mayerhofer A, Ojeda SR. Excessive ovarian production of nerve growth factor facilitates development of cystic ovarian morphology in mice and is a feature of polycystic ovarian syndrome in humans. Endocrinology. 2009;150(6):2906-2914. 19264868 10.1210/en.2008-1575 PMC2689806

[138] Wilson JL, Chen W, Dissen GA, et al. Excess of nerve growth factor in the ovary causes a polycystic ovary-like syndrome in mice, which closely resembles both reproductive and metabolic aspects of the human syndrome. Endocrinology. 2014;155(11):4494-4506. 25211588 10.1210/en.2014-1368 PMC4197978

[139] Matzuk MM, DeMayo FJ, Hadsell LA, Kumar TR. Overexpression of human chorionic gonadotropin causes multiple reproductive defects in transgenic mice. Biol Reprod. 2003;69(1):338-346. 12672665 10.1095/biolreprod.102.013953

[140] Hill JW, Elias CF, Fukuda M, et al. Direct insulin and leptin action on pro-opiomelanocortin neurons is required for normal glucose homeostasis and fertility. Cell Metab. 2010;11(4):286-297. 20374961 10.1016/j.cmet.2010.03.002 PMC2854520

[141] Marino JS, Iler J, Dowling AR, et al. Adipocyte dysfunction in a mouse model of polycystic ovary syndrome (PCOS): evidence of adipocyte hypertrophy and tissue-specific inflammation. PLoS One. 2012;7(10):e48643. 23119079 10.1371/journal.pone.0048643 PMC3485364

[142] Shi D, Dyck MK, Uwiera RR, Russell JC, Proctor SD, Vine DF. A unique rodent model of cardiometabolic risk associated with the metabolic syndrome and polycystic ovary syndrome. Endocrinology. 2009;150(9):4425-4436. 19470707 10.1210/en.2008-1612

[143] Azziz R . PCOS: animal models for PCOS - not the real thing. Nat Rev Endocrinol. 2017;13(7):382-384. 28474686 10.1038/nrendo.2017.57

[144] Arifin E, Shively CA, Register TC, Cline JM. Polycystic ovary syndrome with endometrial hyperplasia in a cynomolgus monkey (*Macaca fascicularis*). Vet Pathol. 2008;45(4):512-515. 18587099 10.1354/vp.45-4-512

[145] Teede HJ, Misso ML, Costello MF, et al.; International PCOS Network. Recommendations from the international evidence-based guideline for the assessment and management of polycystic ovary syndrome. Hum Reprod. 2018;33(9):1602-1618. 30052961 10.1093/humrep/dey256 PMC6112576

[146] Polson DW, Adams J, Wadsworth J, Franks S. Polycystic ovaries–a common finding in normal women. Lancet. 1988;1(8590):870-872. 2895373 10.1016/s0140-6736(88)91612-1

[147] Johnstone EB, Rosen MP, Neril R, et al. The polycystic ovary post-rotterdam: a common, age-dependent finding in ovulatory women without metabolic significance. J Clin Endocrinol Metab. 2010;95(11):4965-4972. 20719841 10.1210/jc.2010-0202 PMC2968725

[148] Azziz R, Fox LM, Zacur HA, Parker CR Jr, Boots LR. Adrenocortical secretion of dehydroepiandrosterone in healthy women: highly variable response to adrenocorticotropin. J Clin Endocrinol Metab. 2001;86(6):2513-2517. 11397848 10.1210/jcem.86.6.7587

[149] Moran C, Reyna R, Boots LS, Azziz R. Adrenocortical hyperresponsiveness to corticotropin in polycystic ovary syndrome patients with adrenal androgen excess. Fertil Steril. 2004;81(1):126-131. 14711555 10.1016/j.fertnstert.2003.07.008

[150] Rosenfield RL . Ovarian and adrenal function in polycystic ovary syndrome. Endocrinol Metab Clin North Am. 1999;28(2):265-293. 10352919 10.1016/s0889-8529(05)70070-0

[151] Gilling-Smith C, Story H, Rogers V, Franks S. Evidence for a primary abnormality of thecal cell steroidogenesis in the polycystic ovary syndrome. Clin Endocrinol (Oxf). 1997;47(1):93-99. 9302378 10.1046/j.1365-2265.1997.2321049.x

[152] Nelson VL, Legro RS, Strauss JF 3rd, McAllister JM. Augmented androgen production is a stable steroidogenic phenotype of propagated theca cells from polycystic ovaries. Mol Endocrinol. 1999;13(6):946-957. 10379893 10.1210/mend.13.6.0311

[153] Nisenblat V, Norman RJ. Androgens and polycystic ovary syndrome. Curr Opin Endocrinol Diabetes Obes. 2009;16(3):224-231. 19390322 10.1097/MED.0b013e32832afd4d

[154] Taylor AE, McCourt B, Martin KA, et al. Determinants of abnormal gonadotropin secretion in clinically defined women with polycystic ovary syndrome. J Clin Endocrinol Metab. 1997;82(7):2248-2256. 9215302 10.1210/jcem.82.7.4105

[155] Chang PL, Lindheim SR, Lowre C, et al. Normal ovulatory women with polycystic ovaries have hyperandrogenic pituitary-ovarian responses to gonadotropin-releasing hormone-agonist testing. J Clin Endocrinol Metab. 2000;85(3):995-1000. 10720029 10.1210/jcem.85.3.6452

[156] Abe S, Suzuki T, Ito T, et al Differential expression of GABA(A) receptor subunit mRNAs and ligand binding sites in rat brain following phencyclidine administration. Synapse. 2000;38:51-60. 10941140 10.1002/1098-2396(200010)38:1<51::AID-SYN6>3.0.CO;2-A

[157] Mortensen M, Ehrmann DA, Littlejohn E, Rosenfield RL. Asymptomatic volunteers with a polycystic ovary are a functionally distinct but heterogeneous population. J Clin Endocrinol Metab. 2009;94(5):1579-1586. 19240158 10.1210/jc.2008-2771 PMC2684482

[158] Berg T, Silveira MA, Moenter SM. Prepubertal development of GABAergic transmission to gonadotropin-releasing hormone (GnRH) neurons and postsynaptic response are altered by prenatal androgenization. J Neurosci. 2018;38(9):2283-2293. 29374136 10.1523/JNEUROSCI.2304-17.2018 PMC5830516

[159] Terasawa E . Mechanism of pulsatile GnRH release in primates: unresolved questions. Mol Cell Endocrinol. 2019;498:110578. 31518609 10.1016/j.mce.2019.110578 PMC6944307

[160] Clarkson J, Han SY, Piet R, et al. Definition of the hypothalamic GnRH pulse generator in mice. Proc Natl Acad Sci U S A. 2017;114(47):E10216-E10223. 29109258 10.1073/pnas.1713897114 PMC5703322

[161] Caldwell AS, Eid S, Kay CR, et al. Haplosufficient genomic androgen receptor signaling is adequate to protect female mice from induction of polycystic ovary syndrome features by prenatal hyperandrogenization. Endocrinology. 2015;156(4):1441-1452. 25643156 10.1210/en.2014-1887

[162] Brown RE, Wilkinson DA, Imran SA, Caraty A, Wilkinson M. Hypothalamic kiss1 mRNA and kisspeptin immunoreactivity are reduced in a rat model of polycystic ovary syndrome (PCOS). Brain Res. 2012;1467:1-9. 22668987 10.1016/j.brainres.2012.05.049

[163] Cimino I, Casoni F, Liu X, et al. Novel role for anti-Müllerian hormone in the regulation of GnRH neuron excitability and hormone secretion. Nat Commun. 2016;7:10055. 26753790 10.1038/ncomms10055 PMC4729924

[164] Baillargeon JP, Nestler JE. Commentary: polycystic ovary syndrome: a syndrome of ovarian hypersensitivity to insulin? J Clin Endocrinol Metab. 2006;91(1):22-24. 16263814 10.1210/jc.2005-1804 PMC3846532

[165] Nestler JE, Powers LP, Matt DW, et al. A direct effect of hyperinsulinemia on serum sex hormone-binding globulin levels in obese women with the polycystic ovary syndrome. J Clin Endocrinol Metab. 1991;72(1):83-89. 1898744 10.1210/jcem-72-1-83

[166] Franks S, Mason H, Willis D. Follicular dynamics in the polycystic ovary syndrome. Mol Cell Endocrinol. 2000;163(1-2):49-52. 10963873 10.1016/s0303-7207(99)00239-7

[167] Dumesic DA, Abbott DH. Accounting for the follicle population in the polycystic ovary. In: Dunaif ACR, Franks S, Legro RS, eds. Polycystic Ovary Syndrome;Current Controversies, from the Ovary to the Pancreas. Totowa, NJ: Humana Press Inc.; 2008:9-24.

[168] Dumesic DA, Schramm RD, Peterson E, Paprocki AM, Zhou R, Abbott DH. Impaired developmental competence of oocytes in adult prenatally androgenized female rhesus monkeys undergoing gonadotropin stimulation for in vitro fertilization. J Clin Endocrinol Metab. 2002;87(3):1111-1119. 11889174 10.1210/jcem.87.3.8287

[169] Glueck CJ, Goldenberg N. Characteristics of obesity in polycystic ovary syndrome: etiology, treatment, and genetics. Metabolism. 2019;92:108-120. 30445140 10.1016/j.metabol.2018.11.002

[170] Poretsky L, Cataldo NA, Rosenwaks Z, Giudice LC. The insulin-related ovarian regulatory system in health and disease. Endocr Rev. 1999;20(4):535-582. 10453357 10.1210/edrv.20.4.0374

[171] Diamanti-Kandarakis E, Argyrakopoulou G, Economou F, Kandaraki E, Koutsilieris M. Defects in insulin signaling pathways in ovarian steroidogenesis and other tissues in polycystic ovary syndrome (PCOS). J Steroid Biochem Mol Biol. 2008;109(3-5):242-246. 18440223 10.1016/j.jsbmb.2008.03.014

[172] Agarwal SK, Vogel K, Weitsman SR, Magoffin DA. Leptin antagonizes the insulin-like growth factor-I augmentation of steroidogenesis in granulosa and theca cells of the human ovary. J Clin Endocrinol Metab. 1999;84(3):1072-1076. 10084597 10.1210/jcem.84.3.5543

[173] Greisen S, Ledet T, Møller N, et al. Effects of leptin on basal and FSH stimulated steroidogenesis in human granulosa luteal cells. Acta Obstet Gynecol Scand. 2000;79(11):931-935. 11081675

[174] Cook CL, Siow Y, Brenner AG, Fallat ME. Relationship between serum müllerian-inhibiting substance and other reproductive hormones in untreated women with polycystic ovary syndrome and normal women. Fertil Steril. 2002;77(1):141-146. 11779604 10.1016/s0015-0282(01)02944-2

[175] Pigny P, Jonard S, Robert Y, Dewailly D. Serum anti-Mullerian hormone as a surrogate for antral follicle count for definition of the polycystic ovary syndrome. J Clin Endocrinol Metab. 2006;91(3):941-945. 16368745 10.1210/jc.2005-2076

[176] Teede H, Misso M, Tassone EC, et al. Anti-Müllerian hormone in PCOS: a review informing international guidelines. Trends Endocrinol Metab. 2019;30(7):467-478. 31160167 10.1016/j.tem.2019.04.006

[177] Durlinger AL, Visser JA, Themmen AP. Regulation of ovarian function: the role of anti-Müllerian hormone. Reproduction. 2002;124(5):601-609. 12416998 10.1530/rep.0.1240601

[178] van Rooij IA, Broekmans FJ, te Velde ER, et al. Serum anti-Müllerian hormone levels: a novel measure of ovarian reserve. Hum Reprod. 2002;17(12):3065-3071. 12456604 10.1093/humrep/17.12.3065

[179] Peñarrubia J, Fábregues F, Manau D, et al. Basal and stimulation day 5 anti-Mullerian hormone serum concentrations as predictors of ovarian response and pregnancy in assisted reproductive technology cycles stimulated with gonadotropin-releasing hormone agonist–gonadotropin treatment. Hum Reprod. 2005;20(4):915-922. 15665015 10.1093/humrep/deh718

[180] Dewailly D, Andersen CY, Balen A, et al The physiology and clinical utility of anti-Mullerian hormone in women. Hum Reprod Update. 2014;20(3):370-385. 24430863 10.1093/humupd/dmt062

[181] Xu J, Lawson MS, Mitalipov SM, Park BS, Xu F. Stage-specific modulation of antimüllerian hormone promotes primate follicular development and oocyte maturation in the matrix-free three-dimensional culture. Fertil Steril. 2018;110(6):1162-1172. 30396561 10.1016/j.fertnstert.2018.07.006 PMC6226025

[182] Xu J, Xu F, Lawson MS, et al. Anti-Müllerian hormone is a survival factor and promotes the growth of rhesus macaque preantral follicles during matrix-free culture. Biol Reprod. 2018;98(2):197-207. 29293939 10.1093/biolre/iox181 PMC6248587

[183] Xu J, Xu F, Letaw JH, Park BS, Searles RP, Ferguson BM. Anti-Müllerian hormone is produced heterogeneously in primate preantral follicles and is a potential biomarker for follicle growth and oocyte maturation in vitro. J Assist Reprod Genet. 2016;33(12):1665-1675. 27638727 10.1007/s10815-016-0804-3 PMC5234704

[184] Rice S, Ojha K, Whitehead S, Mason H. Stage-specific expression of androgen receptor, follicle-stimulating hormone receptor, and anti-Müllerian hormone type II receptor in single, isolated, human preantral follicles: relevance to polycystic ovaries. J Clin Endocrinol Metab. 2007;92(3):1034-1040. 17179193 10.1210/jc.2006-1697

[185] Pellatt L, Hanna L, Brincat M, et al. Granulosa cell production of anti-Müllerian hormone is increased in polycystic ovaries. J Clin Endocrinol Metab. 2007;92(1):240-245. 17062765 10.1210/jc.2006-1582

[186] Pellatt L, Rice S, Mason HD. Anti-Müllerian hormone and polycystic ovary syndrome: a mountain too high? Reproduction. 2010;139(5):825-833. 20207725 10.1530/REP-09-0415

[187] Persani L, Rossetti R, Di Pasquale E, Cacciatore C, Fabre S. The fundamental role of bone morphogenetic protein 15 in ovarian function and its involvement in female fertility disorders. Hum Reprod Update. 2014;20(6):869-883. 24980253 10.1093/humupd/dmu036

[188] Riepsamen AH, Chan K, Lien S, et al. Serum concentrations of oocyte-secreted factors BMP15 and GDF9 during IVF and in women with reproductive pathologies. Endocrinology. 2019;160(10):2298-2313. 31211369 10.1210/en.2019-00264

[189] Russell DL, Gilchrist RB, Brown HM, Thompson JG. Bidirectional communication between cumulus cells and the oocyte: old hands and new players? Theriogenology. 2016;86(1):62-68. 27160446 10.1016/j.theriogenology.2016.04.019

[190] Liu Q, Li Y, Feng Y, et al. Single-cell analysis of differences in transcriptomic profiles of oocytes and cumulus cells at GV, MI, MII stages from PCOS patients. Sci Rep. 2016;6:39638. 28004769 10.1038/srep39638 PMC5177934

[191] Peluso C, Fonseca FL, Gastaldo GG, et al. AMH and AMHR2 polymorphisms and AMH serum level can predict assisted reproduction outcomes: a cross-sectional study. Cell Physiol Biochem. 2015;35(4):1401-1412. 25790842 10.1159/000373961

[192] Wood JR, Dumesic DA, Abbott DH, Strauss JF 3rd. Molecular abnormalities in oocytes from women with polycystic ovary syndrome revealed by microarray analysis. J Clin Endocrinol Metab. 2007;92(2):705-713. 17148555 10.1210/jc.2006-2123

[193] Coyle C, Campbell RE. Pathological pulses in PCOS. Mol Cell Endocrinol. 2019;498:110561. 31461666 10.1016/j.mce.2019.110561

[194] Pastor CL, Griffin-Korf ML, Aloi JA, Evans WS, Marshall JC. Polycystic ovary syndrome: evidence for reduced sensitivity of the gonadotropin-releasing hormone pulse generator to inhibition by estradiol and progesterone. J Clin Endocrinol Metab. 1998;83(2):582-590. 9467578 10.1210/jcem.83.2.4604

[195] Chhabra S, McCartney CR, Yoo RY, Eagleson CA, Chang RJ, Marshall JC. Progesterone inhibition of the hypothalamic gonadotropin-releasing hormone pulse generator: evidence for varied effects in hyperandrogenemic adolescent girls. J Clin Endocrinol Metab. 2005;90(5):2810-2815. 15728200 10.1210/jc.2004-2359

[196] Ruddenklau A, Campbell RE. Neuroendocrine impairments of polycystic ovary syndrome. Endocrinology. 2019;160(10):2230-2242. 31265059 10.1210/en.2019-00428

[197] Malone SA, Papadakis GE, Messina A, et al Defective AMH signaling disrupts GnRH neuron development and function and contributes to hypogonadotropic hypogonadism. Elife. 2019;8:e47198. 31291191 10.7554/eLife.47198 PMC6620045

[198] Day F, Karaderi T, Jones MR, et al.; 23andMe Research Team. Large-scale genome-wide meta-analysis of polycystic ovary syndrome suggests shared genetic architecture for different diagnosis criteria. PLoS Genet. 2018;14(12):e1007813. 30566500 10.1371/journal.pgen.1007813 PMC6300389

[199] Chen ZJ, Zhao H, He L, et al. Genome-wide association study identifies susceptibility loci for polycystic ovary syndrome on chromosome 2p16.3, 2p21 and 9q33.3. Nat Genet. 2011;43(1):55-59. 21151128 10.1038/ng.732

[200] Liu H, Zhao H, Chen ZJ. Genome-wide association studies for polycystic ovary syndrome. Semin Reprod Med. 2016;34(4):224-229. 27513023 10.1055/s-0036-1585403

[201] Jones MR, Chua AK, Mengesha EA, et al.; Reproductive Medicine Network. Metabolic and cardiovascular genes in polycystic ovary syndrome: a candidate-wide association study (CWAS). Steroids. 2012;77(4):317-322. 22178785 10.1016/j.steroids.2011.12.005 PMC3689580

[202] Hayes MG, Urbanek M, Ehrmann DA, et al.; Reproductive Medicine Network. Genome-wide association of polycystic ovary syndrome implicates alterations in gonadotropin secretion in European ancestry populations. Nat Commun. 2015;6:7502. 26284813 10.1038/ncomms8502 PMC4557132

[203] Manolio TA, Collins FS, Cox NJ, et al. Finding the missing heritability of complex diseases. Nature. 2009;461(7265):747-753. 19812666 10.1038/nature08494 PMC2831613

[204] McAllister JM, Modi B, Miller BA, et al. Overexpression of a DENND1A isoform produces a polycystic ovary syndrome theca phenotype. Proc Natl Acad Sci U S A. 2014;111(15):E1519-E1527. 24706793 10.1073/pnas.1400574111 PMC3992676

[205] Azziz R . PCOS in 2015: new insights into the genetics of polycystic ovary syndrome. Nat Rev Endocrinol. 2016;12:183. 26822926 10.1038/nrendo.2016.9

[206] Hanson MA, Gluckman PD. Early developmental conditioning of later health and disease: physiology or pathophysiology? Physiol Rev. 2014;94(4):1027-1076. 25287859 10.1152/physrev.00029.2013 PMC4187033

[207] Barker DJ . The origins of the developmental origins theory. J Intern Med. 2007;261(5):412-417. 17444880 10.1111/j.1365-2796.2007.01809.x

[208] Stener-Victorin E, Holm G, Labrie F, Nilsson L, Janson PO, Ohlsson C. Are there any sensitive and specific sex steroid markers for polycystic ovary syndrome? J Clin Endocrinol Metab. 2010;95(2):810-819. 20016048 10.1210/jc.2009-1908

[209] O’Reilly MW, Kempegowda P, Walsh M, et al. AKR1C3-mediated adipose androgen generation drives lipotoxicity in women with polycystic ovary syndrome. J Clin Endocrinol Metab. 2017;102(9):3327-3339. 28645211 10.1210/jc.2017-00947 PMC5587066

[210] Boomsma CM, Fauser BC, Macklon NS. Pregnancy complications in women with polycystic ovary syndrome. Semin Reprod Med. 2008;26(1):72-84. 18181085 10.1055/s-2007-992927

[211] Roos N, Kieler H, Sahlin L, Ekman-Ordeberg G, Falconer H, Stephansson O. Risk of adverse pregnancy outcomes in women with polycystic ovary syndrome: population based cohort study. Bmj. 2011;343:d6309. 21998337 10.1136/bmj.d6309 PMC3192872

[212] Palomba S, Marotta R, Di Cello A, et al. Pervasive developmental disorders in children of hyperandrogenic women with polycystic ovary syndrome: a longitudinal case-control study. Clin Endocrinol (Oxf). 2012;77(6):898-904. 22612600 10.1111/j.1365-2265.2012.04443.x

[213] Palomba S, Russo T, Falbo A, et al. Macroscopic and microscopic findings of the placenta in women with polycystic ovary syndrome. Hum Reprod. 2013;28(10):2838-2847. 23756703 10.1093/humrep/det250

[214] Falbo A, Rocca M, Russo T, et al. Changes in androgens and insulin sensitivity indexes throughout pregnancy in women with polycystic ovary syndrome (PCOS): relationships with adverse outcomes. J Ovarian Res. 2010;3:23. 20942923 10.1186/1757-2215-3-23 PMC2967533

[215] Maliqueo M, Sundström Poromaa I, Vanky E, et al. Placental STAT3 signaling is activated in women with polycystic ovary syndrome. Hum Reprod. 2015;30(3):692-700. 25609240 10.1093/humrep/deu351

[216] Maliqueo M, Lara HE, Sánchez F, Echiburú B, Crisosto N, Sir-Petermann T. Placental steroidogenesis in pregnant women with polycystic ovary syndrome. Eur J Obstet Gynecol Reprod Biol. 2013;166(2):151-155. 23122578 10.1016/j.ejogrb.2012.10.015

[217] Cesta CE, Oberg AS, Ibrahimson A, et al Maternal polycystic ovary syndrome and risk of neuropsychiatric disorders in offspring: prenatal androgen exposure or genetic confounding? Psychol Med. 2020;50(4):616-624. 10.1017/S0033291719000424 PMC7093321 30857571

[218] Lubahn DB, Moyer JS, Golding TS, Couse JF, Korach KS, Smithies O. Alteration of reproductive function but not prenatal sexual development after insertional disruption of the mouse estrogen receptor gene. Proc Natl Acad Sci U S A. 1993;90(23):11162-11166. 8248223 10.1073/pnas.90.23.11162 PMC47942

[219] Walters KA, Rodriguez Paris V, Aflatounian A, Handelsman DJ. Androgens and ovarian function: translation from basic discovery research to clinical impact. J Endocrinol. 2019;242(2):R23-R50. 31125975 10.1530/JOE-19-0096

[220] Dumesic DA, Akopians AL, Madrigal VK, et al. Hyperandrogenism accompanies increased intra-abdominal fat storage in normal weight polycystic ovary syndrome women. J Clin Endocrinol Metab. 2016;101(11):4178-4188. 27571186 10.1210/jc.2016-2586 PMC5095243

[221] Dumesic DA, Phan JD, Leung KL, et al. Adipose insulin resistance in normal-weight women with polycystic ovary syndrome. J Clin Endocrinol Metab. 2019;104(6):2171-2183. 30649347 10.1210/jc.2018-02086 PMC6482023

[222] Chang RJ, Dumesic DA. Polycystic ovary syndrome and hyperandrogenic states. In: Strauss JF, Barbieri R, eds. Yen and Jaffe’s Reproductive Endocrinology: Physiology, Pathophysiology and Clinical Management. 8th ed. 2018:520-555.

[223] Cardoso RC, Padmanabhan V. Developmental programming of PCOS traits: insights from the sheep. Med Sci. 2019;7(7):79. 10.3390/medsci7070079 PMC6681354 31336724

[224] Piltonen TT, Giacobini P, Edvinsson A, et al Circulating antimullerian hormone and steroid hormone levels remain high in pregnant women with polycystic ovary syndrome at term. Fertil Steril. 2019;111:588-596.e581. 30630591 10.1016/j.fertnstert.2018.11.028

[225] Benrick A, Maliqueo M, Miao S, et al. Resveratrol is not as effective as physical exercise for improving reproductive and metabolic functions in rats with dihydrotestosterone-induced polycystic ovary syndrome. Evid Based Complement Alternat Med. 2013;2013:964070. 23690868 10.1155/2013/964070 PMC3638597

[226] Mannerås L, Cajander S, Lönn M, Stener-Victorin E. Acupuncture and exercise restore adipose tissue expression of sympathetic markers and improve ovarian morphology in rats with dihydrotestosterone-induced PCOS. Am J Physiol Regul Integr Comp Physiol. 2009;296(4):R1124-R1131. 19158405 10.1152/ajpregu.90947.2008

[227] Mannerås L, Jonsdottir IH, Holmäng A, Lönn M, Stener-Victorin E. Low-frequency electro-acupuncture and physical exercise improve metabolic disturbances and modulate gene expression in adipose tissue in rats with dihydrotestosterone-induced polycystic ovary syndrome. Endocrinology. 2008;149(7):3559-3568. 18388196 10.1210/en.2008-0053

[228] Maliqueo M, Benrick A, Marcondes RR, Johansson J, Sun M, Stener-Victorin E. Acupuncture does not ameliorate metabolic disturbances in the P450 aromatase inhibitor-induced rat model of polycystic ovary syndrome. Exp Physiol. 2017;102(1):113-127. 27790765 10.1113/EP085983

[229] Naessen T, Kushnir MM, Chaika A, et al. Steroid profiles in ovarian follicular fluid in women with and without polycystic ovary syndrome, analyzed by liquid chromatography-tandem mass spectrometry. Fertil Steril. 2010;94(6):2228-2233. 20171618 10.1016/j.fertnstert.2009.12.081

[230] Xita N, Lazaros L, Georgiou I, Tsatsoulis A. CYP19 gene: a genetic modifier of polycystic ovary syndrome phenotype. Fertil Steril. 2010;94(1):250-254. 19324338 10.1016/j.fertnstert.2009.01.147

[231] Yu YY, Sun CX, Liu YK, Li Y, Wang L, Zhang W. Promoter methylation of CYP19A1 gene in Chinese polycystic ovary syndrome patients. Gynecol Obstet Invest. 2013;76(4):209-213. 24157654 10.1159/000355314

[232] Rosencrantz MA, Wachs DS, Coffler MS, Malcom PJ, Donohue M, Chang RJ. Comparison of inhibin B and estradiol responses to intravenous FSH in women with polycystic ovary syndrome and normal women. Hum Reprod. 2010;25(1):198-203. 19850592 10.1093/humrep/dep373 PMC2794669

[233] Guedikian AA, Lee AY, Grogan TR, et al. Reproductive and metabolic determinants of granulosa cell dysfunction in normal-weight women with polycystic ovary syndrome. Fertil Steril. 2018;109(3):508-515. 29428312 10.1016/j.fertnstert.2017.11.017 PMC5812340

[234] Leung A, Sakkas D, Pang S, Thornton K, Resetkova N. Assisted reproductive technology outcomes in female-to-male transgender patients compared with cisgender patients: a new frontier in reproductive medicine. Fertil Steril. 2019;112(5):858-865. 31594633 10.1016/j.fertnstert.2019.07.014

[235] Phillips KA, Bales KL, Capitanio JP, et al. Why primate models matter. Am J Primatol. 2014;76(9):801-827. 24723482 10.1002/ajp.22281 PMC4145602

[236] Francis PJ, Appukuttan B, Simmons E, et al. Rhesus monkeys and humans share common susceptibility genes for age-related macular disease. Hum Mol Genet. 2008;17(17):2673-2680. 18535016 10.1093/hmg/ddn167 PMC2733804

[237] Rogers J, Raveendran M, Fawcett GL, et al. CRHR1 genotypes, neural circuits and the diathesis for anxiety and depression. Mol Psychiatry. 2013;18(6):700-707. 23147386 10.1038/mp.2012.152 PMC3663915

[238] Oler JA, Fox AS, Shelton SE, et al. Amygdalar and hippocampal substrates of anxious temperament differ in their heritability. Nature. 2010;466(7308):864-868. 20703306 10.1038/nature09282 PMC2998538

[239] Dray BK, Raveendran M, Harris RA, et al. Mismatch repair gene mutations lead to lynch syndrome colorectal cancer in rhesus macaques. Genes Cancer. 2018;9(3-4):142-152. 30108684 10.18632/genesandcancer.170 PMC6086002

[240] Moshiri A, Chen R, Kim S, et al. A nonhuman primate model of inherited retinal disease. J Clin Invest. 2019;129(2):863-874. 30667376 10.1172/JCI123980 PMC6355306

[241] Fawcett GL, Dettmer AM, Kay D, et al. Quantitative genetics of response to novelty and other stimuli by infant rhesus macaques (Macaca mulatta) across three behavioral assessments. Int J Primatol. 2014;35(1):325-339. 24701001 10.1007/s10764-014-9750-z PMC3970820

[242] Ma Y, Andrisse S, Chen Y, et al. Androgen receptor in the ovary theca cells plays a critical role in androgen-induced reproductive dysfunction. Endocrinology. 2017;158(1):98-108. 27841936 10.1210/en.2016-1608 PMC5412974

[243] Mannerås-Holm L, Leonhardt H, Kullberg J, et al. Adipose tissue has aberrant morphology and function in PCOS: enlarged adipocytes and low serum adiponectin, but not circulating sex steroids, are strongly associated with insulin resistance. J Clin Endocrinol Metab. 2011;96(2):E304-E311. 21084397 10.1210/jc.2010-1290

[244] Benrick A, Chanclón B, Micallef P, et al. Adiponectin protects against development of metabolic disturbances in a PCOS mouse model. Proc Natl Acad Sci U S A. 2017;114(34):E7187-E7196. 28790184 10.1073/pnas.1708854114 PMC5576831

[245] Sverrisdóttir YB, Mogren T, Kataoka J, Janson PO, Stener-Victorin E. Is polycystic ovary syndrome associated with high sympathetic nerve activity and size at birth? Am J Physiol Endocrinol Metab. 2008;294(3):E576-E581. 18198350 10.1152/ajpendo.00725.2007

[246] Schlaich MP, Straznicky N, Grima M, et al. Renal denervation: a potential new treatment modality for polycystic ovary syndrome? J Hypertens. 2011;29(5):991-996. 21358414 10.1097/HJH.0b013e328344db3a

[247] Shorakae S, Abell SK, Hiam DS, et al. High-molecular-weight adiponectin is inversely associated with sympathetic activity in polycystic ovary syndrome. Fertil Steril. 2018;109(3):532-539. 29428305 10.1016/j.fertnstert.2017.11.020

[248] Lambert EA, Teede H, Sari CI, et al. Sympathetic activation and endothelial dysfunction in polycystic ovary syndrome are not explained by either obesity or insulin resistance. Clin Endocrinol (Oxf). 2015;83(6):812-819. 25926334 10.1111/cen.12803

[249] Shorakae S, Ranasinha S, Abell S, et al. Inter-related effects of insulin resistance, hyperandrogenism, sympathetic dysfunction and chronic inflammation in PCOS. Clin Endocrinol (Oxf). 2018;89(5):628-633. 29992612 10.1111/cen.13808

[250] Steiner RA, Clifton DK, Spies HG, Resko JA. Sexual differentiation and feedback control of luteinizing hormone secretion in the rhesus monkey. Biol Reprod. 1976;15(2):206-212. 786386 10.1095/biolreprod15.2.206

[251] Abbott DH, Vepraskas SH, Horton TH, Terasawa E, Levine JE. Accelerated episodic luteinizing hormone release accompanies blunted progesterone regulation in PCOS-like female rhesus monkeys (Macaca Mulatta) exposed to testosterone during early-to-mid gestation. Neuroendocrinology. 2018;107(2):133-146. 29949806 10.1159/000490570 PMC7363207

[252] Foecking EM, Levine JE. Effects of experimental hyperandrogenemia on the female rat reproductive axis: suppression of progesterone-receptor messenger RNA expression in the brain and blockade of luteinizing hormone surges. Gend Med. 2005;2(3):155-165. 16290888 10.1016/s1550-8579(05)80044-0

[253] Baird DT, Corker CS, Davidson DW, Hunter WM, Michie EA, Van Look PF. Pituitary-ovarian relationships in polycystic ovary syndrome. J Clin Endocrinol Metab. 1977;45(4):798-801. 334789 10.1210/jcem-45-4-798

[254] Puttabyatappa M, Cardoso RC, Herkimer C, Veiga-Lopez A, Padmanabhan V. Developmental programming: postnatal estradiol modulation of prenatally organized reproductive neuroendocrine function in sheep. Reproduction. 2016;152(2):139-150. 27222598 10.1530/REP-16-0065 PMC4936966

[255] Albalawi FS, Daghestani MH, Daghestani MH, Eldali A, Warsy AS. rs4889 polymorphism in KISS1 gene, its effect on polycystic ovary syndrome development and anthropometric and hormonal parameters in Saudi women. J Biomed Sci. 2018;25(1):50. 29848339 10.1186/s12929-018-0452-2 PMC5975709

[256] Umayal B, Jayakody SN, Chandrasekharan NV, Wijesundera WS, Wijeyaratne CN. Polycystic ovary syndrome (PCOS) and kisspeptin - a Sri Lankan study. J Postgrad Med. 2019;65(1):18-23. 30004037 10.4103/jpgm.JPGM_683_17 PMC6380135

[257] Katulski K, Podfigurna A, Czyzyk A, Meczekalski B, Genazzani AD. Kisspeptin and LH pulsatile temporal coupling in PCOS patients. Endocrine. 2018;61(1):149-157. 29728876 10.1007/s12020-018-1609-1 PMC5997113

[258] Jansen HT, Hershey J, Mytinger A, Foster DL, Padmanabhan V. Developmental programming: reproductive endocrinopathies in the adult female sheep after prenatal testosterone treatment are reflected in altered ontogeny of GnRH afferents. Endocrinology. 2011;152(11):4288-4297. 21933866 10.1210/en.2011-0117 PMC3199006

[259] Foecking EM, McDevitt MA, Acosta-Martínez M, Horton TH, Levine JE. Neuroendocrine consequences of androgen excess in female rodents. Horm Behav. 2008;53(5):673-692. 18374922 10.1016/j.yhbeh.2007.12.013 PMC2413177

[260] Plant TM . The neurobiological mechanism underlying hypothalamic GnRH pulse generation: the role of kisspeptin neurons in the arcuate nucleus. F1000Res. 2019;8:F1000 Faculty Rev-982. 10.12688/f1000research.18356.2 PMC6600864 31297186

[261] Marshall CJ, Desroziers E, McLennan T, Campbell RE. Defining subpopulations of arcuate nucleus GABA neurons in male, female, and prenatally androgenized female mice. Neuroendocrinology. 2017;105(2):157-169. 27710963 10.1159/000452105

[262] Campbell RE . Defining the gonadotrophin-releasing hormone neuronal network: transgenic approaches to understanding neurocircuitry. J Neuroendocrinol. 2007;19(7):561-573. 17532792 10.1111/j.1365-2826.2007.01561.x

[263] Spergel DJ, Krüth U, Hanley DF, Sprengel R, Seeburg PH. GABA- and glutamate-activated channels in green fluorescent protein-tagged gonadotropin-releasing hormone neurons in transgenic mice. J Neurosci. 1999;19(6):2037-2050. 10066257 10.1523/JNEUROSCI.19-06-02037.1999 PMC6782541

[264] Suter KJ, Song WJ, Sampson TL, et al. Genetic targeting of green fluorescent protein to gonadotropin-releasing hormone neurons: characterization of whole-cell electrophysiological properties and morphology. Endocrinology. 2000;141(1):412-419. 10614664 10.1210/endo.141.1.7279

[265] DeFazio RA, Heger S, Ojeda SR, Moenter SM. Activation of A-type gamma-aminobutyric acid receptors excites gonadotropin-releasing hormone neurons. Mol Endocrinol. 2002;16(12):2872-2891. 12456806 10.1210/me.2002-0163

[266] Herbison AE, Moenter SM. Depolarising and hyperpolarising actions of GABA(A) receptor activation on gonadotrophin-releasing hormone neurones: towards an emerging consensus. J Neuroendocrinol. 2011;23(7):557-569. 21518033 10.1111/j.1365-2826.2011.02145.x PMC3518440

[267] Constantin S, Iremonger KJ, Herbison AE. In vivo recordings of GnRH neuron firing reveal heterogeneity and dependence upon GABAA receptor signaling. J Neurosci. 2013;33(22):9394-9401. 23719807 10.1523/JNEUROSCI.0533-13.2013 PMC6618582

[268] Taylor-Burds C, Cheng P, Wray S. Chloride accumulators NKCC1 and AE2 in mouse GnRH neurons: implications for GABAA mediated excitation. PLoS One. 2015;10(6):e0131076. 26110920 10.1371/journal.pone.0131076 PMC4482508

[269] DeFazio RA, Navarro MA, Adams CE, Milescu LS, Moenter SM. Estradiol enhances the depolarizing response to GABA and AMPA synaptic conductances in arcuate kisspeptin neurons by diminishing voltage-gated potassium currents. J Neurosci. 2019;39(48):9532-9545. 31628184 10.1523/JNEUROSCI.0378-19.2019 PMC6880461

[270] Löscher W . Valproate: a reappraisal of its pharmacodynamic properties and mechanisms of action. Prog Neurobiol. 1999;58(1):31-59. 10321796 10.1016/s0301-0082(98)00075-6

[271] Kawwass JF, Sanders KM, Loucks TL, Rohan LC, Berga SL. Increased cerebrospinal fluid levels of GABA, testosterone and estradiol in women with polycystic ovary syndrome. Hum Reprod. 2017;32(7):1450-1456. 28453773 10.1093/humrep/dex086 PMC6251519

[272] Veiga-Lopez A, Moeller J, Abbott DH, Padmanabhan V. Developmental programming: rescuing disruptions in preovulatory follicle growth and steroidogenesis from prenatal testosterone disruption. J Ovarian Res. 2016;9(1):39. 27357284 10.1186/s13048-016-0250-y PMC4928247

[273] Padmanabhan V, Veiga-Lopez A, Herkimer C, et al. Developmental programming: prenatal and postnatal androgen antagonist and insulin sensitizer interventions prevent advancement of puberty and improve LH surge dynamics in prenatal testosterone-treated sheep. Endocrinology. 2015;156(7):2678-2692. 25919188 10.1210/en.2015-1235 PMC4475717

[274] De Leo V, Lanzetta D, D’Antona D, la Marca A, Morgante G. Hormonal effects of flutamide in young women with polycystic ovary syndrome. J Clin Endocrinol Metab. 1998;83(1):99-102. 9435423 10.1210/jcem.83.1.4500

[275] Sullivan SD, Moenter SM. GABAergic integration of progesterone and androgen feedback to gonadotropin-releasing hormone neurons. Biol Reprod. 2005;72(1):33-41. 15342358 10.1095/biolreprod.104.033126

[276] Pielecka J, Quaynor SD, Moenter SM. Androgens increase gonadotropin-releasing hormone neuron firing activity in females and interfere with progesterone negative feedback. Endocrinology. 2006;147(3):1474-1479. 16339200 10.1210/en.2005-1029

[277] Turgeon JL, Waring DW. Androgen modulation of luteinizing hormone secretion by female rat gonadotropes. Endocrinology. 1999;140(4):1767-1774. 10098514 10.1210/endo.140.4.6642

[278] Liberato MH, Sonohara S, Brentani MM. Effects of androgens on proliferation and progesterone-receptor levels in T47d human breast-cancer cells. Tumor Biol. 1993;14:38-45. 10.1159/000217823 8493449

[279] MacIndoe JH, Etre LA. An antiestrogenic action of androgens in human breast cancer cells. J Clin Endocrinol Metab. 1981;53(4):836-842. 7287866 10.1210/jcem-53-4-836

[280] Zhou R, Bruns CM, Bird IM, et al. Pioglitazone improves insulin action and normalizes menstrual cycles in a majority of prenatally androgenized female rhesus monkeys. Reprod Toxicol. 2007;23(3):438-448. 17306503 10.1016/j.reprotox.2006.12.009 PMC2705750

[281] Veiga-Lopez A, Lee JS, Padmanabhan V. Developmental programming: insulin sensitizer treatment improves reproductive function in prenatal testosterone-treated female sheep. Endocrinology. 2010;151(8):4007-4017. 20555028 10.1210/en.2010-0124 PMC2940534

[282] Herman RA, Wallen K. Cognitive performance in rhesus monkeys varies by sex and prenatal androgen exposure. Horm Behav. 2007;51(4):496-507. 17335823 10.1016/j.yhbeh.2007.01.005 PMC2497007

[283] Hanem LGE, Salvesen O, Juliusson PB, et al Intrauterine metformin exposure and offspring cardiometabolic risk factors (PedMet study): a 5-10 year follow-up of the PregMet randomised controlled trial. Lancet Child & Adolesc Health. 2019;3:166-174. 10.1016/S2352-4642(18)30385-7 30704873

[284] Walters KA, Edwards MC, Tesic D, et al. The role of central androgen receptor actions in regulating the hypothalamic-pituitary-ovarian axis. Neuroendocrinology. 2018;106(4):389-400. 29635226 10.1159/000487762

[285] Labrie F, Martel C, Bélanger A, Pelletier G. Androgens in women are essentially made from DHEA in each peripheral tissue according to intracrinology. J Steroid Biochem Mol Biol. 2017;168:9-18. 28153489 10.1016/j.jsbmb.2016.12.007

[286] Prough RA, Clark BJ, Klinge CM. Novel mechanisms for DHEA action. J Mol Endocrinol. 2016;56(3):R139-R155. 26908835 10.1530/JME-16-0013

[287] Vendola K, Zhou J, Wang J, Bondy CA. Androgens promote insulin-like growth factor-I and insulin-like growth factor-I receptor gene expression in the primate ovary. Hum Reprod. 1999;14(9):2328-2332. 10469704 10.1093/humrep/14.9.2328

[288] Vendola KA, Zhou J, Adesanya OO, Weil SJ, Bondy CA. Androgens stimulate early stages of follicular growth in the primate ovary. J Clin Invest. 1998;101(12):2622-2629. 9637695 10.1172/JCI2081 PMC508852

[289] Weil S, Vendola K, Zhou J, Bondy CA. Androgen and follicle-stimulating hormone interactions in primate ovarian follicle development. J Clin Endocrinol Metab. 1999;84(8):2951-2956. 10443703 10.1210/jcem.84.8.5929

[290] Weil SJ, Vendola K, Zhou J, et al. Androgen receptor gene expression in the primate ovary: cellular localization, regulation, and functional correlations. J Clin Endocrinol Metab. 1998;83(7):2479-2485. 9661631 10.1210/jcem.83.7.4917

[291] Hogg K, Young JM, Oliver EM, Souza CJ, McNeilly AS, Duncan WC. Enhanced thecal androgen production is prenatally programmed in an ovine model of polycystic ovary syndrome. Endocrinology. 2012;153(1):450-461. 22087026 10.1210/en.2011-1607

[292] Lujan ME, Kepley AL, Chizen DR, Lehotay DC, Pierson RA. Development of morphologically dominant follicles is associated with fewer metabolic disturbances in amenorrheic women with polycystic ovary syndrome: a pilot study. Ultrasound Obstet Gynecol. 2010;36(6):759-766. 20645396 10.1002/uog.7751

[293] Ortega HH, Rey F, Velazquez MM, Padmanabhan V. Developmental programming: effect of prenatal steroid excess on intraovarian components of insulin signaling pathway and related proteins in sheep. Biol Reprod. 2010;82(6):1065-1075. 20147730 10.1095/biolreprod.109.082719 PMC2874494

[294] Salvetti NR, Ortega HH, Veiga-Lopez A, Padmanabhan V. Developmental programming: impact of prenatal testosterone excess on ovarian cell proliferation and apoptotic factors in sheep. Biol Reprod. 2012;87:22, 21-10. 22539681 10.1095/biolreprod.112.100024 PMC3406557

[295] Ortega HH, Veiga-Lopez A, Sreedharan S, del Luján Velázquez MM, Salvetti NR, Padmanabhan V. Developmental programming: does prenatal steroid excess disrupt the ovarian VEGF system in sheep? Biol Reprod. 2015;93(3):58. 26178718 10.1095/biolreprod.115.131607 PMC4710184

[296] Guo X, Puttabyatappa M, Thompson RC, Padmanabhan V. Developmental programming: contribution of epigenetic enzymes to antral follicular defects in the sheep model of PCOS. Endocrinology. 2019;160(10):2471-2484. 31398247 10.1210/en.2019-00389 PMC6760338

[297] Garcia-Rudaz C, Dorfman M, Nagalla S, et al. Excessive ovarian production of nerve growth factor elicits granulosa cell apoptosis by setting in motion a tumor necrosis factor α/stathmin-mediated death signaling pathway. Reproduction. 2011;142(2):319-331. 21646391 10.1530/REP-11-0134 PMC3302175

[298] Eisner JR, Barnett MA, Dumesic DA, Abbott DH. Ovarian hyperandrogenism in adult female rhesus monkeys exposed to prenatal androgen excess. Fertil Steril. 2002;77(1):167-172. 11779609 10.1016/s0015-0282(01)02947-8

[299] Dumesic DA, Patankar MS, Barnett DK, Lesnick TG, Hutcherson BA, Abbott DH. Early prenatal androgenization results in diminished ovarian reserve in adult female rhesus monkeys. Hum Reprod. 2009;24(12):3188-3195. 19740899 10.1093/humrep/dep324 PMC2777787

[300] Zhou R, Bird IM, Dumesic DA, Abbott DH. Adrenal hyperandrogenism is induced by fetal androgen excess in a rhesus monkey model of polycystic ovary syndrome. J Clin Endocrinol Metab. 2005;90(12):6630-6637. 16174719 10.1210/jc.2005-0691 PMC1350929

[301] Luque-Ramírez M, Escobar-Morreale HF. Adrenal hyperandrogenism and polycystic ovary syndrome. Curr Pharm Des. 2016;22(36):5588-5602. 27510480 10.2174/1381612822666160720150625

[302] Baker ME, Lathe R. The promiscuous estrogen receptor: evolution of physiological estrogens and response to phytochemicals and endocrine disruptors. J Steroid Biochem Mol Biol. 2018;184:29-37. 30009950 10.1016/j.jsbmb.2018.07.001

[303] Palomba S, Daolio J, La Sala GB. Oocyte competence in women with polycystic ovary syndrome. Trends Endocrinol Metab. 2017;28(3):186-198. 27988256 10.1016/j.tem.2016.11.008

[304] Dumesic DA, Padmanabhan V, Abbott DH. Polycystic ovary syndrome and oocyte developmental competence. Obstet Gynecol Surv. 2008;63(1):39-48. 18081939 10.1097/OGX.0b013e31815e85fc PMC2655633

[305] Foong SC, Abbott DH, Zschunke MA, Lesnick TG, Phy JL, Dumesic DA. Follicle luteinization in hyperandrogenic follicles of polycystic ovary syndrome patients undergoing gonadotropin therapy for in vitro fertilization. J Clin Endocrinol Metab. 2006;91(6):2327-2333. 16551732 10.1210/jc.2005-2142

[306] Phy JL, Conover CA, Abbott DH, et al. Insulin and messenger ribonucleic acid expression of insulin receptor isoforms in ovarian follicles from nonhirsute ovulatory women and polycystic ovary syndrome patients. J Clin Endocrinol Metab. 2004;89(7):3561-3566. 15240646 10.1210/jc.2003-031888

[307] Cano F, García-Velasco JA, Millet A, Remohí J, Simón C, Pellicer A. Oocyte quality in polycystic ovaries revisited: identification of a particular subgroup of women. J Assist Reprod Genet. 1997;14(5):254-261. 9147238 10.1007/BF02765826 PMC3454720

[308] Marquard KL, Stephens SM, Jungheim ES, et al Polycystic ovary syndrome and maternal obesity affect oocyte size in in vitro fertilization/intracytoplasmic sperm injection cycles. Fertil Steril. 2011;95:2146-2149, 2149 e2141. 21071018 10.1016/j.fertnstert.2010.10.026 PMC3684964

[309] Vendola K, Zhou J, Wang J, Famuyiwa OA, Bievre M, Bondy CA. Androgens promote oocyte insulin-like growth factor I expression and initiation of follicle development in the primate ovary. Biol Reprod. 1999;61(2):353-357. 10411511 10.1095/biolreprod61.2.353

[310] Palomba S, Falbo A, Russo T, Tolino A, Orio F, Zullo F. Pregnancy in women with polycystic ovary syndrome: the effect of different phenotypes and features on obstetric and neonatal outcomes. Fertil Steril. 2010;94(5):1805-1811. 20004377 10.1016/j.fertnstert.2009.10.043

[311] Palomba S, Russo T, Falbo A, et al. Decidual endovascular trophoblast invasion in women with polycystic ovary syndrome: an experimental case-control study. J Clin Endocrinol Metab. 2012;97(7):2441-2449. 22508703 10.1210/jc.2012-1100

[312] Abbott DH, Bruns CR, Barnett DK, et al. Experimentally induced gestational androgen excess disrupts glucoregulation in rhesus monkey dams and their female offspring. Am J Physiol Endocrinol Metab. 2010;299(5):E741-E751. 20682841 10.1152/ajpendo.00058.2010 PMC2980359

[313] Batchuluun B, Al Rijjal D, Prentice KJ, et al. Elevated medium-chain acylcarnitines are associated with gestational diabetes mellitus and early progression to type 2 diabetes and induce pancreatic β-cell dysfunction. Diabetes. 2018;67(5):885-897. 29436377 10.2337/db17-1150 PMC5910003

[314] Novembri R, Funghi L, Voltolini C, et al. Placenta expresses anti-Müllerian hormone and its receptor: sex-related difference in fetal membranes. Placenta. 2015;36(7):731-737. 25972076 10.1016/j.placenta.2015.04.009

[315] Sun M, Maliqueo M, Benrick A, et al. Maternal androgen excess reduces placental and fetal weights, increases placental steroidogenesis, and leads to long-term health effects in their female offspring. Am J Physiol Endocrinol Metab. 2012;303(11):E1373-E1385. 23047983 10.1152/ajpendo.00421.2012

[316] Diamanti-Kandarakis E, Dunaif A. Insulin resistance and the polycystic ovary syndrome revisited: an update on mechanisms and implications. Endocr Rev. 2012;33(6):981-1030. 23065822 10.1210/er.2011-1034 PMC5393155

[317] Messer C, Boston R, Leroith D, et al. Pancreatic β-cell dysfunction in polycystic ovary syndrome: the role of metformin. Endocr Pract. 2012;18(5):685-693. 22548946 10.4158/EP11375.OR PMC3722880

[318] Harsha Varma S, Tirupati S, Pradeep TVS, Sarathi V, Kumar D. Insulin resistance and hyperandrogenemia independently predict nonalcoholic fatty liver disease in women with polycystic ovary syndrome. Diabetes Metab Syndr. 2019;13(2):1065-1069. 31336445 10.1016/j.dsx.2018.12.020

[319] Paschou SA, Polyzos SA, Anagnostis P, et al. Nonalcoholic fatty liver disease in women with polycystic ovary syndrome. Endocrine. 2020;67(1):1-8. 31538291 10.1007/s12020-019-02085-7

[320] Tantanavipas S, Vallibhakara O, Sobhonslidsuk A, et al. Abdominal obesity as a predictive factor of nonalcoholic fatty liver disease assessed by ultrasonography and transient elastography in polycystic ovary syndrome and healthy women. Biomed Res Int. 2019;2019:9047324. 31467918 10.1155/2019/9047324 PMC6699391

[321] Sarkar M, Terrault N, Chan W, et al. Polycystic ovary syndrome (PCOS) is associated with NASH severity and advanced fibrosis. Liver Int. 2020;40(2):355-359. 31627243 10.1111/liv.14279 PMC6980925

[322] Xu Y, Nedungadi TP, Zhu L, et al. Distinct hypothalamic neurons mediate estrogenic effects on energy homeostasis and reproduction. Cell Metab. 2011;14(4):453-465. 21982706 10.1016/j.cmet.2011.08.009 PMC3235745

[323] Sheppard KM, Padmanabhan V, Coolen LM, Lehman MN. Prenatal programming by testosterone of hypothalamic metabolic control neurones in the ewe. J Neuroendocrinol. 2011;23(5):401-411. 21418339 10.1111/j.1365-2826.2011.02126.x PMC3939689

[324] Evans JJ, Anderson GM. Balancing ovulation and anovulation: integration of the reproductive and energy balance axes by neuropeptides. Hum Reprod Update. 2012;18(3):313-332. 22442260 10.1093/humupd/dms004

[325] Coutinho E, Prescott M, Hessler S, Marshall C, Herbison A, Campbell RE. Activation of a classic hunger circuit slows luteinizing hormone pulsatility. Neuroendocrinology. 2019;10.1159/000504225. 10.1159/000504225 31630145

[326] Padilla SL, Qiu J, Nestor CC, et al. AgRP to Kiss1 neuron signaling links nutritional state and fertility. Proc Natl Acad Sci U S A. 2017;114(9):2413-2418. 28196880 10.1073/pnas.1621065114 PMC5338482

[327] Lu C, Cardoso RC, Puttabyatappa M, Padmanabhan V. Developmental programming: prenatal testosterone excess and insulin signaling disruptions in female sheep. Biol Reprod. 2016;94(5):113. 27053365 10.1095/biolreprod.115.136283 PMC4939741

[328] Cardoso RC, Veiga-Lopez A, Moeller J, et al. Developmental programming: impact of gestational steroid and metabolic milieus on adiposity and insulin sensitivity in prenatal testosterone-treated female sheep. Endocrinology. 2016;157(2):522-535. 26650569 10.1210/en.2015-1565 PMC4733129

[329] Nicol LE, O’Brien TD, Dumesic DA, Grogan T, Tarantal AF, Abbott DH. Abnormal infant islet morphology precedes insulin resistance in PCOS-like monkeys. PLoS One. 2014;9(9):e106527. 25207967 10.1371/journal.pone.0106527 PMC4160158

[330] Puttabyatappa M, Lu C, Martin JD, Chazenbalk G, Dumesic D, Padmanabhan V. Developmental programming: impact of prenatal testosterone excess on steroidal machinery and cell differentiation markers in visceral adipocytes of female sheep. Reprod Sci. 2018;25(7):1010-1023. 29237348 10.1177/1933719117746767 PMC6346350

[331] Keller E, Chazenbalk GD, Aguilera P, et al. Impaired preadipocyte differentiation into adipocytes in subcutaneous abdominal adipose of PCOS-like female rhesus monkeys. Endocrinology. 2014;155(7):2696-2703. 24735327 10.1210/en.2014-1050 PMC4060192

[332] Chazenbalk G, Singh P, Irge D, Shah A, Abbott DH, Dumesic DA. Androgens inhibit adipogenesis during human adipose stem cell commitment to preadipocyte formation. Steroids. 2013;78(9):920-926. 23707571 10.1016/j.steroids.2013.05.001 PMC3951890

[333] Xu N, Kwon S, Abbott DH, et al. Epigenetic mechanism underlying the development of polycystic ovary syndrome (PCOS)-like phenotypes in prenatally androgenized rhesus monkeys. PLoS One. 2011;6(11):e27286. 22076147 10.1371/journal.pone.0027286 PMC3208630

[334] Stepto NK, Moreno-Asso A, McIlvenna LC, Walters KA, Rodgers RJ. Molecular mechanisms of insulin resistance in polycystic ovary syndrome: unraveling the conundrum in skeletal muscle? J Clin Endocrinol Metab. 2019;104(11):5372-5381. 30938770 10.1210/jc.2019-00167

[335] de Zegher F, Reinehr T, Malpique R, Darendeliler F, López-Bermejo A, Ibáñez L. Reduced prenatal weight gain and/or augmented postnatal weight gain precedes polycystic ovary syndrome in adolescent girls. Obesity (Silver Spring). 2017;25(9):1486-1489. 28737293 10.1002/oby.21935

[336] Eisner JR, Dumesic DA, Kemnitz JW, Colman RJ, Abbott DH. Increased adiposity in female rhesus monkeys exposed to androgen excess during early gestation. Obes Res. 2003;11(2):279-286. 12582225 10.1038/oby.2003.42

[337] Bruns CM, Baum ST, Colman RJ, et al. Prenatal androgen excess negatively impacts body fat distribution in a nonhuman primate model of polycystic ovary syndrome. Int J Obes (Lond). 2007;31(10):1579-1585. 17471299 10.1038/sj.ijo.0803638 PMC2597033

[338] Wang ET, Ku IA, Shah SJ, et al. Polycystic ovary syndrome is associated with higher left ventricular mass index: the CARDIA women’s study. J Clin Endocrinol Metab. 2012;97(12):4656-4662. 23012389 10.1210/jc.2012-1597 PMC3591678

[339] Paradisi G, Steinberg HO, Hempfling A, et al. Polycystic ovary syndrome is associated with endothelial dysfunction. Circulation. 2001;103(10):1410-1415. 11245645 10.1161/01.cir.103.10.1410

[340] Soares GM, Vieira CS, Martins WP, et al. Increased arterial stiffness in nonobese women with polycystic ovary syndrome (PCOS) without comorbidities: one more characteristic inherent to the syndrome? Clin Endocrinol (Oxf). 2009;71(3):406-411. 19094071 10.1111/j.1365-2265.2008.03506.x

[341] Mannerås-Holm L, Baghaei F, Holm G, et al. Coagulation and fibrinolytic disturbances in women with polycystic ovary syndrome. J Clin Endocrinol Metab. 2011;96(4):1068-1076. 21252248 10.1210/jc.2010-2279

[342] Anderson SA, Barry JA, Hardiman PJ. Risk of coronary heart disease and risk of stroke in women with polycystic ovary syndrome: a systematic review and meta-analysis. Int J Cardiol. 2014;176(2):486-487. 25096551 10.1016/j.ijcard.2014.06.079

[343] Meun C, Franco OH, Dhana K, et al. High androgens in postmenopausal women and the risk for atherosclerosis and cardiovascular disease: the Rotterdam Study. J Clin Endocrinol Metab. 2018;103(4):1622-1630. 29408955 10.1210/jc.2017-02421

[344] Hurliman A, Keller Brown J, Maille N, Mandala M, Casson P, Osol G. Hyperandrogenism and insulin resistance, not changes in body weight, mediate the development of endothelial dysfunction in a female rat model of polycystic ovary syndrome (PCOS). Endocrinology. 2015;156(11):4071-4080. 26322372 10.1210/en.2015-1159

[345] Cesta CE, Månsson M, Palm C, Lichtenstein P, Iliadou AN, Landén M. Polycystic ovary syndrome and psychiatric disorders: co-morbidity and heritability in a nationwide Swedish cohort. Psychoneuroendocrinology. 2016;73:196-203. 27513883 10.1016/j.psyneuen.2016.08.005

[346] Cooney LG, Lee I, Sammel MD, Dokras A. High prevalence of moderate and severe depressive and anxiety symptoms in polycystic ovary syndrome: a systematic review and meta-analysis. Hum Reprod. 2017;32(5):1075-1091. 28333286 10.1093/humrep/dex044

[347] Dokras A, Stener-Victorin E, Yildiz BO, et al. Androgen Excess- Polycystic Ovary Syndrome Society: position statement on depression, anxiety, quality of life, and eating disorders in polycystic ovary syndrome. Fertil Steril. 2018;109(5):888-899. 29778388 10.1016/j.fertnstert.2018.01.038

[348] Ingudomnukul E, Baron-Cohen S, Wheelwright S, Knickmeyer R. Elevated rates of testosterone-related disorders in women with autism spectrum conditions. Horm Behav. 2007;51(5):597-604. 17462645 10.1016/j.yhbeh.2007.02.001

[349] Hergüner S, Harmancı H, Toy H. Attention deficit-hyperactivity disorder symptoms in women with polycystic ovary syndrome. Int J Psychiatry Med. 2015;50(3):317-325. 26449924 10.1177/0091217415610311

[350] Cherskov A, Pohl A, Allison C, Zhang H, Payne RA, Baron-Cohen S. Polycystic ovary syndrome and autism: a test of the prenatal sex steroid theory. Transl Psychiatry. 2018;8(1):136. 30065244 10.1038/s41398-018-0186-7 PMC6068102

[351] Thornton J, Zehr JL, Loose MD. Effects of prenatal androgens on rhesus monkeys: a model system to explore the organizational hypothesis in primates. Horm Behav. 2009;55(5):633-645. 19446080 10.1016/j.yhbeh.2009.03.015 PMC3146061

[352] Wallen K . Hormonal influences on sexually differentiated behavior in nonhuman primates. Front Neuroendocrinol. 2005;26(1):7-26. 15862182 10.1016/j.yfrne.2005.02.001

[353] Soleman RS, Kreukels BPC, Veltman DJ, et al Does polycystic ovary syndrome affect cognition? A functional magnetic resonance imaging study exploring working memory. Fertil Steril. 2016;105:1314-1321.e1311. 26878092 10.1016/j.fertnstert.2016.01.034

[354] Jarrett BY, Vantman N, Mergler RJ, et al. Dysglycemia, not altered sex steroid hormones, affects cognitive function in polycystic ovary syndrome. J Endocr Soc. 2019;3(10):1858-1868. 31583367 10.1210/js.2019-00112 PMC6767628

[355] Wang K, Xu F, Campbell SP, et al. Rapid actions of anti-Müllerian hormone in regulating synaptic transmission and long-term synaptic plasticity in the hippocampus. Faseb J. 2020;34(1):706-719. 31914642 10.1096/fj.201902217R PMC6956706

[356] Saben JL, Boudoures AL, Asghar Z, et al. Maternal metabolic syndrome programs mitochondrial dysfunction via germline changes across three generations. Cell Rep. 2016;16(1):1-8. 27320925 10.1016/j.celrep.2016.05.065 PMC4957639

[357] Whigham LD, Butz DE, Dashti H, et al. Metabolic evidence of diminished lipid oxidation in women with polycystic ovary syndrome. Curr Metabolomics. 2014;2(4):269-278. 24765590 10.2174/2213235X01666131203230512 PMC3994884

[358] Roland AV, Moenter SM. Prenatal androgenization of female mice programs an increase in firing activity of gonadotropin-releasing hormone (GnRH) neurons that is reversed by metformin treatment in adulthood. Endocrinology. 2011;152(2):618-628. 21159854 10.1210/en.2010-0823 PMC3037157

[359] Eagleson CA, Gingrich MB, Pastor CL, et al. Polycystic ovarian syndrome: evidence that flutamide restores sensitivity of the gonadotropin-releasing hormone pulse generator to inhibition by estradiol and progesterone. J Clin Endocrinol Metab. 2000;85(11):4047-4052. 11095431 10.1210/jcem.85.11.6992

[360] Ibáñez L, Díaz M, Sebastiani G, Marcos MV, López-Bermejo A, de Zegher F. Oral contraception vs insulin sensitization for 18 months in nonobese adolescents with androgen excess: posttreatment differences in C-reactive protein, intima-media thickness, visceral adiposity, insulin sensitivity, and menstrual regularity. J Clin Endocrinol Metab. 2013;98(5):E902-E907. 23547047 10.1210/jc.2013-1041

[361] Ezeh U, Huang A, Landay M, Azziz R. Long-term response of hirsutism and other hyperandrogenic symptoms to combination therapy in polycystic ovary syndrome. J Womens Health (Larchmt). 2018;27(7):892-902. 29878857 10.1089/jwh.2017.6833 PMC6065519

